# Supplementary material for: The Parallel Structure–Activity Relationship Screening of Three Compounds Identifies the Common Agonist Pharmacophore of Pyrrolidine Bis-Cyclic Guanidine Melanocortin-3 Receptor (MC3R) Small-Molecule Ligands
Source: Int J Mol Sci. 2023 Jun 14;24(12):10145. doi: 10.3390/ijms241210145 (PMC10299128; doi:10.3390/ijms241210145)
Supplement: Supplementary file 1 [file ijms-24-10145-s001.zip › ijms-2352417-supplementary.pdf]

SUPPLEMENTAL MATERIALS

The Parallel Structure–Activity Relationship Screening of Three Compounds Identifies the Common Agonist Pharmacophore of Pyrrolidine Bis-Cyclic Guanidine Melanocortin-3 Receptor (MC3R) Small Molecule Ligands

Mark D. Ericson [1], Katie T. Freeman [1], Travis M. LaVoi [2], Haley M. Donow [2], Radleigh G. Santos [3], Marc A. Giulianotti [2], Clemencia Pinilla [2], Richard A. Houghten [2] and Carrie Haskell-Luevano [1,*]

[1] Department of Medicinal Chemistry & Institute for Translational Neuroscience, University of Minnesota, Minneapolis, MN 55455, USA

[2] Center for Translational Science, Florida International University, Port St. Lucie, FL 34987, USA

[3] Department of Mathematics, Nova Southeastern University, Fort Lauderdale, FL 33314, USA

* Correspondence: chaskell@umn.edu

Compound purification and characterization. All reagents were commercially available and used without further purification. Pyrrolidine bis-cyclic guanidine compounds were synthesized using Scheme 1. The final compounds were purified using preparative HPLC with a dual pump Shimadzu LC-20AB system equipped with a Luna C18 preparative column (21.5 x 150 mm, 5 micron) at λ = 214 nm, with a mobile phase of (A) H_2O (+0.1% formic acid)/(B) acetonitrile (ACN) (+0.1% formic acid) at a flow rate of 15 mL/min; gradients varied by compound based on hydrophobicity. 1H NMR and 13C NMR spectra were recorded in DMSO-d6 on a Bruker Ascend 400 MHz spectrometer at 400.14 and 100.62 MHz, respectively. The purities of synthesized compounds were assessed by liquid chromatography and mass spectrometry on a Shimadzu LCMS-2020 instrument with ESI Mass Spec and SPD-20A Liquid Chromatograph with a mobile phase of (A) H_2O (+0.1% formic acid)/(B) ACN (+0.1% formic acid) (5-95% over 6 min with a 4 min rinse).

2718.001 (S)-1-((S)-3-((4-(tert-butyl)cyclohexyl)methyl)-2-imino-1-((R)-1-((S)-2-(((R)-2-imino-5-isobutylimidazolidin-1-yl)methyl)pyrrolidin-1-yl)-3-phenylpropan-2-yl)imidazolidin-4-yl)ethanol. Compound 2718.001 was synthesized using the following reagents: (100mg) MBHA resin starting material, Boc-D-Leucine-OH (R_1), Boc-L-Proline-OH (R_2), Boc-D-Phenylalanine-OH (R_3), Boc-D-Threonine(Bzl)-OH (R_4), and 4-tert-Butylcyclohexancecarboxylic acid (R_5). The final crude product was purified using HPLC as described above, with a gradient of (B) 0/2, 2/2, 20/27, 25/30, 27/98, 30/98. **Isolated Mass** 7.9mg, **^{1}H NMR** (400 MHz, DMSO-d6) ppm 8.62 (br. s., 2 H) 7.23 (br. s., 2 H) 4.69 (br. s., 1 H) 3.94 (br. s., 3 H) 3.79 (br. s., 1 H) 3.74 (br. s., 1 H) 3.68 (br. s., 1 H) 3.60 (br. s., 2 H) 3.39 - 3.55 (m, 2 H) 3.30 (br. s., 2 H) 3.11 (br. s., 1 H) 2.99 (d, J=13.20 Hz, 3 H) 2.61 - 2.85 (m, 2 H) 2.46 (d, J=11.49 Hz, 1 H) 2.34 (br. s., 1 H) 1.79 - 1.99 (m, 2 H) 1.69 (br. s., 2 H) 1.36 - 1.63 (m, 7 H) 1.26 (br. s., 2 H) 1.15 (br. s., 3 H) 1.00 (br. s., 4 H) 0.86 - 0.97 (m, 5 H) 0.83 (br. s., 9 H) **m/z** calcd $C_{37}H_{63}N_7O$ $[M+H]^+$ 622.5, found 622.55 (MS ESI) **Purity** LC-MS: >96% (214 nm, peak area).

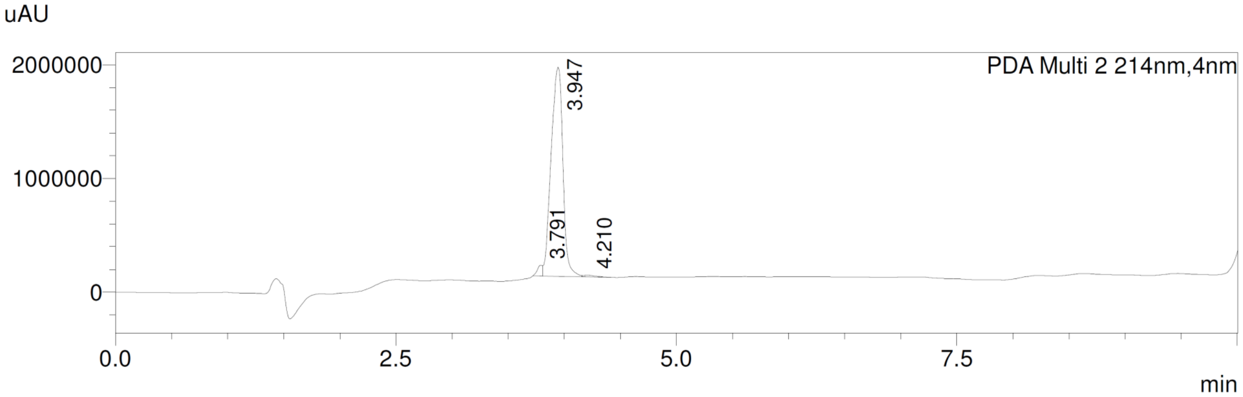

PDA Ch2 214nm

Peak#	Ret. Time	Area	Height	Area%	Conc.
1	3.791	317255	95200	2.297	2.297
2	3.947	13371844	1840711	96.820	96.820
3	4.210	121955	16020	0.883	0.883
Total		13811055	1951931	100.000	

2718.002 (S)-1-((S)-3-((4-(tert-butyl)cyclohexyl)methyl)-1-((R)-1-cyclohexyl-3-((S)-2-(((R)-2-imino-5-isobutylimidazolidin-1-yl)methyl)pyrrolidin-1-yl)propan-2-yl)-2-iminoimidazolidin-4-yl)ethanol. Compound 2718.002 was synthesized using the following reagents: (100mg) MBHA resin starting material, Boc-D-Leucine-OH (R_1), Boc-L-Proline-OH (R_2), Boc-D-Cyclohexylalanine-OH (R_3), Boc-D-Threonine(Bzl)-OH (R_4), and 4-tert-Butylcyclohexancecarboxylic acid (R_5). The final crude product was purified using HPLC as described above, with a gradient of (B) 0/2, 2/2, 20/27, 25/30, 27/98, 30/98. **Isolated Mass** 7.9mg, **^{1}H NMR** (400 MHz, DMSO-*d*6) d ppm 8.23 (br. s., 1 H) 3.99 (br. s., 1 H) 3.71 - 3.95 (m, 3 H) 3.66 (t, *J*=9.29 Hz, 1 H) 3.43 - 3.58 (m, 3 H) 3.38 (br. s., 6 H) 3.09 - 3.34 (m, 7 H) 3.04 (d, *J*=3.42 Hz, 1 H) 2.89 (br. s., 1 H) 2.74 (d, *J*=3.91 Hz, 1 H) 2.53 (br. s., 1 H) 2.10 - 2.35 (m, 2 H) 1.76 - 1.94 (m, 2 H) 1.63 - 1.76 (m, 4 H) 1.53 - 1.63 (m, 4 H) 1.31 - 1.53 (m, 6 H) 1.17 - 1.31 (m, 2 H) 1.12 (d, *J*=7.83 Hz, 3 H) 0.84 (s, 8 H) 0.79 - 1.09 (m, 11 H) **m/z** calcd $C_{37}H_{69}N_7O$ $[M+H]^+$ 628.56, found 628.95 (MS ESI) **Purity** LC-MS: >94% (214 nm, peak area).

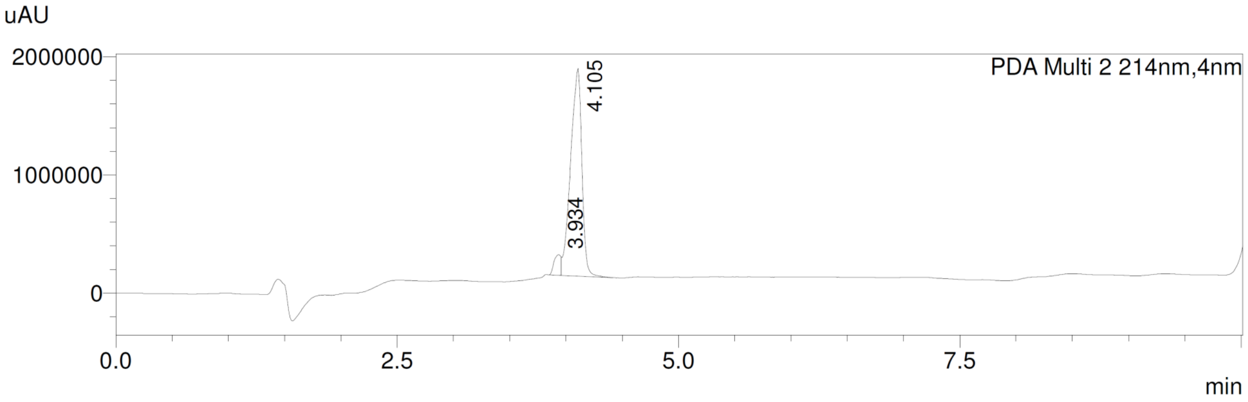

PDA Ch2 214nm

Peak#	Ret. Time	Area	Height	Area%	Conc.
1	3.934	768969	176689	5.901	5.901
2	4.105	12261866	1760305	94.099	94.099
Total		13030834	1936995	100.000	

2718.003 (R)-3-((4-(tert-butyl)cyclohexyl)methyl)-1-((R)-1-cyclohexyl-3-((S)-2-(((R)-2-imino-5-isopropylimidazolidin-1-yl)methyl)pyrrolidin-1-yl)propan-2-yl)-4-propylimidazolidin-2-imine. Compound 2718.003 was synthesized using the following reagents: (100mg) MBHA resin starting material, Boc-D-Valine-OH (R_1), Boc-L-Proline-OH (R_2), Boc-D-Cyclohexylalanine-OH (R_3), Boc-D-Norvaline-OH (R_4), and 4-tert-Butylcyclohexancecarboxylic acid (R_5). The final crude product was purified using HPLC as described above, with a gradient of (B) 0/2, 2/2, 20/27, 25/30, 27/98, 30/98. **Isolated Mass** 7.3mg, **^{1}H NMR** (400 MHz, DMSO-d6) d ppm 10.42 (br. s., 1 H) 8.35 (br. s., 1 H) 3.78 - 4.03 (m, 4 H) 3.41 - 3.71 (m, 7 H) 3.19 - 3.41 (m, 4 H) 3.11 - 3.17 (m, 1 H) 2.78 - 3.08 (m, 3 H) 2.22 - 2.48 (m, 2 H) 2.18 (dt, J=6.76, 3.29 Hz, 1 H) 2.08 (br. s., 1 H) 1.64 - 1.93 (m, 7 H) 1.61 (br. s., 2 H) 1.34 - 1.55 (m, 6 H) 1.05 - 1.32 (m, 7 H) 0.79 - 1.05 (m, 17 H) 0.74 (d, J=6.72 Hz, 3 H) **m/z** calcd $C_{37}H_{69}N_7$ $[M+H]^+$ 612.56, found 613.0 (MS ESI) **Purity** LC-MS: >99% (214 nm, peak area).

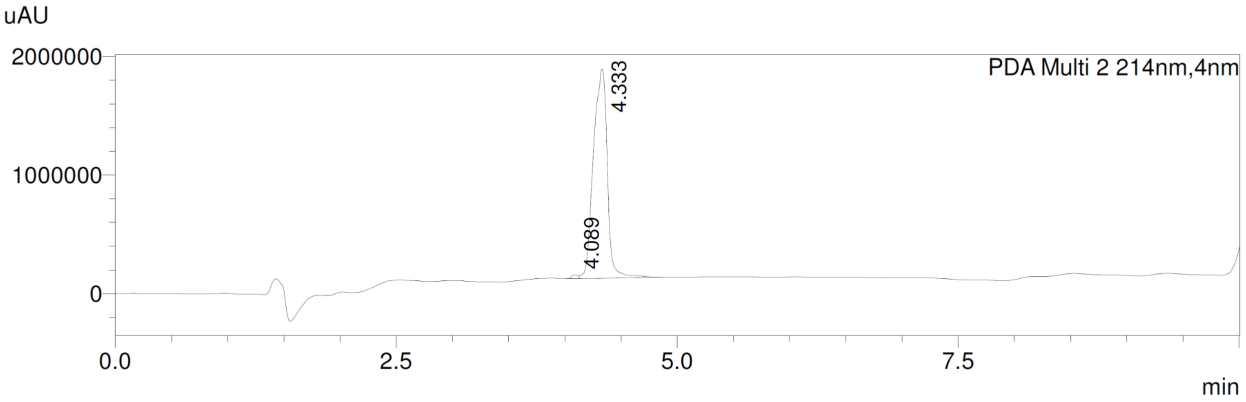

PDA Ch2 214nm

Peak#	Ret. Time	Area	Height	Area%	Conc.
1	4.089	130945	30839	0.856	0.856
2	4.333	15162360	1769533	99.144	99.144
Total		15293305	1800372	100.000	

2718.004 (S)-1-((S)-3-ethyl-2-imino-1-((R)-1-((S)-2-(((R)-2-imino-5-isobutylimidazolidin-1-yl)methyl)pyrrolidin-1-yl)-3-phenylpropan-2-yl)imidazolidin-4-yl)ethanol. Compound 2718.004 was synthesized using the following reagents: (100mg) MBHA resin starting material, Boc-D-Leucine-OH (R_1), Boc-L-Proline-OH (R_2), Boc-D-Phenylalanine-OH (R_3), Boc-D-Threonine(Bzl)-OH (R_4), and Acetic Anhydride (R_5). The final crude product was purified using HPLC as described above, with a gradient of (B) 0/2, 2/2, 20/26, 25/29, 27/98, 30/98. **Isolated Mass** 7.1mg, **^{1}H NMR** (400 MHz, DMSO-d 6) d ppm 8.44 (br. s., 1 H) 7.17 - 7.36 (m, 4 H) 4.23 - 4.33 (m, 1 H) 3.77 - 4.02 (m, 2 H) 3.54 - 3.77 (m, 3 H) 3.17 - 3.47 (m, 13 H) 2.92 - 3.08 (m, 2 H) 2.65 - 2.92 (m, 3 H) 2.29 - 2.48 (m, 1 H) 2.23 (q, J=8.27 Hz, 1 H) 1.75 - 1.92 (m, 1 H) 1.52 - 1.75 (m, 3 H) 1.29 - 1.52 (m, 2 H) 1.03 (d, J=6.24 Hz, 2 H) 0.93 (dd, J=18.52, 5.93 Hz, 5 H) 0.83 (t, J=7.03 Hz, 2 H) **m/z** calcd $C_{28}H_{47}N_7O$ $[M+H]^+$ 498.4, found 498.3 (MS ESI) **Purity** LC-MS: >99% (214 nm, peak area).

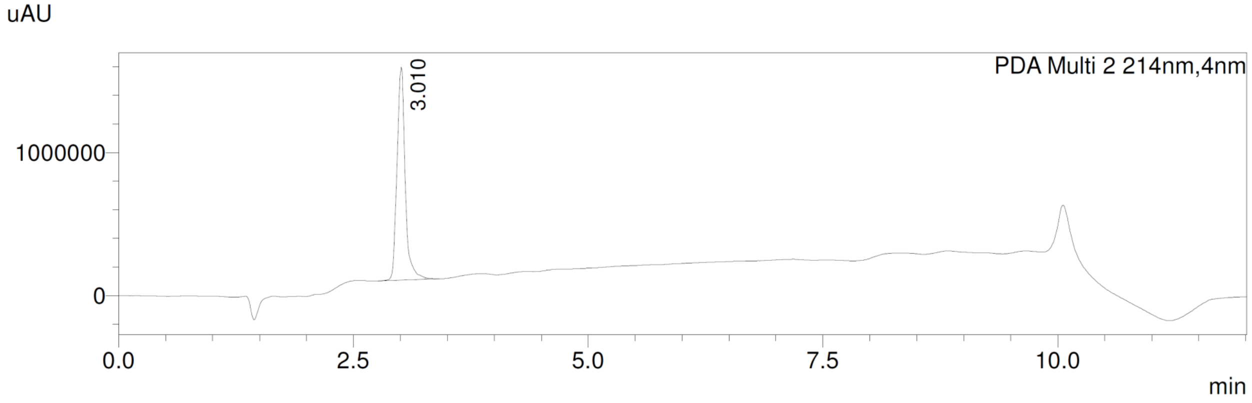

PDA Ch2 214nm

Peak#	Ret. Time	Area	Height	Area%	Conc.
1	3.010	9095706	1489523	100.000	100.000
Total		9095706	1489523	100.000	

2718.006 (R)-1-(((S)-1-((R)-2-(2-iminoimidazolidin-1-yl)-3-phenylpropyl)pyrrolidin-2-yl)methyl)-5-isobutylimidazolidin-2-imine. Compound 2718.006 was synthesized using the following reagents: (100mg) MBHA resin starting material, Boc-D-Leucine-OH (R_1), Boc-L-Proline-OH (R_2), Boc-D-Phenylalanine-OH (R_3), and Boc-Glycine-OH (R_4). The final crude product was purified using HPLC as described above, with a gradient of (B) 0/2, 2/2, 20/26, 25/29, 27/98, 30/98. **Isolated Mass** 6.3mg, **^{1}H NMR** (400 MHz, DMSO-d6) d ppm 10.30 (br. s., 1 H) 9.94 (br. s., 1 H) 8.39 (br. s., 2 H) 7.21 - 7.34 (m, 4 H) 4.17 (br. s., 1 H) 3.86 - 4.05 (m, 2H) 3.59 - 3.86 (m, 4 H) 3.51 (d, J=5.14 Hz, 1 H) 3.45 - 3.56 (m, 1 H) 3.38 - 3.44 (m, 1 H) 3.13 - 3.33 (m, 3 H) 3.00 - 3.12 (m, 1 H) 2.72 - 2.99 (m, 3H) 2.22 - 2.48 (m, 2 H) 1.76 - 1.91 (m, 1 H) 1.52 - 1.75 (m, 3 H) 1.24 - 1.50 (m, 2 H) 0.80 - 0.98 (m, 6 H) **m/z** calcd $C_{24}H_{39}N_7$ $[M+H]^+$ 426.3, found 426.25 (MS ESI) **Purity** LC-MS: >99% (214 nm, peak area).

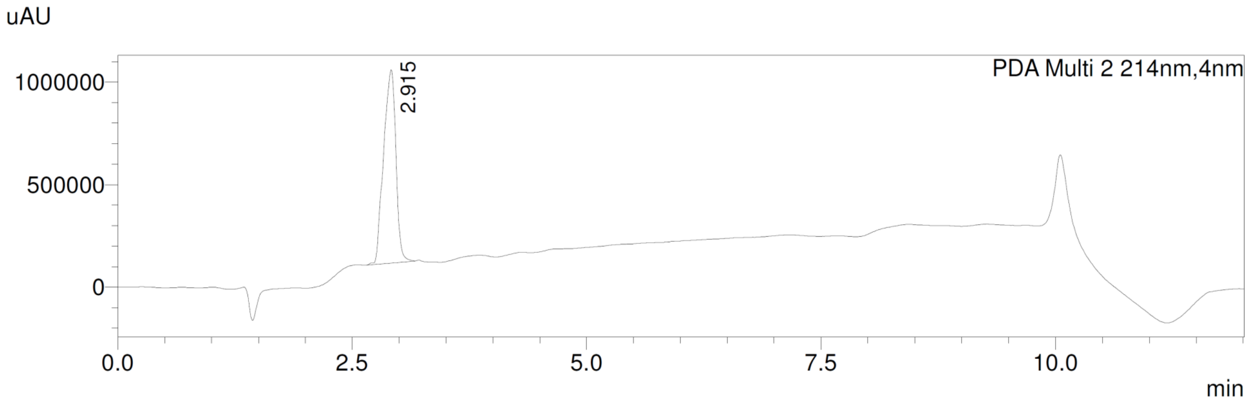

PDA Ch2 214nm

Peak#	Ret. Time	Area	Height	Area%	Conc.
1	2.915	8585807	943626	100.000	100.000
Total		8585807	943626	100.000	

2718.010 (S)-1-((S)-1-((R)-1-cyclohexyl-3-((S)-2-(((R)-2-imino-5-isobutylimidazolidin-1-yl)methyl)pyrrolidin-1-yl)propan-2-yl)-3-ethyl-2-iminoimidazolidin-4-yl)ethanol. Compound 2718.010 was synthesized using the following reagents: (100mg) MBHA resin starting material, Boc-D-Leucine-OH (R_1), Boc-L-Proline-OH (R_2), Boc-D-Cyclohexylalanine-OH (R_3), Boc-D-Threonine(Bzl)-OH (R_4), and Acetic Anhydride (R_5). The final crude product was purified using HPLC as described above, with a gradient of (B) 0/2, 2/2, 20/26, 25/29, 27/98, 30/98. **Isolated Mass** 5.8mg, **^{1}H NMR** (400 MHz, DMSO-d6) d ppm 10.35 (br. s., 1 H) 8.27 (br. s., 1 H) 3.78 - 4.06 (m, 7 H) 3.43 - 3.71 (m, 5 H) 3.09 - 3.31 (m, 4 H) 3.02 - 3.08 (m, 1 H) 2.79 - 2.94 (m, 1 H) 2.72 - 2.79 (m, 1 H) 2.28 (d, J=9.05 Hz, 2 H) 1.75 - 1.89 (m, 2 H) 1.52 - 1.74 (m, 8 H) 1.26 - 1.48 (m, 4 H) 1.12 - 1.23 (m, 3 H) 1.03 - 1.12 (m, 6 H) 0.79 - 1.02 (m, 8 H) **m/z** calcd $C_{28}H_{53}N_7O$ $[M+H]^+$ 504.4, found 504.35 (MS ESI) **Purity** LC-MS: >99% (214 nm, peak area).

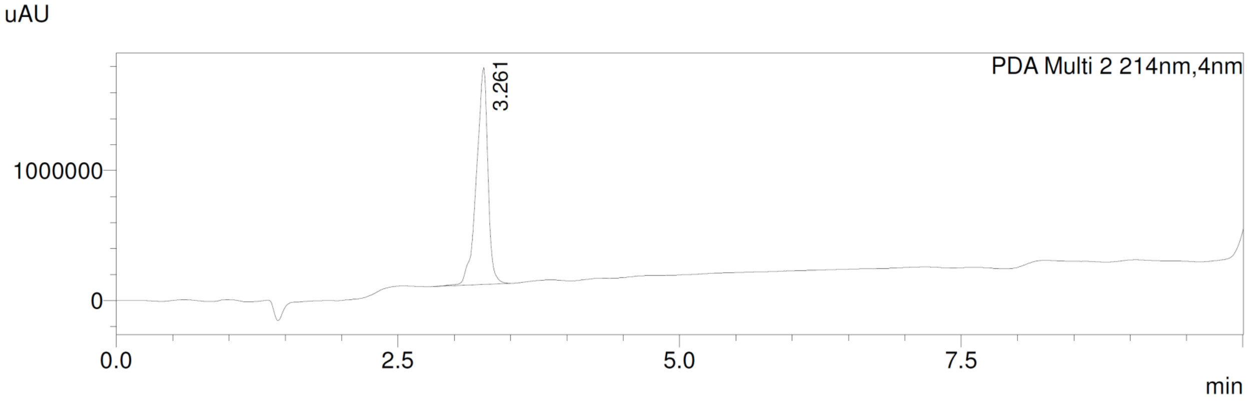

PDA Ch2 214nm

Peak#	Ret. Time	Area	Height	Area%	Conc.
1	3.261	11822142	1673131	100.000	100.000
Total		11822142	1673131	100.000	

2718.011 (S)-1-((S)-1-((R)-1-cyclohexyl-3-((S)-2-(((R)-2-imino-5-isobutylimidazolidin-1-yl)methyl)pyrrolidin-1-yl)propan-2-yl)-2-iminoimidazolidin-4-yl)ethanol. Compound 2718.011 was synthesized using the following reagents: (100mg) MBHA resin starting material, Boc-D-Leucine-OH (R_1), Boc-L-Proline-OH (R_2), Boc-D-Cyclohexylalanine-OH (R_3), and Boc-D-Threonine(Bzl)-OH (R_4). The final crude product was purified using HPLC as described above, with a gradient of (B) 0/2, 2/2, 20/26, 25/29, 27/98, 30/98. **Isolated Mass** 5.7mg, **^{1}H NMR** (400 MHz, DMSO-d6) d ppm 10.05 (br. s., 1 H) 9.98 (br. s., 1 H) 8.65 (br. s., 2 H) 4.23 (br. s., 1 H) 4.01 (br. s., 1 H) 3.79 - 3.95 (m, 3 H) 3.64 - 3.76 (m, 3 H) 3.48 - 3.61 (m, 3 H) 3.04 - 3.30 (m, 5 H) 2.71 - 2.94 (m, 2 H) 2.17 - 2.35 (m, 2 H) 1.62 - 1.88 (m, 6 H) 1.52 - 1.62 (m, 3 H) 1.33 - 1.49 (m, 3 H) 1.03 - 1.30 (m, 7 H) 0.80 - 1.00 (m, 8 H) **m/z** calcd $C_{26}H_{49}N_7O$ $[M+H]^+$ 476.4, found 476.65 (MS ESI) **Purity** LC-MS: >95% (214 nm, peak area).

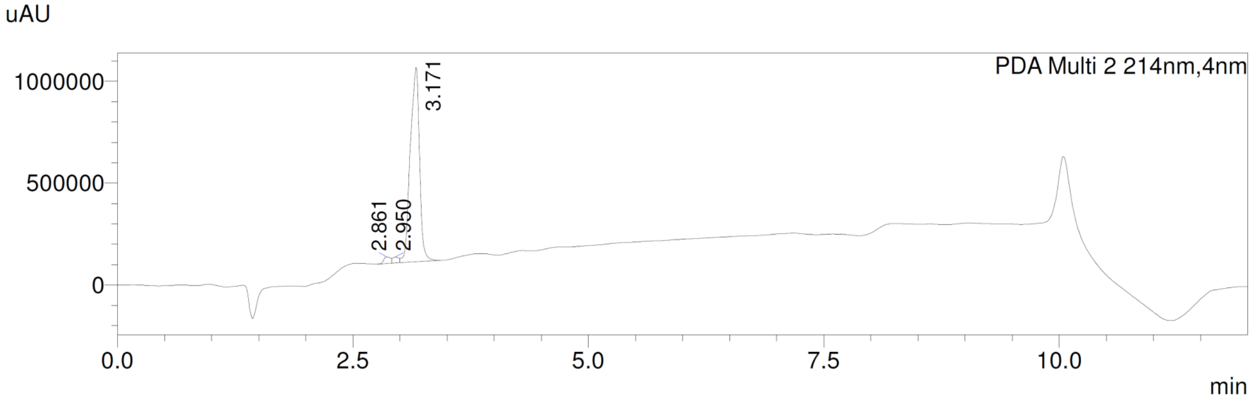

PDA Ch2 214nm

Peak#	Ret. Time	Area	Height	Area%	Conc.
1	2.861	168560	32582	2.474	2.474
2	2.950	132609	28825	1.946	1.946
3	3.171	6512817	955412	95.580	95.580
Total		6813986	1016818	100.000	

2718.012 (R)-1-(((S)-1-((R)-3-cyclohexyl-2-(2-iminoimidazolidin-1-yl)propyl)pyrrolidin-2-yl)methyl)-5-isobutylimidazolidin-2-imine. Compound 2718.012 was synthesized using the following reagents: (100mg) MBHA resin starting material, Boc-D-Leucine-OH (R_1), Boc-L-Proline-OH (R_2), Boc-D-Cyclohexylalanine-OH (R_3), and Boc-Glycine-OH (R_4). The final crude product was purified using HPLC as described above, with a gradient of (B) 0/2, 2/2, 20/26, 25/29, 27/98, 30/98. **Isolated Mass** 6.3mg, **^{1}H NMR** (400 MHz, DMSO-d6) d ppm 10.19 (br. s., 1 H) 8.40 (br. s., 2 H) 8.27 (br. s., 1 H) 3.78 - 4.06 (m, 3 H) 3.43 - 3.71 (m, 9 H) 3.08 - 3.31 (m, 4 H) 2.71 - 2.87 (m, 2 H) 2.17 - 2.35 (m, 2 H) 1.75 - 1.91 (m, 2 H) 1.54 - 1.75 (m, 8 H) 1.31 - 1.46 (m, 3 H) 1.08 - 1.28 (m, 5 H) 0.80 - 1.00 (m, 8 H) **m/z** calcd $C_{24}H_{45}N_7$ $[M+H]^+$ 432.4, found 432.25 (MS ESI) **Purity** LC-MS: >95% (214 nm, peak area).

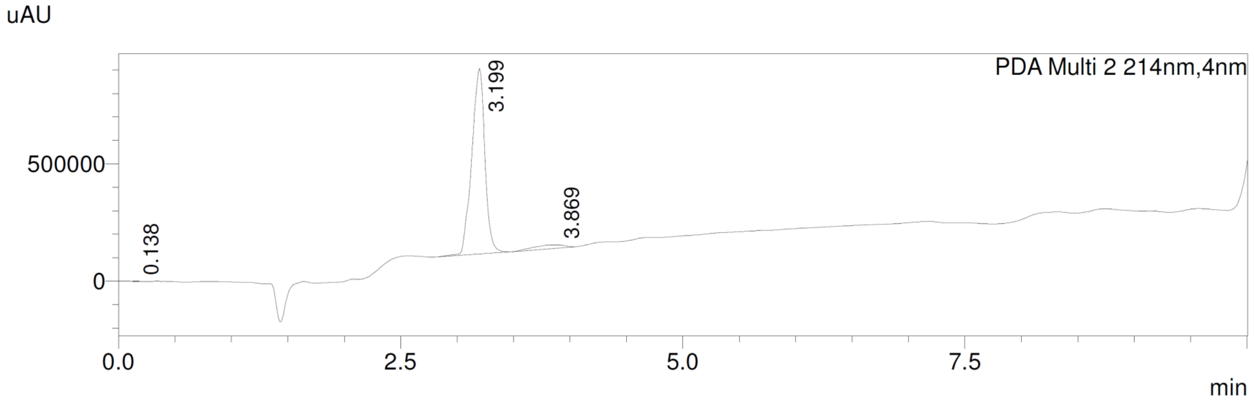

PDA Ch2 214nm

Peak#	Ret. Time	Area	Height	Area%	Conc.
1	0.138	1464	442	0.022	0.022
2	3.199	6401103	792014	95.196	95.196
3	3.869	321539	15306	4.782	4.782
Total		6724107	807762	100.000	

2718.014 (R)-1-((R)-1-cyclohexyl-3-((S)-2-(((R)-2-imino-5-isopropylimidazolidin-1-yl)methyl)pyrrolidin-1-yl)propan-2-yl)-3-ethyl-4-propylimidazolidin-2-imine. Compound 2718.014 was synthesized using the following reagents: (100mg) MBHA resin starting material, Boc-D-Valine-OH (R_1), Boc-L-Proline-OH (R_2), Boc-D-Cyclohexylalanine-OH (R_3), Boc-D-Norvaline-OH (R_4), and Acetic Anhydride (R_5). The final crude product was purified using HPLC as described above, with a gradient of (B) 0/2, 2/2, 20/26, 25/29, 27/98, 30/98. **Isolated Mass** 6.6mg, **^{1}H NMR** (400 MHz, DMSO-d6) d ppm 8.43 (br. s., 1 H) 3.75 - 4.05 (m, 3 H) 3.55 - 3.63 (m, 2 H) 3.27 - 3.52 (m, 10 H) 3.18 - 3.23 (m, 1 H) 3.08 - 3.15 (m, 1 H) 2.98 (dd, J=14.37, 4.10 Hz, 1 H) 2.76 - 2.92 (m, 2 H) 2.53 (br. s., 1 H) 2.23 - 2.48 (m, 2 H) 2.07 - 2.23 (m, 1 H) 1.93 - 2.07 (m, 1 H) 1.57 - 1.87 (m, 7 H) 1.19 - 1.49 (m, 6 H) 1.11 - 1.19 (m, 2 H) 1.05 (t, J=7.03 Hz, 3 H) 0.81 - 1.01 (m, 6 H) 0.74 (d, J=6.72 Hz, 2 H) **m/z** calcd $C_{28}H_{53}N_7$ $[M+H]^+$ 488.4, found 488.8 (MS ESI) **Purity** LC-MS: >99% (214 nm, peak area).

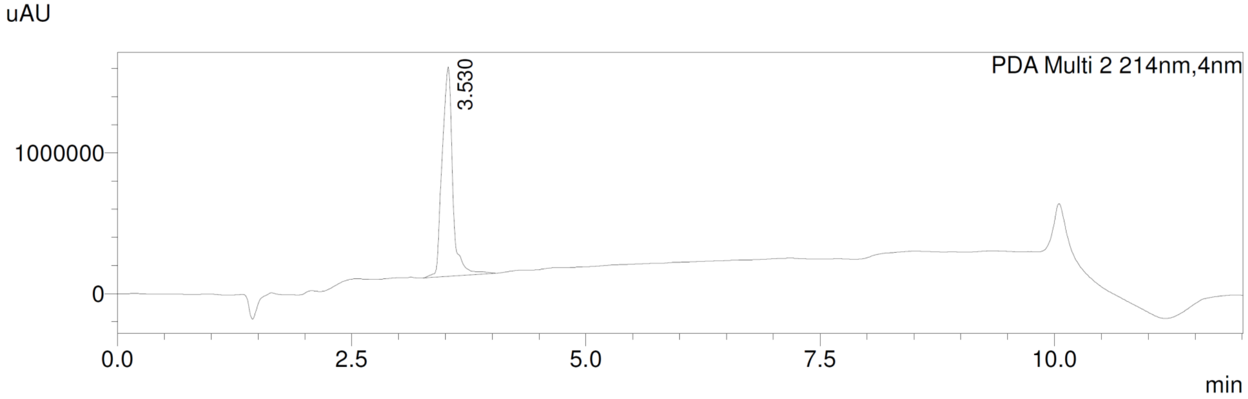

PDA Ch2 214nm

Peak#	Ret. Time	Area	Height	Area%	Conc.
1	3.530	11660131	1489871	100.000	100.000
Total		11660131	1489871	100.000	

2718.015 (R)-1-((R)-1-cyclohexyl-3-((S)-2-(((R)-2-imino-5-isopropylimidazolidin-1-yl)methyl)pyrrolidin-1-yl)propan-2-yl)-4-propylimidazolidin-2-imine. Compound 2718.015 was synthesized using the following reagents: (100mg) MBHA resin starting material, Boc-D-Valine-OH (R_1), Boc-L-Proline-OH (R_2), Boc-D-Cyclohexylalanine-OH (R_3), and Boc-D-Norvaline-OH (R_4). The final crude product was purified using HPLC as described above, with a gradient of (B) 0/2, 2/2, 20/26, 25/29, 27/98, 30/98. **Isolated Mass** 5.8mg, **^{1}H NMR** (400 MHz, DMSO-d6) d ppm 10.52 (br. s., 1 H) 10.14 (br. s., 1 H) 8.70 (br. s., 1 H) 8.33 (br. s., 2 H) 3.83 - 4.09 (m, 2 H) 3.69 - 3.80 (m, 2 H) 3.31 - 3.60 (m, 6 H) 3.18 - 3.29 (m, 2 H) 2.95 - 3.14 (m, 2 H) 2.72 - 2.94 (m, 2 H) 2.46 (br. s., 1 H) 2.00 - 2.27 (m, 2 H) 1.52 - 1.81 (m, 6 H) 1.30 - 1.52 (m, 4 H) 1.01 - 1.27 (m, 5 H) 0.73 - 0.91 (m, 7 H) 0.67 (d, J=6.72 Hz, 3 H) **m/z** calcd $C_{26}H_{49}N_7$ $[M+H]^+$ 460.4, found 460.7 (MS ESI) **Purity** LC-MS: >99% (214 nm, peak area).

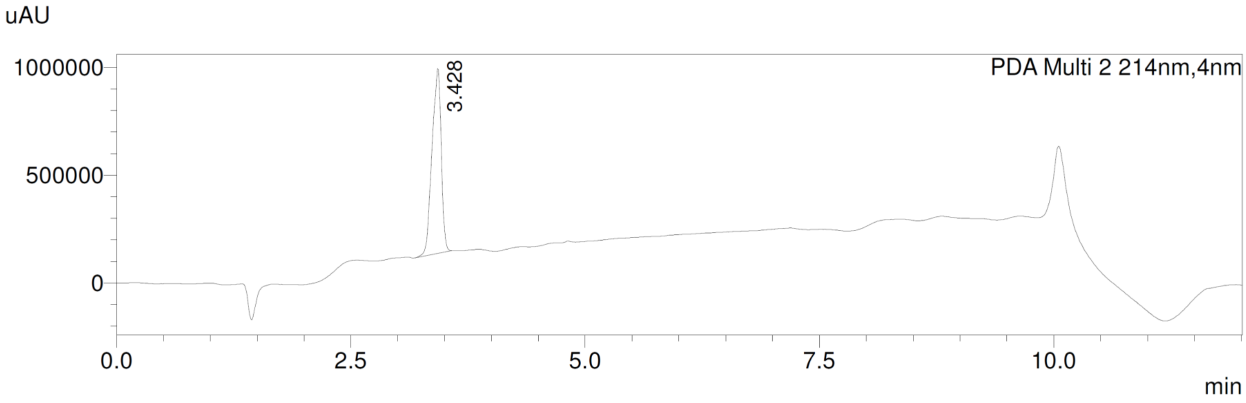

PDA Ch2 214nm

Peak#	Ret. Time	Area	Height	Area%	Conc.
1	3.428	5887893	858040	100.000	100.000
Total		5887893	858040	100.000	

2718.016 (R)-1-(((S)-1-((R)-3-cyclohexyl-2-(2-iminoimidazolidin-1-yl)propyl)pyrrolidin-2-yl)methyl)-5-isopropylimidazolidin-2-imine. Compound 2718.016 was synthesized using the following reagents: (100mg) MBHA resin starting material, Boc-D-Valine-OH (R_1), Boc-L-Proline-OH (R_2), Boc-D-Cyclohexylalanine-OH (R_3), and Boc-Glycine-OH (R_4). The final crude product was purified using HPLC as described above, with a gradient of (B) 0/2, 2/2, 20/26, 25/29, 27/98, 30/98. **Isolated Mass** 5.9mg, **^{1}H NMR** (400 MHz, DMSO-d6) d ppm 9.80 (br. s., 1 H) 8.70 (br. s., 2 H) 4.08 - 4.37 (m, 1 H) 3.70 - 3.98 (m, 5 H) 3.41 - 3.69 (m, 6 H) 3.26 - 3.36 (m, 2 H) 3.04 - 3.20 (m, 2 H) 2.78 - 2.91 (m, 2 H) 2.53 (br. s., 1 H) 2.10 - 2.34 (m, 2 H) 1.78 - 1.89 (m, 2 H) 1.58 - 1.76 (m, 5 H) 1.35 - 1.56 (m, 2 H) 1.07 - 1.30 (m, 4 H) 0.90 - 1.02 (m, 1 H) 0.86 (d, J=6.85 Hz, 3 H) 0.76 (d, J=6.72 Hz, 3 H) **m/z** calcd $C_{23}H_{43}N_7$ $[M+H]^+$ 418.4, found 418.25 (MS ESI) **Purity** LC-MS: >99% (214 nm, peak area).

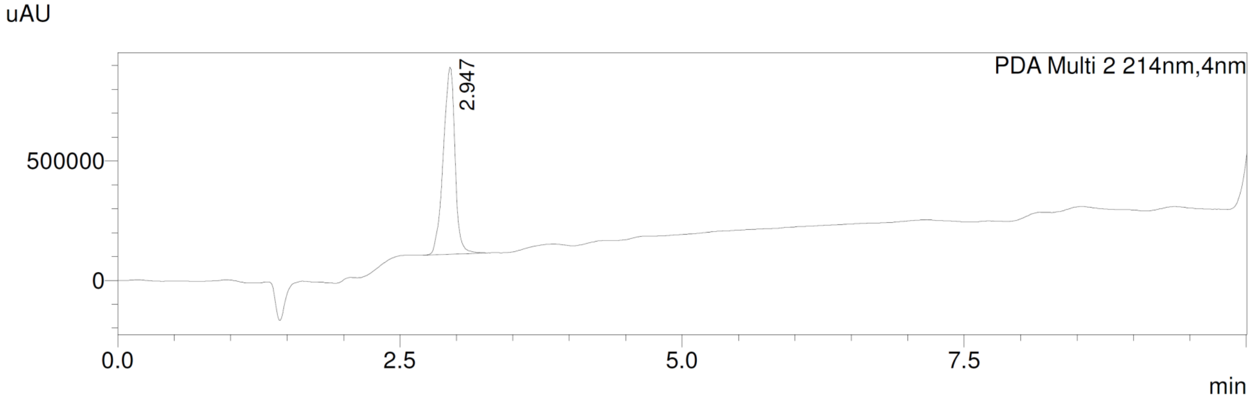

PDA Ch2 214nm

Peak#	Ret. Time	Area	Height	Area%	Conc.
1	2.947	5667516	785224	100.000	100.000
Total		5667516	785224	100.000	

2718.020 (S)-1-((S)-3-((4-(tert-butyl)cyclohexyl)methyl)-2-iminoimidazolidin-4-yl)ethanol. Compound 2718.020 was synthesized using the following reagents: (100mg) MBHA resin starting material, Boc-D-Threonine(Bzl)-OH (R_4), and 4-tert-Butylcyclohexancecarboxylic acid (R_5). The final crude product was purified using HPLC as described above, with a gradient of (B) 0/2, 2/2, 20/27, 25/30, 27/98, 30/98. **Isolated Mass** 6.8mg, **^{1}H NMR** (400 MHz, DMSO-d6) d ppm 8.62 (br. s., 1 H) 3.72 - 4.00 (m, 2 H) 3.46 - 3.72 (m, 2 H) 3.26 - 3.46 (m, 4 H) 3.16 (dd, J=14.55, 5.99 Hz, 1 H) 2.12 (br. s., 1 H) 1.59 (br. s., 2 H) 1.30 - 1.56 (m, 4 H) 1.06 - 1.30 (m, 1 H) 0.88 - 1.06 (m, 4 H) 0.58 - 0.88 (m, 8 H) **m/z** calcd $C_{16}H_{31}N_3O$ $[M+H]^+$ 282.3, found 282.55 (MS ESI) **Purity** LC-MS: >97% (214 nm, peak area).

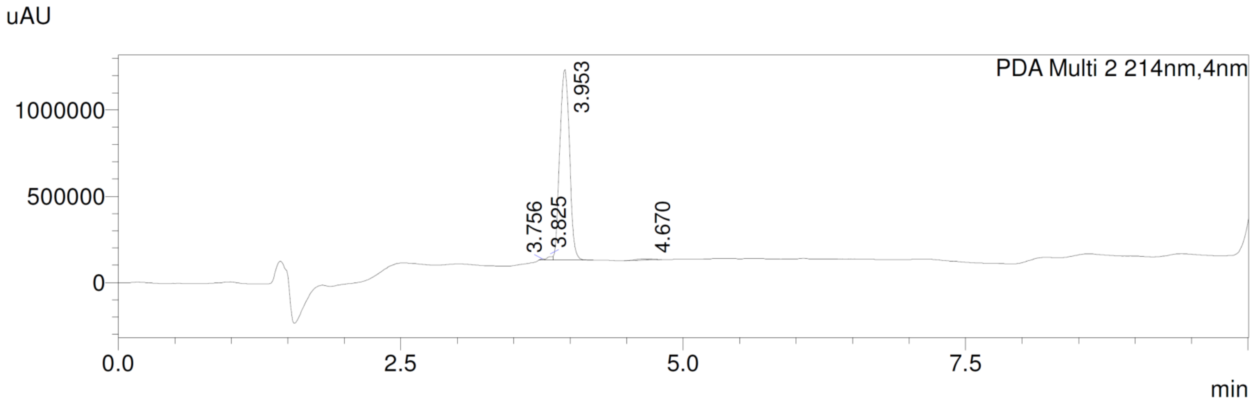

PDA Ch2 214nm

Peak#	Ret. Time	Area	Height	Area%	Conc.
1	3.756	21681	8351	0.318	0.318
2	3.825	78630	20815	1.153	1.153
3	3.953	6638793	1106908	97.358	97.358
4	4.670	79855	7381	1.171	1.171
Total		6818959	1143456	100.000	

2718.021 (R)-1-((4-(tert-butyl)cyclohexyl)methyl)-5-propylimidazolidin-2-imine. Compound 2718.021 was synthesized using the following reagents: (100mg) MBHA resin starting material, Boc-D-Norvaline-OH (R_4), and 4-tert-Butylcyclohexancecarboxylic acid (R_5). The final crude product was purified using HPLC as described above, with a gradient of (B) 0/2, 2/2, 20/27, 25/30, 27/98, 30/98. **Isolated Mass** 5.6mg, ^{1}**H NMR** (400 MHz, DMSO-d6) d ppm 10.20 (br. s., 1 H) 3.87 (d, J=8.80 Hz, 1 H) 3.55 - 3.75 (m, 2 H) 3.25 (dd, J=9.72, 5.69 Hz, 1 H) 2.95 (br. s., 1 H) 2.03 (br. s., 1 H) 1.62 (dt, J=10.09, 3.03 Hz, 2 H) 1.34 - 1.55 (m, 6 H) 1.25 (d, J=7.34 Hz, 2 H) 1.13 (br. s., 2 H) 0.87 - 1.00 (m, 5 H) 0.84 (s, 8 H) 0.83 (br. s., 1 H) **m/z** calcd $C_{17}H_{33}N_3$ $[M+H]^+$ 280.3, found 280.6 (MS ESI) **Purity** LC-MS: >98% (214 nm, peak area).

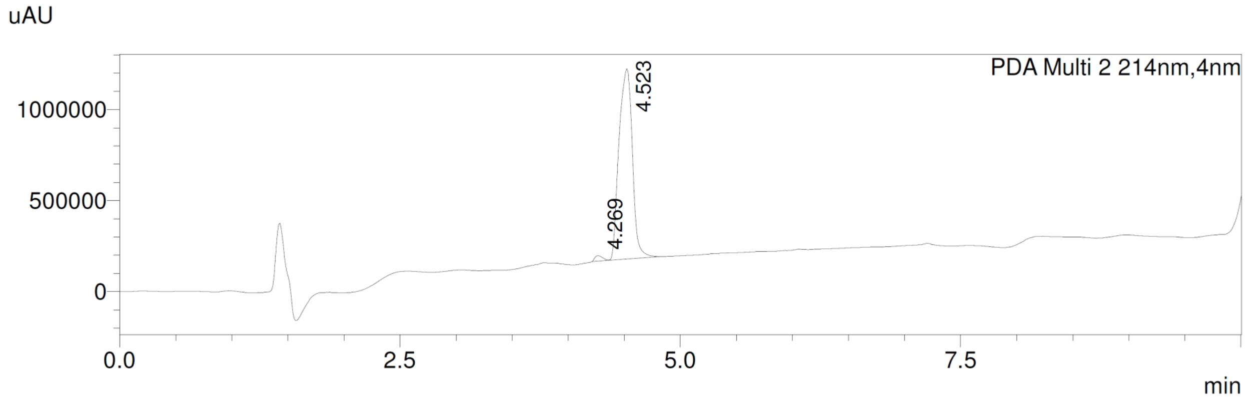

PDA Ch2 214nm

Peak#	Ret. Time	Area	Height	Area%	Conc.
1	4.269	145522	29146	1.651	1.651
2	4.523	8667181	1046010	98.349	98.349
Total		8812703	1075156	100.000	

2718.022 1-((4-(tert-butyl)cyclohexyl)methyl)-3-((R)-1-((S)-2-(((R)-2-imino-5-isobutylimidazolidin-1-yl)methyl)pyrrolidin-1-yl)-3-phenylpropan-2-yl)imidazolidin-2-imine. Compound 2718.022 was synthesized using the following reagents: (100mg) MBHA resin starting material, Boc-D-Leucine-OH (R_1), Boc-L-Proline-OH (R_2), Boc-D-Phenylalanine-OH (R_3), Boc-Glycine-OH (R_4), and 4-tert-Butylcyclohexancecarboxylic acid (R_5). The final crude product was purified using HPLC as described above, with a gradient of (B) 0/2, 2/2, 20/27, 25/30, 27/98, 30/98. **Isolated Mass** 6.8mg, **^{1}H NMR** (400 MHz, DMSO-d6) d ppm 8.13 (br. s., 1 H) 7.06 - 7.35 (m, 4 H) 4.31 - 4.41 (m, 1 H) 3.91 (q, J=8.15 Hz, 1 H) 3.65 (t, J=9.23 Hz, 2 H) 3.36 - 3.58 (m, 10 H) 3.15 - 3.36 (m, 5 H) 3.09 (dd, J=14.00, 7.76 Hz, 2 H) 2.97 (br. s., 1 H) 2.64 - 2.90 (m, 3 H) 2.20 - 2.48 (m, 2 H) 1.78 - 2.08 (m, 2 H) 1.64 - 1.78 (m, 2 H) 1.52 - 1.64 (m, 2 H) 1.32 - 1.52 (m, 5 H) 1.28 (br. s., 2 H) 0.86 - 1.12 (m, 7 H) 0.82 (s, 7 H) **m/z** calcd $C_{35}H_{59}N_7$ $[M+H]^+$ 578.5, found 578.85 (MS ESI) **Purity** LC-MS: >99% (214 nm, peak area).

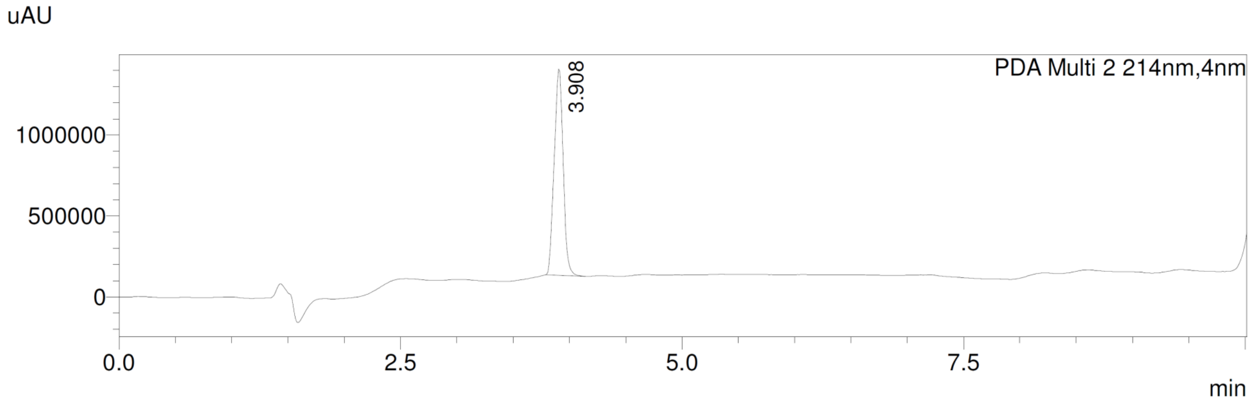

PDA Ch2 214nm

Peak#	Ret. Time	Area	Height	Area%	Conc.
1	3.908	7076967	1275564	100.000	100.000
Total		7076967	1275564	100.000	

2718.023 (S)-1-((S)-3-((4-(tert-butyl)cyclohexyl)methyl)-2-imino-1-(2-((S)-2-(((R)-2-imino-5-isobutylimidazolidin-1-yl)methyl)pyrrolidin-1-yl)ethyl)imidazolidin-4-yl)ethanol.

Compound 2718.023 was synthesized using the following reagents: (100mg) MBHA resin starting material, Boc-D-Leucine-OH (R_1), Boc-L-Proline-OH (R_2), Boc-Glycine-OH (R_3), Boc-D-Threonine(Bzl)-OH (R_4), and 4-tert-Butylcyclohexancecarboxylic acid (R_5). The final crude product was purified using HPLC as described above, with a gradient of (B) 0/2, 2/2, 20/26, 25/29, 27/98, 30/98. **Isolated Mass** 5.7mg, **^{1}H NMR** (400 MHz, DMSO-d6) d ppm 10.46 (br. s., 1 H) 8.47 (br. s., 2 H) 3.75 - 3.95 (m, 6 H) 3.64 - 3.70 (m, 2 H) 3.31 - 3.59 (m, 5 H) 2.99 - 3.24 (m, 4 H) 2.67 - 2.95 (m, 2 H) 2.30 (q, J=8.03 Hz, 2 H) 2.09 (d, J=12.84 Hz, 1 H) 1.67 - 1.89 (m, 3 H) 1.40 - 1.63 (m, 7 H) 1.32 - 1.38 (m, 1 H) 1.08 - 1.18 (m, 2 H) 0.84 (s, 8 H) 0.81 - 1.05 (m, 12 H) **m/z** calcd $C_{30}H_{57}N_7O$ $[M+H]^+$ 532.5, found 532.55 (MS ESI) **Purity** LC-MS: >99% (214 nm, peak area).

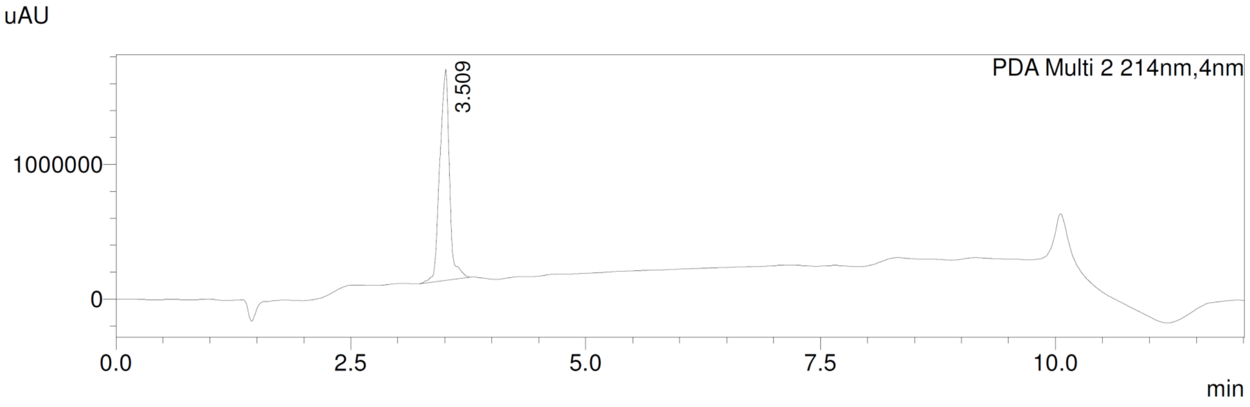

PDA Ch2 214nm

Peak#	Ret. Time	Area	Height	Area%	Conc.
1	3.509	11159595	1569912	100.000	100.000
Total		11159595	1569912	100.000	

2718.024 (S)-1-((S)-3-((4-(tert-butyl)cyclohexyl)methyl)-2-imino-1-((R)-1-((S)-2-((2-iminoimidazolidin-1-yl)methyl)pyrrolidin-1-yl)-3-phenylpropan-2-yl)imidazolidin-4-yl)ethanol. Compound 2718.024 was synthesized using the following reagents: (100mg) MBHA resin starting material, Boc-Glycine-OH (R_1), Boc-L-Proline-OH (R_2), Boc-D-Phenylalanine-OH (R_3), Boc-D-Threonine(Bzl)-OH (R_4), and 4-tert-Butylcyclohexancecarboxylic acid (R_5). The final crude product was purified using HPLC as described above, with a gradient of (B) 0/2, 2/2, 20/26, 25/29, 27/98, 30/98. **Isolated Mass** 6.4mg, **^{1}H NMR** (400 MHz, DMSO-d6) d ppm 7.97 (br. s., 1 H) 7.32 - 7.37 (m, 2 H) 7.12 - 7.31 (m, 3 H) 4.23 - 4.40 (m, 1 H) 3.74 - 4.00 (m, 2 H) 3.58 - 3.69 (m, 4 H) 3.37 - 3.56 (m, 6 H) 3.02 - 3.27 (m, 3 H) 3.00 (br. s., 1 H) 2.87 (d, J=4.03 Hz, 1 H) 2.74 (dd, J=13.88, 10.09 Hz, 2 H) 2.38 (d, J=8.80 Hz, 1 H) 2.08 - 2.32 (m, 1 H) 1.78 - 2.04 (m, 2 H) 1.57 - 1.76 (m, 2 H) 1.41 - 1.54 (m, 3 H) 1.38 (br. s., 1 H) 1.28 (d, J=11.74 Hz, 1 H) 1.01 (d, J=6.24 Hz, 4 H) 0.97 - 1.20 (m, 2 H) 0.82 (s, 9 H) 0.75 - 0.94 (m, 1 H) **m/z** calcd $C_{33}H_{55}N_7O$ $[M+H]^+$ 566.45, found 566.40 (MS ESI) **Purity** LC-MS: >84% (214 nm, peak area).

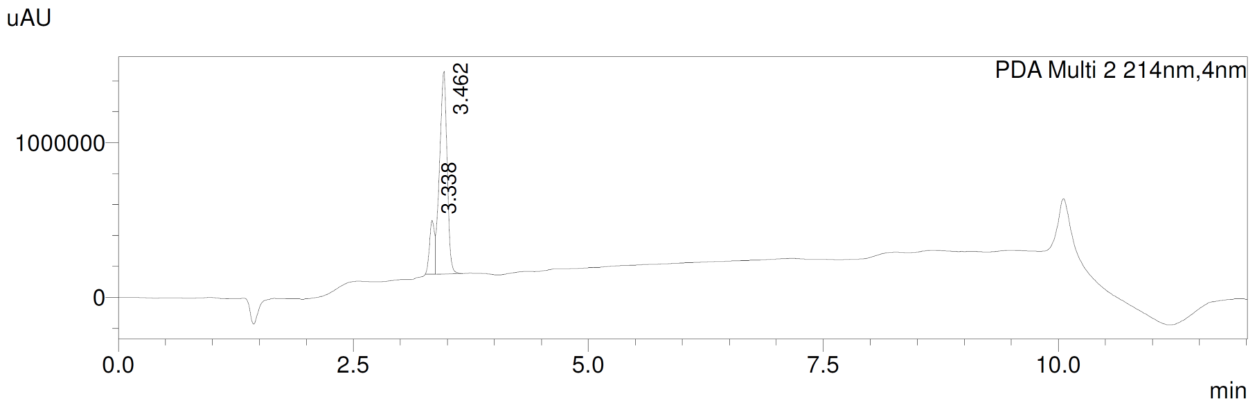

PDA Ch2 214nm

Peak#	Ret. Time	Area	Height	Area%	Conc.
1	3.338	1339002	349181	15.580	15.580
2	3.462	7255339	1311674	84.420	84.420
Total		8594340	1660855	100.000	

2718.025 1-((4-(tert-butyl)cyclohexyl)methyl)-3-((R)-1-cyclohexyl-3-((S)-2-(((R)-2-imino-5-isobutylimidazolidin-1-yl)methyl)pyrrolidin-1-yl)propan-2-yl)imidazolidin-2-imine.

Compound 2718.025 was synthesized using the following reagents: (100mg) MBHA resin starting material, Boc-D-Leucine-OH (R_1), Boc-L-Proline-OH (R_2), Boc-D-Cyclohexylalanine-OH (R_3), Boc-Glycine-OH (R_4), and 4-tert-Butylcyclohexancecarboxylic acid (R_5). The final crude product was purified using HPLC as described above, with a gradient of (B) 0/2, 2/2, 20/27, 25/30, 27/98, 30/98. **Isolated Mass** 5.9mg, **^{1}H NMR** (400 MHz, DMSO-d6) d ppm 10.30 (br. s., 1 H) 8.28 (br. s., 1 H) 4.02 (t, J=9.72 Hz, 1 H) 3.78 - 3.97 (m, 1 H) 3.38 - 3.67 (m, 11 H) 3.15 - 3.38 (m, 5 H) 2.98 - 3.15 (m, 1 H) 2.85 (br. s., 1 H) 2.78 (br. s., 1 H) 2.53 (br. s., 1 H) 2.20 - 2.37 (m, 2 H) 2.06 (br. s., 1 H) 1.76 - 1.98 (m, 2 H) 1.54 - 1.70 (m, 7 H) 1.39 - 1.53 (m, 5 H) 1.27 (br. s., 1 H) 1.06 - 1.23 (m, 5 H) 0.84 (s, 9 H) 0.73 - 1.02 (m, 7 H) **m/z** calcd $C_{36}H_{65}N_7$ $[M+H]^+$ 584.5, found 584.45 (MS ESI) **Purity** LC-MS: >99% (214 nm, peak area).

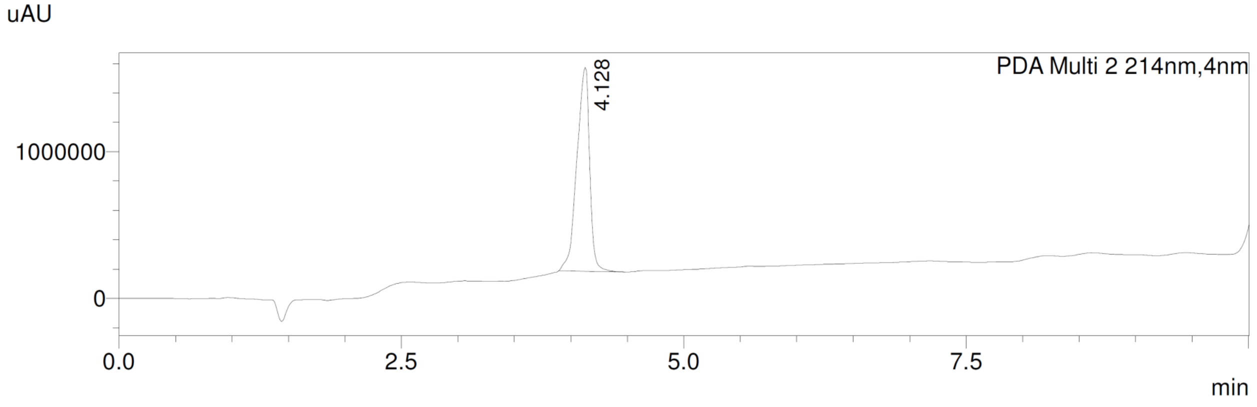

PDA Ch2 214nm

Peak#	Ret. Time	Area	Height	Area%	Conc.
1	4.128	10979245	1391568	100.000	100.000
Total		10979245	1391568	100.000	

2718.026 (S)-1-((S)-3-((4-(tert-butyl)cyclohexyl)methyl)-1-((R)-1-cyclohexyl-3-((S)-2-((2-iminoimidazolidin-1-yl)methyl)pyrrolidin-1-yl)propan-2-yl)-2-iminoimidazolidin-4-yl)ethanol. Compound 2718.026 was synthesized using the following reagents: (100mg) MBHA resin starting material, Boc-Glycine-OH (R_1), Boc-L-Proline-OH (R_2), Boc-D-Cyclohexylalanine-OH (R_3), Boc-D-Threonine(Bzl)-OH (R_4), and 4-tert-Butylcyclohexancecarboxylic acid (R_5). The final crude product was purified using HPLC as described above, with a gradient of (B) 0/2, 2/2, 20/26, 25/29, 27/98, 30/98. **Isolated Mass** 5.7mg, **^{1}H NMR** (400 MHz, DMSO-d6) d ppm 8.42 (br. s., 2 H) 8.27 (br. s., 2 H) 3.92 - 4.22 (m, 2 H) 3.57 - 3.87 (m, 9 H) 3.45 - 3.55 (m, 3 H) 3.18 - 3.40 (m, 3 H) 2.89 - 3.18 (m, 3 H) 2.60 - 2.83 (m, 1 H) 2.04 - 2.29 (m, 3 H) 1.77 - 2.04 (m, 2 H) 1.58 - 1.72 (m, 5 H) 1.40 - 1.57 (m, 5 H) 1.28 (d, J=3.79 Hz, 1 H) 1.19 (br. s., 1 H) 0.89 - 1.16 (m, 9 H) 0.84 (s, 8 H) **m/z** calcd $C_{33}H_{61}N_7O$ $[M+H]^+$ 572.5, found 572.55 (MS ESI) **Purity** LC-MS: >99% (214 nm, peak area).

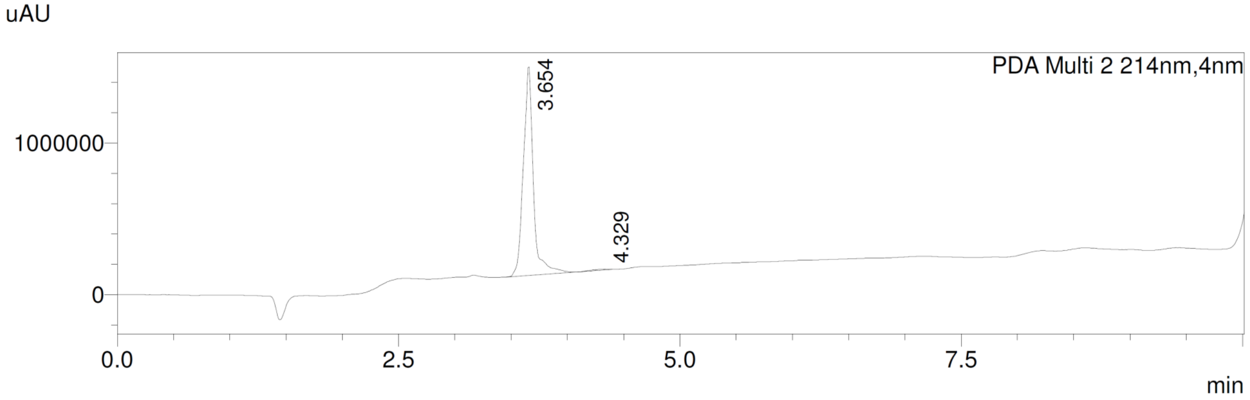

PDA Ch2 214nm

Peak#	Ret. Time	Area	Height	Area%	Conc.
1	3.654	8523568	1375667	99.134	99.134
2	4.329	74439	5115	0.866	0.866
Total		8598007	1380783	100.000	

2718.027 1-((4-(tert-butyl)cyclohexyl)methyl)-3-((R)-1-cyclohexyl-3-((S)-2-(((R)-2-imino-5-isopropylimidazolidin-1-yl)methyl)pyrrolidin-1-yl)propan-2-yl)imidazolidin-2-imine.

Compound 2718.027 was synthesized using the following reagents: (100mg) MBHA resin starting material, Boc-D-Valine-OH (R_1), Boc-L-Proline-OH (R_2), Boc-D-Cyclohexylalanine-OH (R_3), Boc-Glycine-OH (R_4), and 4-tert-Butylcyclohexancecarboxylic acid (R_5). The final crude product was purified using HPLC as described above, with a gradient of (B) 0/2, 2/2, 20/26, 25/29, 27/98, 30/98. **Isolated Mass** 6.8mg, ^{1}**H NMR** (400 MHz, DMSO-d6) d ppm 8.45 (br. s., 2 H) 3.75 - 4.05 (m, 2 H) 3.58 (d, J=8.31 Hz, 1 H) 3.26 - 3.55 (m, 19 H) 3.07 - 3.16 (m, 2 H) 2.77 - 2.98 (m, 2 H) 2.53 (br. s., 2 H) 2.21 - 2.35 (m, 2 H) 1.77 - 1.93 (m, 2 H) 1.59 - 1.77 (m, 5 H) 1.56 (br. s., 1 H) 1.33 - 1.53 (m, 5 H) 1.27 (br. s., 1 H) 1.05 - 1.23 (m, 5 H) 0.96 (t, J=11.49 Hz, 2 H) 0.84 (s, 8 H) 0.87 (s, 1 H) 0.76 (d, J=6.72 Hz, 2 H) **m/z** calcd $C_{34}H_{63}N_7$ $[M+H]^+$ 570.5, found 570.9 (MS ESI) **Purity** LC-MS: >99% (214 nm, peak area).

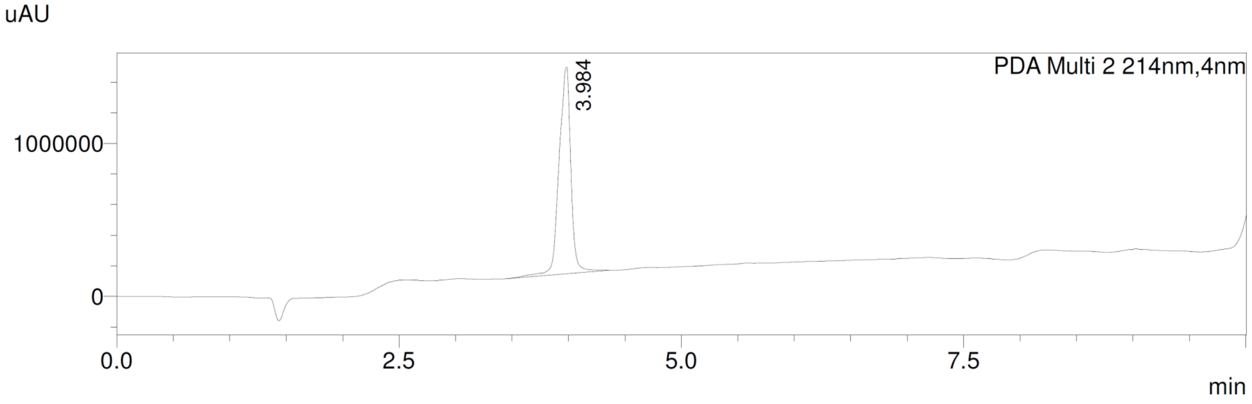

PDA Ch2 214nm

Peak#	Ret. Time	Area	Height	Area%	Conc.
1	3.984	9850854	1351317	100.000	100.000
Total		9850854	1351317	100.000	

2718.028 (R)-3-((4-(tert-butyl)cyclohexyl)methyl)-1-(2-((S)-2-(((R)-2-imino-5-isopropylimidazolidin-1-yl)methyl)pyrrolidin-1-yl)ethyl)-4-propylimidazolidin-2-imine. Compound 2718.028 was synthesized using the following reagents: (100mg) MBHA resin starting material, Boc-D-Valine-OH (R_1), Boc-L-Proline-OH (R_2), Boc-Glycine-OH (R_3), Boc-D-Norvaline-OH (R_4), and 4-tert-Butylcyclohexancecarboxylic acid (R_5). The final crude product was purified using HPLC as described above, with a gradient of (B) 0/2, 2/2, 20/26, 25/29, 27/98, 30/98. **Isolated Mass** 6.2mg, **^{1}H NMR** (400 MHz, DMSO-d6) d ppm 10.47 (br. s., 1 H) 8.57 (br. s., 1 H) 3.94 (ddd, J=9.75, 5.84, 3.61 Hz, 2 H) 3.61 - 3.88 (m, 6 H) 3.31 - 3.61 (m, 8 H) 3.12 (dt, J=9.54, 4.89 Hz, 1 H) 2.77 - 3.06 (m, 3 H) 2.24 - 2.41 (m, 1 H) 2.11 (td, J=6.82, 3.48 Hz, 1 H) 2.01 (br. s., 1 H) 1.68 - 1.91 (m, 3 H) 1.56 - 1.68 (m, 2 H) 1.35 - 1.56 (m, 5 H) 1.19 - 1.35 (m, 2 H) 1.03 - 1.19 (m, 2 H) 0.84 (s, 7 H) 0.79 - 1.03 (m, 7 H) 0.76 (d, J=6.72 Hz, 3 H) **m/z** calcd $C_{30}H_{57}N_7$ $[M+H]^+$ 516.5, found 516.55 (MS ESI) **Purity** LC-MS: >99% (214 nm, peak area).

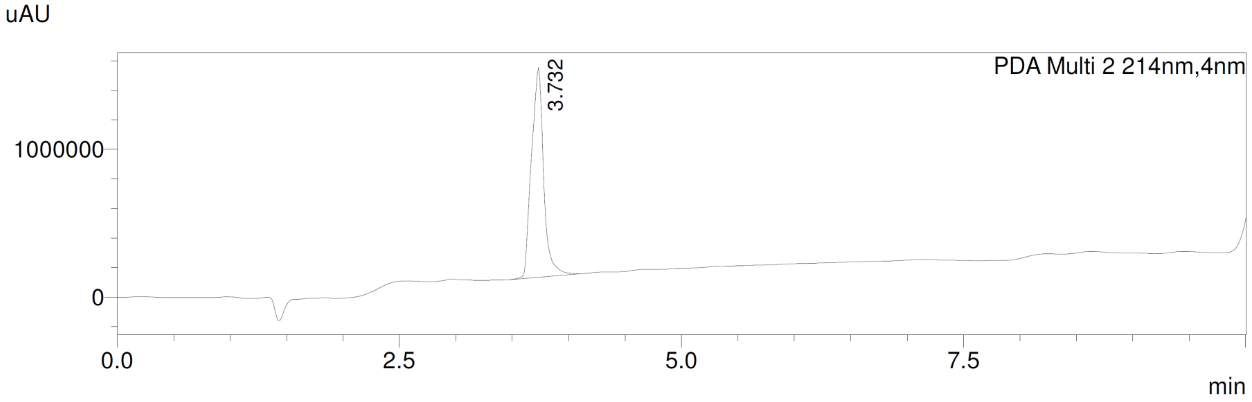

PDA Ch2 214nm

Peak#	Ret. Time	Area	Height	Area%	Conc.
1	3.732	10653792	1422252	100.000	100.000
Total		10653792	1422252	100.000	

2718.029 (R)-3-((4-(tert-butyl)cyclohexyl)methyl)-1-((R)-1-cyclohexyl-3-((S)-2-((2-iminoimidazolidin-1-yl)methyl)pyrrolidin-1-yl)propan-2-yl)-4-propylimidazolidin-2-imine. Compound 2718.029 was synthesized using the following reagents: (100mg) MBHA resin starting material, Boc-Glycine-OH (R_1), Boc-L-Proline-OH (R_2), Boc-D-Cyclohexylalanine-OH (R_3), Boc-D-Norvaline-OH (R_4), and 4-tert-Butylcyclohexancecarboxylic acid (R_5). The final crude product was purified using HPLC as described above, with a gradient of (B) 0/2, 2/2, 20/27, 25/30, 27/98, 30/98. **Isolated Mass** 6.1mg, **^{1}H NMR** (400 MHz, DMSO-d6) d ppm 8.29 (br. s., 1 H) 4.00 (br. s., 1 H) 3.70 - 3.93 (m, 3 H) 3.41 - 3.70 (m, 10 H) 3.16 - 3.41 (m, 4 H) 2.87 - 3.16 (m, 4 H) 2.63 - 2.84 (m, 1 H) 2.14 - 2.35 (m, 2 H) 2.07 (br. s., 1 H) 1.77 - 1.95 (m, 2 H) 1.64 - 1.77 (m, 4 H) 1.61 (br. s., 2 H) 1.35 - 1.55 (m, 6 H) 1.24 - 1.34 (m, 2 H) 1.05 - 1.20 (m, 4 H) 0.87 - 1.05 (m, 5 H) 0.84 (s, 8 H) **m/z** calcd $C_{34}H_{63}N_7$ $[M+H]^+$ 570.5, found 570.9 (MS ESI) **Purity** LC-MS: >99% (214 nm, peak area).

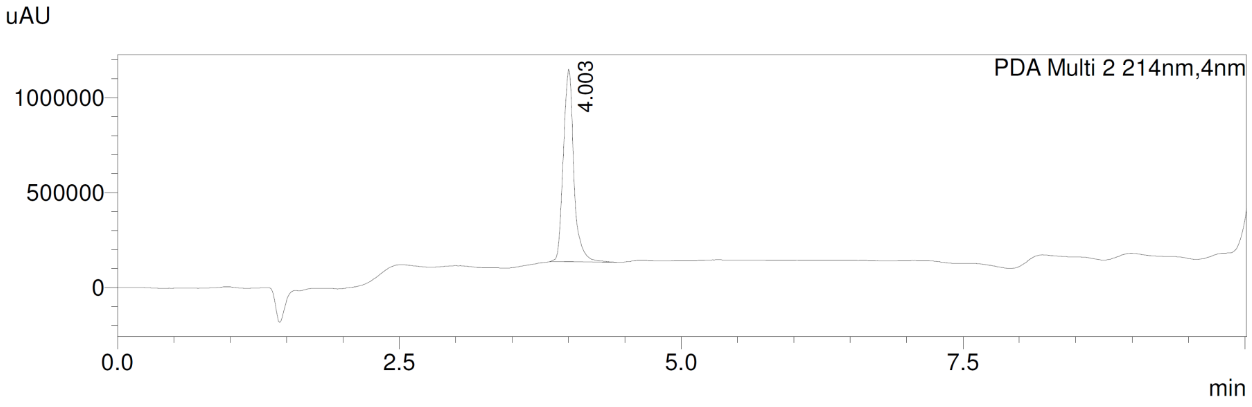

PDA Ch2 214nm

Peak#	Ret. Time	Area	Height	Area%	Conc.
1	4.003	6593125	1015319	100.000	100.000
Total		6593125	1015319	100.000	

2718.030 (R)-1-((R)-3-((4-(tert-butyl)cyclohexyl)methyl)-2-imino-1-((R)-1-((S)-2-(((R)-2-imino-5-isobutylimidazolidin-1-yl)methyl)pyrrolidin-1-yl)-3-phenylpropan-2-yl)imidazolidin-4-yl)ethanol. Compound 2718.030 was synthesized using the following reagents: (100mg) MBHA resin starting material, Boc-D-Leucine-OH (R_1), Boc-L-Proline-OH (R_2), Boc-D-Phenylalanine-OH (R_3), Boc-L-Threonine(Bzl)-OH (R_4), and 4-tert-Butylcyclohexancecarboxylic acid (R_5). The final crude product was purified using HPLC as described above, with a gradient of (B) 0/2, 2/2, 20/26, 25/29, 27/98, 30/98. **Isolated Mass** 6.1mg, **^{1}H NMR** (400 MHz, DMSO-d6) d ppm 10.58 (br. s., 1 H) 8.42 (br. s., 2 H) 8.21 (br. s., 1 H) 7.14 - 7.40 (m, 4 H) 4.16 - 4.46 (m, 2 H) 3.90 (dd, J=9.66, 5.38 Hz, 4 H) 3.48 - 3.78 (m, 5 H) 3.40 (dd, J=10.09, 4.34 Hz, 1 H) 3.14 - 3.35 (m, 3 H) 2.99 - 3.14 (m, 3 H) 2.92 (dd, J=14.31, 5.87 Hz, 1 H) 2.81 (dd, J=14.24, 9.11 Hz, 1 H) 2.67 (d, J=1.71 Hz, 1 H) 2.26 (d, J=9.05 Hz, 1 H) 2.16 (d, J=8.31 Hz, 1 H) 2.10 (br. s., 1 H) 1.73 - 1.90 (m, 1 H) 1.33 - 1.62 (m, 9 H) 1.09 (t, J=12.41 Hz, 1 H) 0.86 - 1.00 (m, 7 H) 0.82 (s, 8 H) 0.78 (d, J=6.36 Hz, 3 H) **m/z** calcd $C_{37}H_{63}N_7$ $[M+H]^+$ 622.5, found 622.9 (MS ESI) **Purity** LC-MS: >99% (214 nm, peak area).

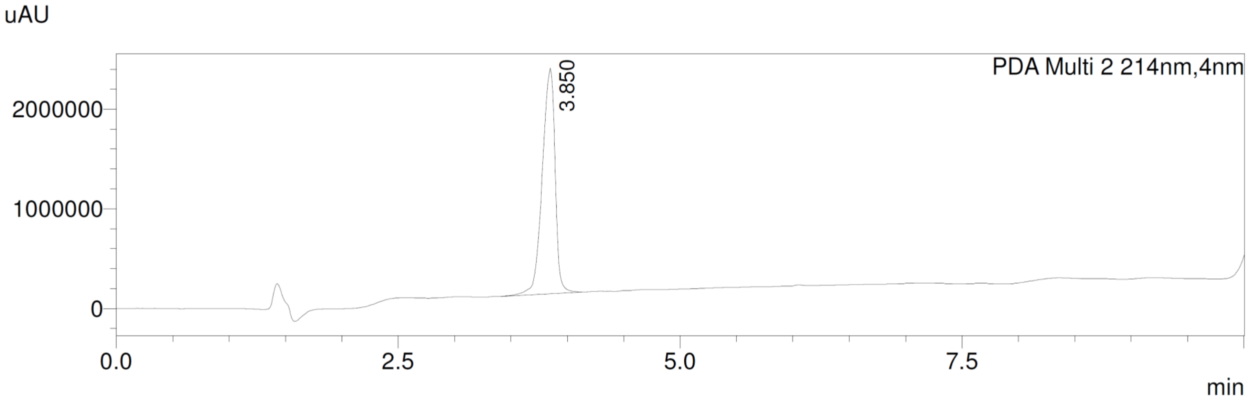

PDA Ch2 214nm

Peak#	Ret. Time	Area	Height	Area%	Conc.
1	3.850	17957846	2264806	100.000	100.000
Total		17957846	2264806	100.000	

2718.031 (S)-1-((S)-3-((4-(tert-butyl)cyclohexyl)methyl)-2-imino-1-((S)-1-((S)-2-(((R)-2-imino-5-isobutylimidazolidin-1-yl)methyl)pyrrolidin-1-yl)-3-phenylpropan-2-yl)imidazolidin-4-yl)ethanol. Compound 2718.031 was synthesized using the following reagents: (100mg) MBHA resin starting material, Boc-D-Leucine-OH (R_1), Boc-L-Proline-OH (R_2), Boc-L-Phenylalanine-OH (R_3), Boc-D-Threonine(Bzl)-OH (R_4), and 4-tert-Butylcyclohexancecarboxylic acid (R_5). The final crude product was purified using HPLC as described above, with a gradient of (B) 0/2, 2/2, 20/26, 25/29, 27/98, 30/98. **Isolated Mass** 6.1mg, **^{1}H NMR** (400 MHz, DMSO-d6) d ppm 8.45 (br. s., 2 H) 7.08 - 7.37 (m, 4 H) 4.26 - 4.50 (m, 1 H) 3.89 (d, J=8.44 Hz, 1 H) 3.72 (dd, J=9.84, 4.58 Hz, 1 H) 3.46 - 3.69 (m, 7 H) 3.27 - 3.46 (m, 4 H) 2.98 - 3.27 (m, 5 H) 2.72 - 2.94 (m, 3 H) 2.61 (dd, J=13.14, 6.79 Hz, 1 H) 2.44 (d, J=8.80 Hz, 1 H) 2.05 (br. s., 1 H) 1.76 - 1.95 (m, 1 H) 1.65 - 1.76 (m, 2 H) 1.37 - 1.65 (m, 7 H) 1.23 - 1.37 (m, 2 H) 0.98 - 1.23 (m, 2 H) 0.82 (s, 8 H) 0.74 - 0.98 (m, 7 H) 0.45 - 0.74 (m, 3 H) **m/z** calcd $C_{37}H_{63}N_7$ $[M+H]^+$ 622.5, found 622.9 (MS ESI) **Purity** LC-MS: >98% (214 nm, peak area).

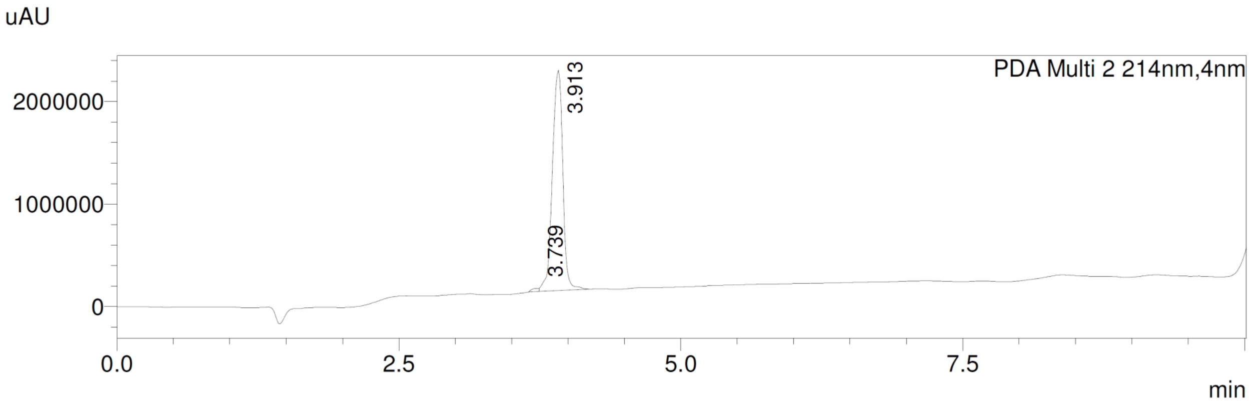

PDA Ch2 214nm

Peak#	Ret. Time	Area	Height	Area%	Conc.
1	3.739	145399	29507	1.044	1.044
2	3.913	13784971	2152242	98.956	98.956
Total		13930370	2181750	100.000	

2718.032 (S)-1-((S)-3-((4-(tert-butyl)cyclohexyl)methyl)-2-imino-1-((R)-1-((R)-2-(((R)-2-imino-5-isobutylimidazolidin-1-yl)methyl)pyrrolidin-1-yl)-3-phenylpropan-2-yl)imidazolidin-4-yl)ethanol. Compound 2718.032 was synthesized using the following reagents: (100mg) MBHA resin starting material, Boc-D-Leucine-OH (R_1), Boc-D-Proline-OH (R_2), Boc-D-Phenylalanine-OH (R_3), Boc-D-Threonine(Bzl)-OH (R_4), and 4-tert-Butylcyclohexancecarboxylic acid (R_5). The final crude product was purified using HPLC as described above, with a gradient of (B) 0/2, 2/2, 20/26, 25/29, 27/98, 30/98. **Isolated Mass** 6.1mg, **^{1}H NMR** (400 MHz, DMSO-d6) d ppm 8.44 (br. s., 2 H) 8.14 (br. s., 1 H) 7.15 - 7.36 (m, 4 H) 4.14 - 4.39 (m, 1 H) 3.87 - 4.14 (m, 2 H) 3.75 - 3.81 (m, 1 H) 3.43 - 3.73 (m, 9 H) 3.26 (dd, J=9.60, 6.42 Hz, 2 H) 2.93 - 3.21 (m, 5 H) 2.77 - 2.93 (m, 1 H) 2.70 (dd, J=14.55, 11.37 Hz, 1 H) 2.47 (br. s., 1 H) 2.24 - 2.43 (m, 1 H) 1.68 - 1.98 (m, 4 H) 1.49 - 1.65 (m, 3 H) 1.32 - 1.47 (m, 3 H) 1.26 (d, J=12.59 Hz, 2 H) 0.82 (s, 8 H) 0.76 - 1.04 (m, 10 H) **m/z** calcd $C_{37}H_{63}N_7O$ $[M+H]^+$ 622.5, found 622.55 (MS ESI) **Purity** LC-MS: >99% (214 nm, peak area).

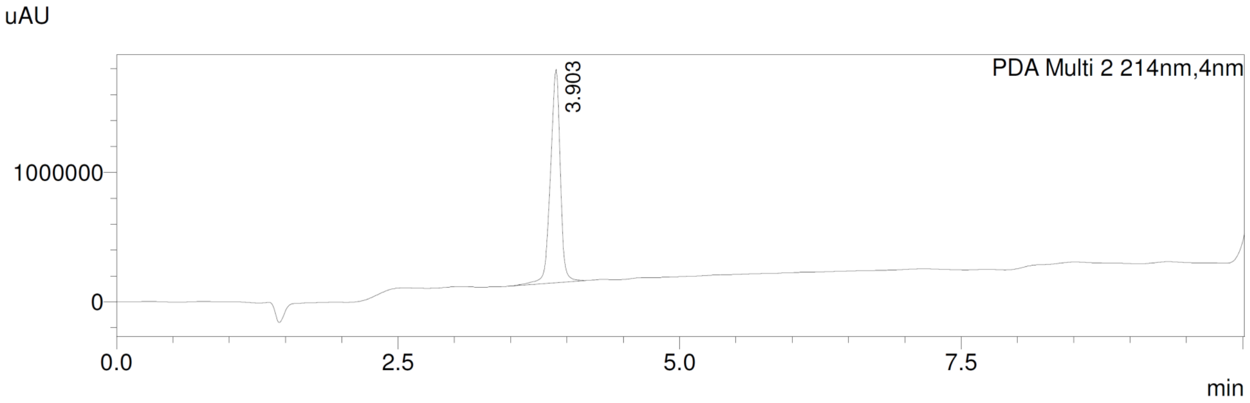

PDA Ch2 214nm

Peak#	Ret. Time	Area	Height	Area%	Conc.
1	3.903	10039379	1649962	100.000	100.000
Total		10039379	1649962	100.000	

2718.033 (S)-1-((S)-3-((4-(tert-butyl)cyclohexyl)methyl)-2-imino-1-((R)-1-((S)-2-(((S)-2-imino-5-isobutylimidazolidin-1-yl)methyl)pyrrolidin-1-yl)-3-phenylpropan-2-yl)imidazolidin-4-yl)ethanol. Compound 2718.033 was synthesized using the following reagents: (100mg) MBHA resin starting material, Boc-L-Leucine-OH (R_1), Boc-L-Proline-OH (R_2), Boc-D-Phenylalanine-OH (R_3), Boc-D-Threonine(Bzl)-OH (R_4), and 4-tert-Butylcyclohexancecarboxylic acid (R_5). The final crude product was purified using HPLC as described above, with a gradient of (B) 0/2, 2/2, 20/26, 25/29, 27/98, 30/98. **Isolated Mass** 5.8mg, **^{1}H NMR** (400 MHz, DMSO-d6) d ppm 8.46 (br. s., 1 H) 7.97 (br. s., 1 H) 7.35 (d, J=7.21 Hz, 2 H) 7.16 - 7.31 (m, 2 H) 4.34 (br. s., 1 H) 3.87 - 4.15 (m, 2 H) 3.76 - 3.87 (m, 2 H) 3.56 - 3.76 (m, 7 H) 3.36 - 3.56 (m, 5 H) 3.21 - 3.34 (m, 3 H) 2.85 - 3.15 (m, 4 H) 2.69 - 2.82 (m, 1 H) 2.63 (d, J=4.77 Hz, 1 H) 2.53 (br. s., 1 H) 2.35 (dd, J=12.35, 3.67 Hz, 1 H) 2.22 (d, J=8.93 Hz, 1 H) 1.91 - 2.12 (m, 1 H) 1.87 (br. s., 1 H) 1.70 (d, J=4.89 Hz, 1 H) 1.36 - 1.65 (m, 6 H) 1.29 (d, J=11.37 Hz, 1 H) 1.18 (br. s., 1 H) 1.01 (d, J=6.24 Hz, 3 H) 0.97 - 1.14 (m, 1 H) 0.82 (s, 7 H) 0.72 - 0.97 (m, 7 H) **m/z** calcd $C_{37}H_{63}N_7O$ $[M+H]^+$ 622.5, found 622.9 (MS ESI) **Purity** LC-MS: >97% (214 nm, peak area).

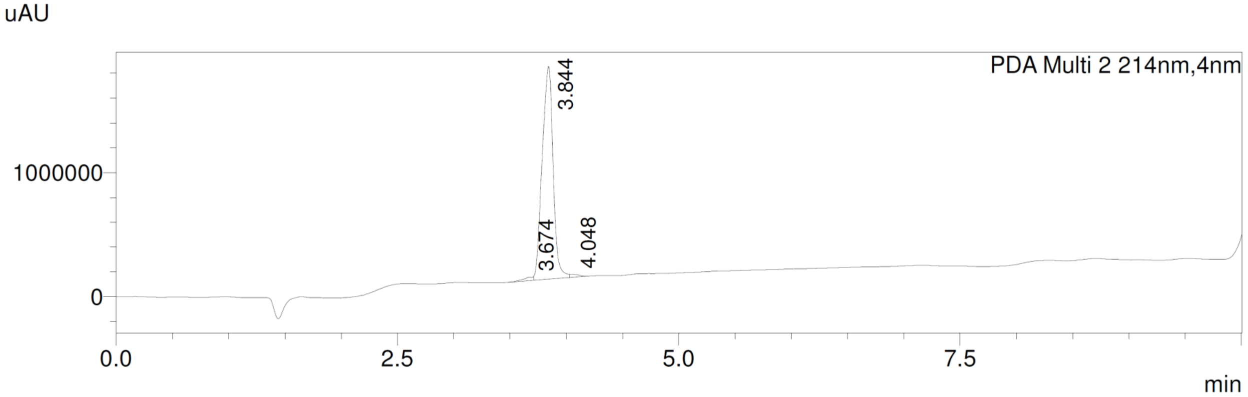

PDA Ch2 214nm

Peak#	Ret. Time	Area	Height	Area%	Conc.
1	3.674	190654	27134	1.604	1.604
2	3.844	11582394	1714248	97.423	97.423
3	4.048	115724	23497	0.973	0.973
Total		11888772	1764878	100.000	

2718.034 (R)-1-((R)-3-((4-(tert-butyl)cyclohexyl)methyl)-2-imino-1-((S)-1-((R)-2-(((S)-2-imino-5-isobutylimidazolidin-1-yl)methyl)pyrrolidin-1-yl)-3-phenylpropan-2-yl)imidazolidin-4-yl)ethanol. Compound 2718.034 was synthesized using the following reagents: (100mg) MBHA resin starting material, Boc-L-Leucine-OH (R_1), Boc-D-Proline-OH (R_2), Boc-L-Phenylalanine-OH (R_3), Boc-L-Threonine(Bzl)-OH (R_4), and 4-tert-Butylcyclohexancecarboxylic acid (R_5). The final crude product was purified using HPLC as described above, with a gradient of (B) 0/2, 2/2, 20/26, 25/29, 27/98, 30/98. **Isolated Mass** 5.8mg, **^{1}H NMR** (400 MHz, DMSO-d6) d ppm 8.44 (br. s., 2 H) 8.01 (br. s., 1 H) 7.15 - 7.38 (m, 4 H) 4.12 - 4.39 (m, 1 H) 3.77 - 4.06 (m, 3 H) 3.44 - 3.73 (m, 10 H) 3.27 - 3.44 (m, 4 H) 3.16 - 3.27 (m, 2 H) 2.97 - 3.14 (m, 3 H) 2.83 - 2.95 (m, 1 H) 2.77 (dd, J=14.00, 10.09 Hz, 2 H) 2.38 (dd, J=12.72, 3.67 Hz, 1 H) 2.10 - 2.32 (m, 1 H) 1.75 - 1.95 (m, 2 H) 1.53 - 1.75 (m, 3 H) 1.34 - 1.53 (m, 4 H) 1.29 (d, J=11.13 Hz, 1 H) 1.00 - 1.11 (m, 4 H) 0.82 (s, 8 H) 0.79 - 0.98 (m, 6 H) **m/z** calcd $C_{37}H_{63}N_7O$ $[M+H]^+$ 622.5, found 622.4 (MS ESI) **Purity** LC-MS: >97% (214 nm, peak area).

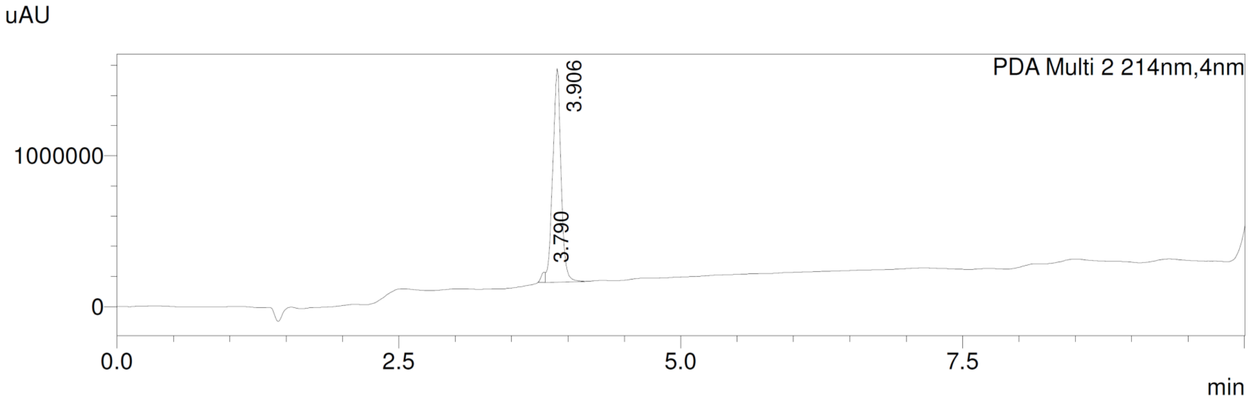

PDA Ch2 214nm

Peak#	Ret. Time	Area	Height	Area%	Conc.
1	3.790	175204	67794	2.365	2.365
2	3.906	7233056	1418164	97.635	97.635
Total		7408260	1485958	100.000	

2718.035 (R)-1-((R)-3-((4-(tert-butyl)cyclohexyl)methyl)-2-imino-1-((S)-1-((S)-2-(((R)-2-imino-5-isobutylimidazolidin-1-yl)methyl)pyrrolidin-1-yl)-3-phenylpropan-2-yl)imidazolidin-4-yl)ethanol. Compound 2718.035 was synthesized using the following reagents: (100mg) MBHA resin starting material, Boc-D-Leucine-OH (R_1), Boc-L-Proline-OH (R_2), Boc-L-Phenylalanine-OH (R_3), Boc-L-Threonine(Bzl)-OH (R_4), and 4-tert-Butylcyclohexancecarboxylic acid (R_5). The final crude product was purified using HPLC as described above, with a gradient of (B) 0/2, 2/2, 20/27, 25/30, 27/98, 30/98. **Isolated Mass** 5.8mg, **^{1}H NMR** (400 MHz, DMSO-d6) d ppm 8.17 (br. s., 1 H) 7.16 - 7.45 (m, 4 H) 4.32 (br. s., 1 H) 3.85 - 4.14 (m, 2 H) 3.73 - 3.85 (m, 2 H) 3.40 - 3.68 (m, 11 H) 3.38 (d, J=6.72 Hz, 1 H) 3.13 - 3.32 (m, 4 H) 3.02 - 3.11 (m, 2 H) 2.88 - 3.00 (m, 1 H) 2.84 (br. s., 1 H) 2.52 - 2.81 (m, 3 H) 2.19 - 2.48 (m, 1 H) 1.80 - 2.10 (m, 2 H) 1.69 - 1.80 (m, 2 H) 1.63 (d, J=8.56 Hz, 2 H) 1.32 - 1.54 (m, 4 H) 1.26 (d, J=11.00 Hz, 1 H) 1.13 (d, J=13.20 Hz, 1 H) 1.01 - 1.09 (m, 1 H) 0.71 - 1.01 (m, 16 H) **m/z** calcd $C_{37}H_{63}N_7O$ $[M+H]^+$ 622.5, found 622.6 (MS ESI) **Purity** LC-MS: >98% (214 nm, peak area).

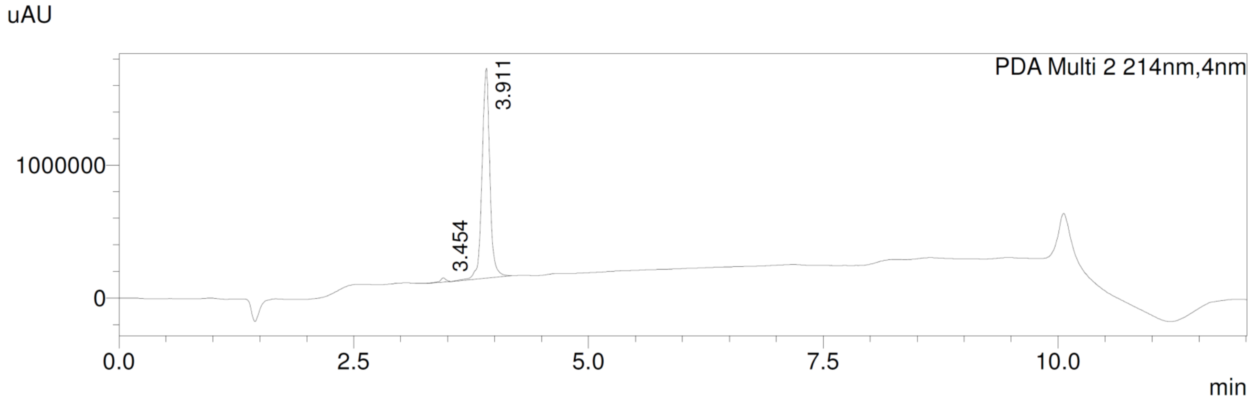

PDA Ch2 214nm

Peak#	Ret. Time	Area	Height	Area%	Conc.
1	3.454	130631	30357	1.402	1.402
2	3.911	9188223	1582183	98.598	98.598
Total		9318854	1612540	100.000	

2718.036 (R)-1-((R)-3-((4-(tert-butyl)cyclohexyl)methyl)-2-imino-1-((R)-1-((R)-2-(((R)-2-imino-5-isobutylimidazolidin-1-yl)methyl)pyrrolidin-1-yl)-3-phenylpropan-2-yl)imidazolidin-4-yl)ethanol. Compound 2718.036 was synthesized using the following reagents: (100mg) MBHA resin starting material, Boc-D-Leucine-OH (R_1), Boc-D-Proline-OH (R_2), Boc-D-Phenylalanine-OH (R_3), Boc-L-Threonine(Bzl)-OH (R_4), and 4-tert-Butylcyclohexancecarboxylic acid (R_5). The final crude product was purified using HPLC as described above, with a gradient of (B) 0/2, 2/2, 20/27, 25/30, 27/98, 30/98. **Isolated Mass** 7.0mg, **^{1}H NMR** (400 MHz, DMSO-d6) d ppm 8.42 (br. s., 1 H) 7.12 - 7.39 (m, 5 H) 4.30 - 4.56 (m, 1 H) 3.81 - 4.11 (m, 2 H) 3.43 - 3.73 (m, 8 H) 3.16 - 3.38 (m, 4 H) 2.90 - 3.16 (m, 5 H) 2.80 - 2.90 (m, 1 H) 2.74 (dd, J=14.92, 10.64 Hz, 1 H) 2.52 - 2.65 (m, 1 H) 2.19 - 2.48 (m, 1 H) 2.04 (br. s., 1 H) 1.90 (td, J=7.95, 4.16 Hz, 1 H) 1.64 - 1.80 (m, 2 H) 1.32 - 1.62 (m, 8 H) 0.98 - 1.25 (m, 2 H) 0.91 (dd, J=11.68, 5.93 Hz, 7 H) 0.82 (s, 9 H) 0.45 - 0.74 (m, 3 H) **m/z** calcd $C_{37}H_{63}N_7O$ $[M+H]^+$ 622.5, found 622.9 (MS ESI) **Purity** LC-MS: >99% (214 nm, peak area).

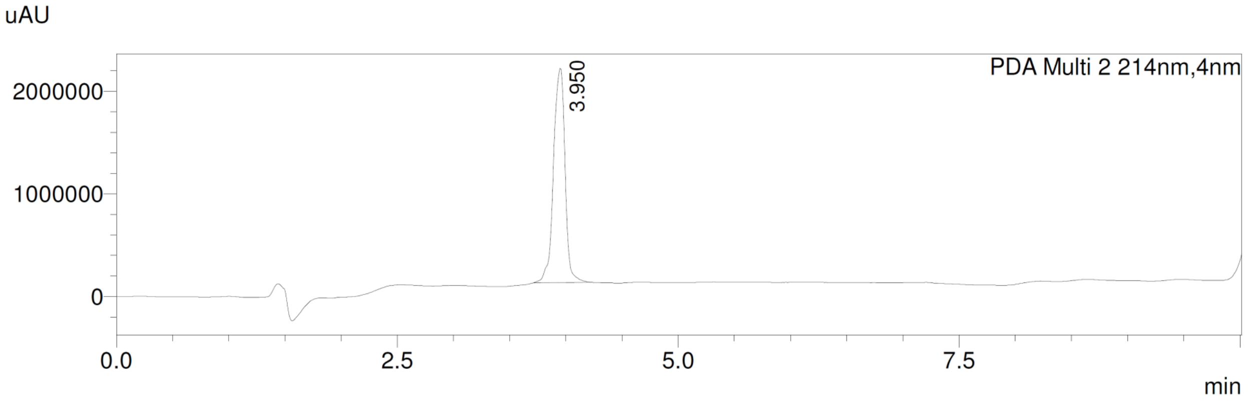

PDA Ch2 214nm

Peak#	Ret. Time	Area	Height	Area%	Conc.
1	3.950	14936669	2089721	100.000	100.000
Total		14936669	2089721	100.000	

2718.037 (R)-1-((R)-3-((4-(tert-butyl)cyclohexyl)methyl)-2-imino-1-((R)-1-((S)-2-(((S)-2-imino-5-isobutylimidazolidin-1-yl)methyl)pyrrolidin-1-yl)-3-phenylpropan-2-yl)imidazolidin-4-yl)ethanol. Compound 2718.037 was synthesized using the following reagents: (100mg) MBHA resin starting material, Boc-L-Leucine-OH (R_1), Boc-L-Proline-OH (R_2), Boc-D-Phenylalanine-OH (R_3), Boc-L-Threonine(Bzl)-OH (R_4), and 4-tert-Butylcyclohexancecarboxylic acid (R_5). The final crude product was purified using HPLC as described above, with a gradient of (B) 0/2, 2/2, 20/27, 25/30, 27/98, 30/98. **Isolated Mass** 6.5mg, **^{1}H NMR** (400 MHz, DMSO-d6) d ppm 8.20 (br. s., 1 H) 7.13 - 7.40 (m, 4 H) 4.44 (br. s., 1 H) 3.94 (d, J=8.68 Hz, 1 H) 3.79 (td, J=10.51, 5.26 Hz, 2 H) 3.42 - 3.72 (m, 9 H) 3.16 - 3.42 (m, 5 H) 2.98 - 3.16 (m, 2 H) 2.69 - 2.98 (m, 3 H) 2.64 (br. s., 2 H) 2.13 - 2.36 (m, 2 H) 2.11 (br. s., 1 H) 1.83 - 2.07 (m, 1 H) 1.65 - 1.83 (m, 2 H) 1.44 - 1.65 (m, 5 H) 1.27 - 1.44 (m, 3 H) 1.01 - 1.27 (m, 2 H) 0.85 - 1.01 (m, 7 H) 0.82 (s, 7 H) 0.74 (dd, J=13.45, 6.24 Hz, 3 H) **m/z** calcd $C_{37}H_{63}N_7O$ $[M+H]^+$ 622.5, found 622.9 (MS ESI) **Purity** LC-MS: >99% (214 nm, peak area).

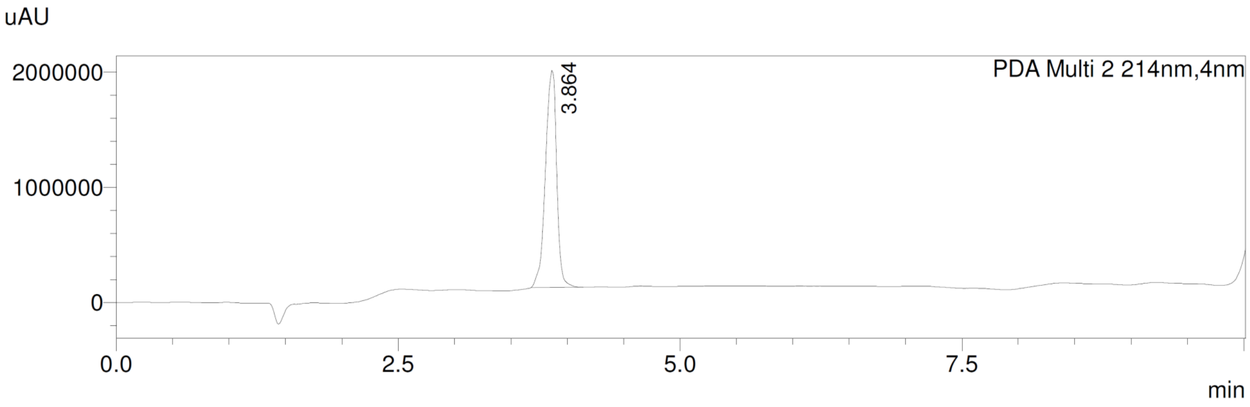

PDA Ch2 214nm

Peak#	Ret. Time	Area	Height	Area%	Conc.
1	3.864	13127579	1886421	100.000	100.000
Total		13127579	1886421	100.000	

2718.038 (S)-1-((S)-3-((4-(tert-butyl)cyclohexyl)methyl)-2-imino-1-((S)-1-((R)-2-(((R)-2-imino-5-isobutylimidazolidin-1-yl)methyl)pyrrolidin-1-yl)-3-phenylpropan-2-yl)imidazolidin-4-yl)ethanol. Compound 2718.038 was synthesized using the following reagents: (100mg) MBHA resin starting material, Boc-D-Leucine-OH (R_1), Boc-D-Proline-OH (R_2), Boc-L-Phenylalanine-OH (R_3), Boc-D-Threonine(Bzl)-OH (R_4), and 4-tert-Butylcyclohexancecarboxylic acid (R_5). The final crude product was purified using HPLC as described above, with a gradient of (B) 0/2, 2/2, 20/26, 25/29, 27/98, 30/98. **Isolated Mass** 6.9mg, **^{1}H NMR** (400 MHz, DMSO-d6) d ppm 8.46 (br. s., 1 H) 8.22 (br. s., 1 H) 7.09 - 7.38 (m, 4 H) 4.43 (br. s., 1 H) 3.93 (d, J=9.66 Hz, 1 H) 3.72 - 3.88 (m, 1 H) 3.45 - 3.72 (m, 6 H) 3.38 (br. s., 4 H) 3.16 - 3.35 (m, 6 H) 2.99 - 3.16 (m, 2 H) 2.85 - 2.99 (m, 1 H) 2.77 (dd, J=14.55, 9.05 Hz, 2 H) 2.52 - 2.71 (m, 1 H) 2.13 - 2.36 (m, 2 H) 2.10 (br. s., 1 H) 2.00 (dd, J=13.20, 6.24 Hz, 1 H) 1.64 - 1.86 (m, 2 H) 1.34 - 1.64 (m, 8 H) 0.98 - 1.27 (m, 2 H) 0.85 - 0.98 (m, 7 H) 0.82 (s, 7 H) 0.62 - 0.80 (m, 3 H) **m/z** calcd $C_{37}H_{63}N_7O$ $[M+H]^+$ 622.5, found 622.85 (MS ESI) **Purity** LC-MS: >98% (214 nm, peak area).

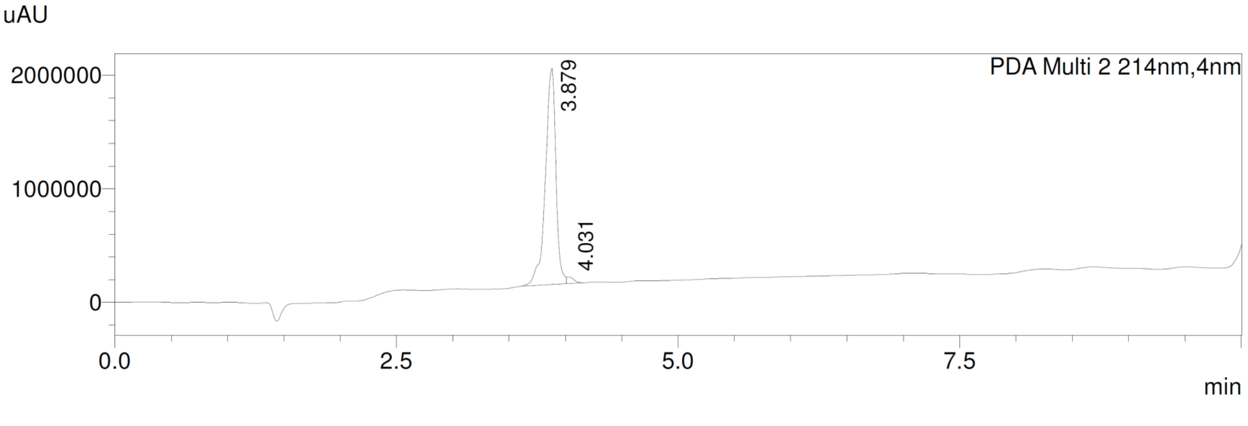

PDA Ch2 214nm

Peak#	Ret. Time	Area	Height	Area%	Conc.
1	3.879	12400151	1907510	98.160	98.160
2	4.031	232439	56993	1.840	1.840
Total		12632590	1964503	100.000	

2718.039 (S)-1-((S)-3-((4-(tert-butyl)cyclohexyl)methyl)-2-imino-1-((S)-1-((S)-2-(((S)-2-imino-5-isobutylimidazolidin-1-yl)methyl)pyrrolidin-1-yl)-3-phenylpropan-2-yl)imidazolidin-4-yl)ethanol. Compound 2718.039 was synthesized using the following reagents: (100mg) MBHA resin starting material, Boc-L-Leucine-OH (R_1), Boc-L-Proline-OH (R_2), Boc-L-Phenylalanine-OH (R_3), Boc-D-Threonine(Bzl)-OH (R_4), and 4-tert-Butylcyclohexancecarboxylic acid (R_5). The final crude product was purified using HPLC as described above, with a gradient of (B) 0/2, 2/2, 20/26, 25/29, 27/98, 30/98. **Isolated Mass** 6.1mg, **^{1}H NMR** (400 MHz, DMSO-d6) d ppm 10.58 (br. s., 1 H) 8.45 (br. s., 2 H) 7.13 - 7.38 (m, 4 H) 4.46 (br. s., 1 H) 3.84 - 4.13 (m, 3 H) 3.44 - 3.74 (m, 11 H) 3.16 - 3.39 (m, 3 H) 2.90 - 3.16 (m, 5 H) 2.80 - 2.90 (m, 1 H) 2.74 (dd, J=14.92, 10.76 Hz, 1 H) 2.57 (d, J=5.99 Hz, 1 H) 2.37 - 2.48 (m, 1 H) 2.04 (br. s., 1 H) 1.80 - 1.98 (m, 1 H) 1.65 - 1.80 (m, 2 H) 1.32 - 1.62 (m, 8 H) 0.99 - 1.23 (m, 2 H) 0.82 (s, 8 H) 0.74 - 0.99 (m, 6 H) 0.64 (d, J=6.24 Hz, 2 H) **m/z** calcd $C_{37}H_{63}N_7O$ $[M+H]^+$ 622.5, found 622.55 (MS ESI) **Purity** LC-MS: >99% (214 nm, peak area).

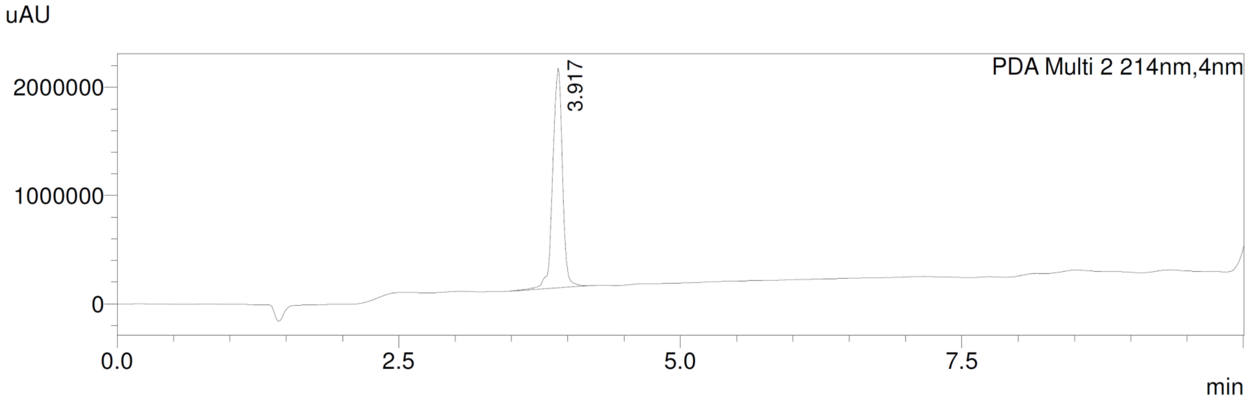

PDA Ch2 214nm

Peak#	Ret. Time	Area	Height	Area%	Conc.
1	3.917	12276638	2030768	100.000	100.000
Total		12276638	2030768	100.000	

2718.040 (S)-1-((S)-3-((4-(tert-butyl)cyclohexyl)methyl)-2-imino-1-((R)-1-((R)-2-(((S)-2-imino-5-isobutylimidazolidin-1-yl)methyl)pyrrolidin-1-yl)-3-phenylpropan-2-yl)imidazolidin-4-yl)ethanol. Compound 2718.040 was synthesized using the following reagents: (100mg) MBHA resin starting material, Boc-L-Leucine-OH (R_1), Boc-D-Proline-OH (R_2), Boc-D-Phenylalanine-OH (R_3), Boc-D-Threonine(Bzl)-OH (R_4), and 4-tert-Butylcyclohexancecarboxylic acid (R_5). The final crude product was purified using HPLC as described above, with a gradient of (B) 0/2, 2/2, 20/27, 25/30, 27/98, 30/98. **Isolated Mass** 6.9mg, **^{1}H NMR** (400 MHz, DMSO-d6) d ppm 8.25 (br. s., 1 H) 7.10 - 7.38 (m, 5 H) 4.36 (br. s., 1 H) 3.86 - 4.15 (m, 2 H) 3.79 (dt, J=11.95, 5.95 Hz, 1 H) 3.48 - 3.73 (m, 6 H) 3.33 - 3.48 (m, 3 H) 3.13 - 3.33 (m, 4 H) 2.91 - 3.12 (m, 3 H) 2.84 (br. s., 1 H) 2.75 (dd, J=14.31, 11.00 Hz, 1 H) 2.19 - 2.48 (m, 1 H) 1.86 (dt, J=11.52, 7.99 Hz, 2 H) 1.69 - 1.80 (m, 2 H) 1.54 - 1.69 (m, 2 H) 1.32 - 1.54 (m, 5 H) 1.26 (d, J=10.51 Hz, 1 H) 1.05 - 1.19 (m, 1 H) 0.82 (s, 8 H) 0.75 - 1.05 (m, 12 H) **m/z** calcd $C_{37}H_{63}N_7O$ $[M+H]^+$ 622.5, found 622.85 (MS ESI) **Purity** LC-MS: >99% (214 nm, peak area).

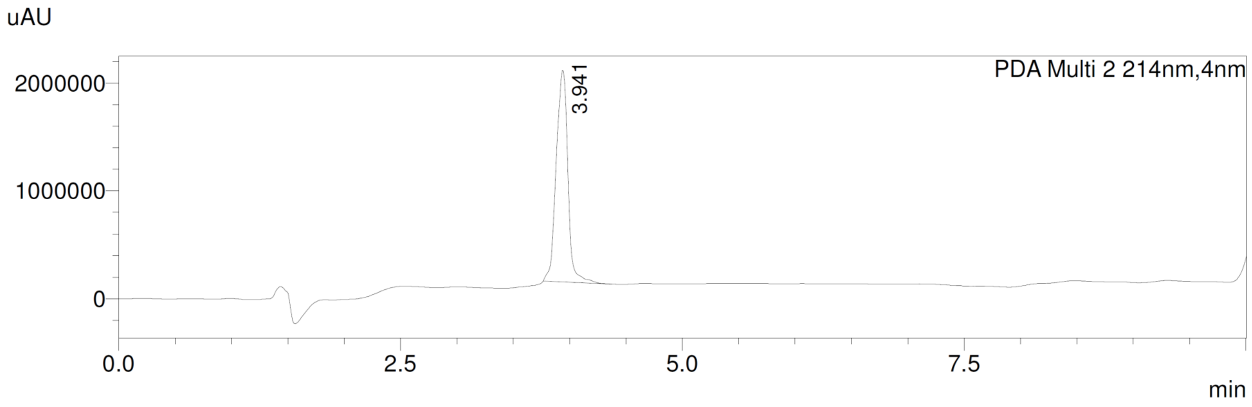

PDA Ch2 214nm

Peak#	Ret. Time	Area	Height	Area%	Conc.
1	3.941	14098173	1962609	100.000	100.000
Total		14098173	1962609	100.000	

2718.041 (R)-1-((R)-3-((4-(tert-butyl)cyclohexyl)methyl)-2-imino-1-((S)-1-((R)-2-(((R)-2-imino-5-isobutylimidazolidin-1-yl)methyl)pyrrolidin-1-yl)-3-phenylpropan-2-yl)imidazolidin-4-yl)ethanol. Compound 2718.041 was synthesized using the following reagents: (100mg) MBHA resin starting material, Boc-D-Leucine-OH (R_1), Boc-D-Proline-OH (R_2), Boc-L-Phenylalanine-OH (R_3), Boc-L-Threonine(Bzl)-OH (R_4), and 4-tert-Butylcyclohexancecarboxylic acid (R_5). The final crude product was purified using HPLC as described above, with a gradient of (B) 0/2, 2/2, 20/27, 25/30, 27/98, 30/98. **Isolated Mass** 5.3mg, **^{1}H NMR** (400 MHz, DMSO-d6) d ppm 7.92 (br. s., 1 H) 7.35 (d, J=7.09 Hz, 1 H) 7.12 - 7.32 (m, 2 H) 4.14 - 4.44 (m, 1 H) 3.88 - 4.14 (m, 1 H) 3.56 - 3.86 (m, 6 H) 3.38 - 3.56 (m, 5 H) 3.13 - 3.38 (m, 5 H) 2.83 - 3.13 (m, 3 H) 2.75 (dd, J=14.00, 10.45 Hz, 1 H) 2.63 (d, J=4.52 Hz, 1 H) 2.30 - 2.48 (m, 1 H) 2.23 (q, J=8.40 Hz, 1 H) 1.91 - 2.11 (m, 1 H) 1.87 (br. s., 1 H) 1.50 - 1.80 (m, 3 H) 1.33 - 1.50 (m, 3 H) 1.29 (d, J=11.25 Hz, 1 H) 1.14 - 1.24 (m, 1 H) 1.04 (d, J=6.11 Hz, 12 H) 0.97 - 1.14 (m, 1 H) 0.91 (dd, J=10.70, 6.05 Hz, 5 H) 0.82 (s, 7 H) **m/z** calcd $C_{37}H_{63}N_7O$ $[M+H]^+$ 622.5, found 622.6 (MS ESI) **Purity** LC-MS: >96% (214 nm, peak area).

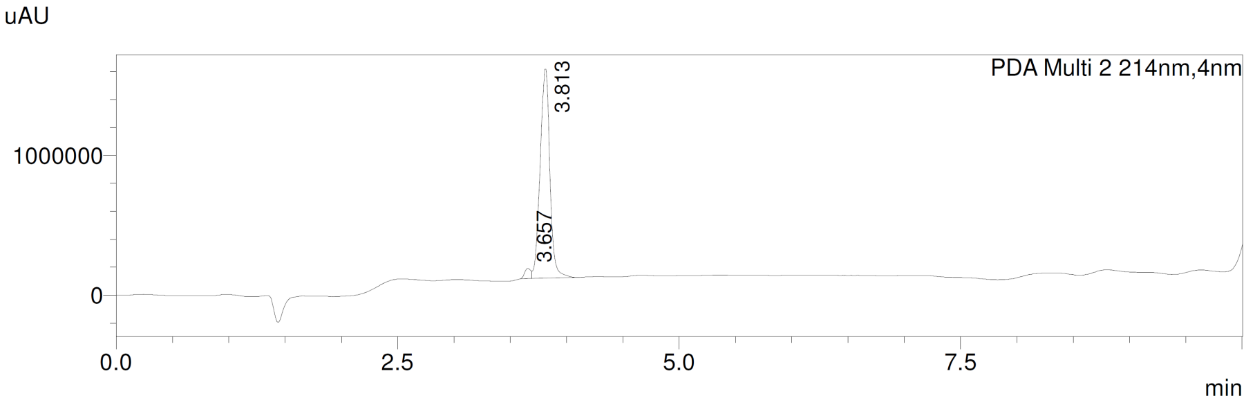

PDA Ch2 214nm

Peak#	Ret. Time	Area	Height	Area%	Conc.
1	3.657	279213	71788	3.012	3.012
2	3.813	8991569	1494188	96.988	96.988
Total		9270782	1565976	100.000	

2718.042 (R)-1-((R)-3-((4-(tert-butyl)cyclohexyl)methyl)-2-imino-1-((S)-1-((S)-2-(((S)-2-imino-5-isobutylimidazolidin-1-yl)methyl)pyrrolidin-1-yl)-3-phenylpropan-2-yl)imidazolidin-4-yl)ethanol. Compound 2718.042 was synthesized using the following reagents: (100mg) MBHA resin starting material, Boc-L-Leucine-OH (R_1), Boc-L-Proline-OH (R_2), Boc-L-Phenylalanine-OH (R_3), Boc-L-Threonine(Bzl)-OH (R_4), and 4-tert-Butylcyclohexancecarboxylic acid (R_5). The final crude product was purified using HPLC as described above, with a gradient of (B) 0/2, 2/2, 20/27, 25/30, 27/98, 30/98. **Isolated Mass** 6.4mg, **^{1}H NMR** (400 MHz, DMSO-d6) d ppm 8.15 (br. s., 1 H) 7.09 - 7.37 (m, 4 H) 4.36 (br. s., 1 H) 3.88 - 4.18 (m, 2 H) 3.73 - 3.81 (m, 2 H) 3.62 - 3.72 (m, 3 H) 3.46 - 3.60 (m, 4 H) 2.99 - 3.29 (m, 6 H) 2.77 - 2.90 (m, 1 H) 2.52 - 2.77 (m, 1 H) 2.19 - 2.48 (m, 2 H) 1.81 - 2.04 (m, 1 H) 1.69 - 1.81 (m, 2 H) 1.58 - 1.69 (m, 2 H) 1.52 (dd, J=12.47, 6.11 Hz, 1 H) 1.31 - 1.47 (m, 3 H) 1.25 (d, J=11.74 Hz, 1 H) 0.79 - 1.07 (m, 24 H) **m/z** calcd $C_{37}H_{63}N_7O$ $[M+H]^+$ 622.5, found 622.9 (MS ESI) **Purity** LC-MS: >99% (214 nm, peak area).

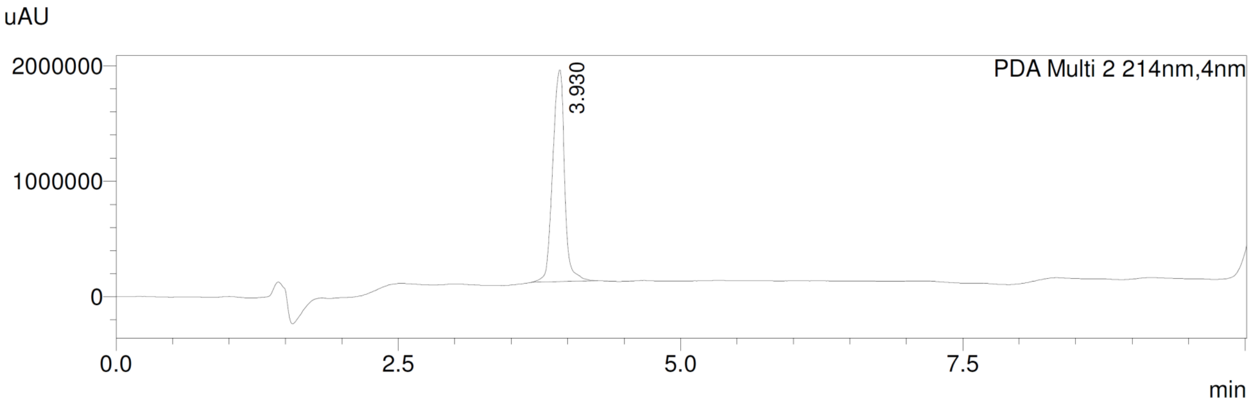

PDA Ch2 214nm

Peak#	Ret. Time	Area	Height	Area%	Conc.
1	3.930	13397855	1833665	100.000	100.000
Total		13397855	1833665	100.000	

2718.043 (R)-1-((R)-3-((4-(tert-butyl)cyclohexyl)methyl)-2-imino-1-((R)-1-((R)-2-(((S)-2-imino-5-isobutylimidazolidin-1-yl)methyl)pyrrolidin-1-yl)-3-phenylpropan-2-yl)imidazolidin-4-yl)ethanol. Compound 2718.043 was synthesized using the following reagents: (100mg) MBHA resin starting material, Boc-L-Leucine-OH (R_1), Boc-D-Proline-OH (R_2), Boc-D-Phenylalanine-OH (R_3), Boc-L-Threonine(Bzl)-OH (R_4), and 4-tert-Butylcyclohexancecarboxylic acid (R_5). The final crude product was purified using HPLC as described above, with a gradient of (B) 0/2, 2/2, 20/27, 25/30, 27/98, 30/98. **Isolated Mass** 6.6mg, **^{1}H NMR** (400 MHz, DMSO-d6) d ppm 8.44 (br. s., 1 H) 7.10 - 7.40 (m, 5 H) 4.35 - 4.51 (m, 1 H) 3.81 - 4.10 (m, 2 H) 3.73 (dd, J=10.64, 5.26 Hz, 1 H) 3.46 - 3.69 (m, 7 H) 3.27 - 3.46 (m, 4 H) 2.98 - 3.27 (m, 5 H) 2.71 - 2.95 (m, 3 H) 2.61 (d, J=6.36 Hz, 1 H) 2.45 (d, J=8.68 Hz, 1 H) 2.06 (d, J=7.70 Hz, 1 H) 1.66 - 1.93 (m, 3 H) 1.38 - 1.60 (m, 6 H) 1.30 - 1.36 (m, 1 H) 1.00 - 1.17 (m, 2 H) 0.82 (s, 8 H) 0.75 - 0.99 (m, 7 H) 0.45 - 0.75 (m, 3 H) **m/z** calcd $C_{37}H_{63}N_7O$ $[M+H]^+$ 622.5, found 622.85 (MS ESI) **Purity** LC-MS: >99% (214 nm, peak area).

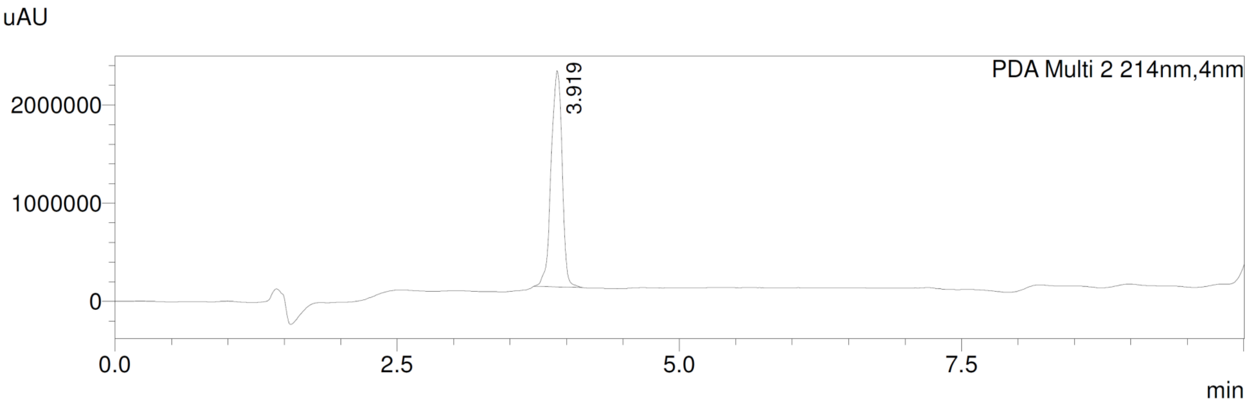

PDA Ch2 214nm

Peak#	Ret. Time	Area	Height	Area%	Conc.
1	3.919	14916863	2203116	100.000	100.000
Total		14916863	2203116	100.000	

2718.044 (S)-1-((S)-3-((4-(tert-butyl)cyclohexyl)methyl)-2-imino-1-((S)-1-((R)-2-(((S)-2-imino-5-isobutylimidazolidin-1-yl)methyl)pyrrolidin-1-yl)-3-phenylpropan-2-yl)imidazolidin-4-yl)ethanol. Compound 2718.044 was synthesized using the following reagents: (100mg) MBHA resin starting material, Boc-L-Leucine-OH (R_1), Boc-D-Proline-OH (R_2), Boc-L-Phenylalanine-OH (R_3), Boc-D-Threonine(Bzl)-OH (R_4), and 4-tert-Butylcyclohexancecarboxylic acid (R_5). The final crude product was purified using HPLC as described above, with a gradient of (B) 0/2, 2/2, 20/26, 25/29, 27/98, 30/98. **Isolated Mass** 6.2mg, **^{1}H NMR** (400 MHz, DMSO-d6) d ppm 10.54 (br. s., 1 H) 8.41 (br. s., 2 H) 8.21 (br. s., 1 H) 7.13 - 7.39 (m, 4 H) 4.15 - 4.45 (m, 4 H) 3.89 (dd, J=9.96, 5.81 Hz, 2 H) 3.48 - 3.77 (m, 4 H) 3.40 (dd, J=10.09, 4.34 Hz, 1 H) 3.14 - 3.35 (m, 3 H) 2.99 - 3.14 (m, 3 H) 2.92 (dd, J=14.37, 5.93 Hz, 1 H) 2.81 (dd, J=14.18, 9.05 Hz, 1 H) 2.61 - 2.74 (m, 1 H) 2.05 - 2.31 (m, 2 H) 1.75 - 1.90 (m, 1 H) 1.33 - 1.62 (m, 9 H) 1.00 - 1.25 (m, 2 H) 0.86 - 1.00 (m, 6 H) 0.82 (s, 8 H) 0.78 (d, J=6.36 Hz, 3 H) **m/z** calcd $C_{37}H_{63}N_7O$ $[M+H]^+$ 622.5, found 622.4 (MS ESI) **Purity** LC-MS: >99% (214 nm, peak area).

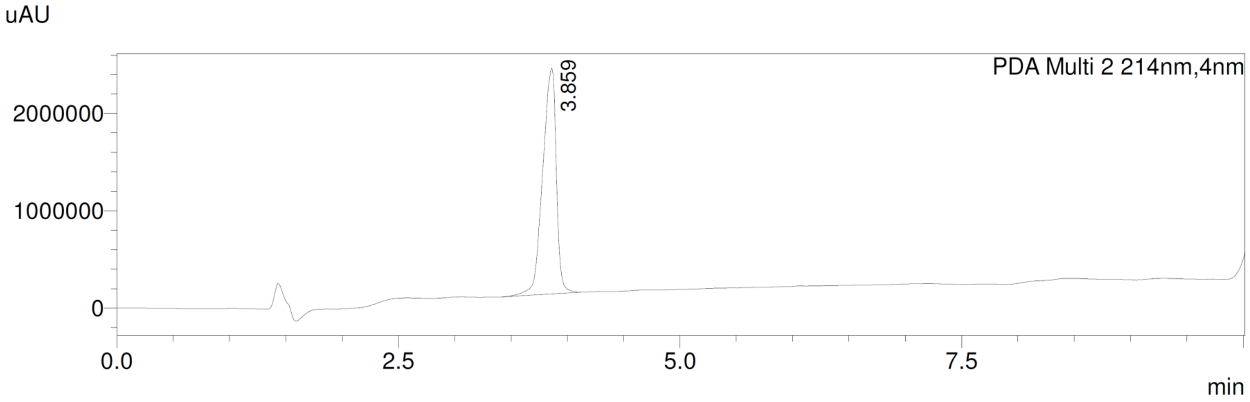

PDA Ch2 214nm

Peak#	Ret. Time	Area	Height	Area%	Conc.
1	3.859	19583202	2324147	100.000	100.000
Total		19583202	2324147	100.000	

2718.045 (S)-1-((S)-3-((4-(tert-butyl)cyclohexyl)methyl)-1-((R)-1-cyclohexyl-3-((S)-2-(((R)-2-imino-5-isobutylimidazolidin-1-yl)methyl)pyrrolidin-1-yl)propan-2-yl)-2-iminoimidazolidin-4-yl)ethanol. Compound 2718.045 was synthesized using the following reagents: (100mg) MBHA resin starting material, Boc-D-Leucine-OH (R_1), Boc-L-Proline-OH (R_2), Boc-D-Cyclohexylalanine-OH (R_3), Boc-L-Threonine(Bzl)-OH (R_4), and 4-tert-Butylcyclohexancecarboxylic acid (R_5). The final crude product was purified using HPLC as described above, with a gradient of (B) 0/2, 2/2, 20/27, 25/30, 27/98, 30/98. **Isolated Mass** 7.2mg, **^{1}H NMR** (400 MHz, DMSO-d6) d ppm 8.22 (br. s., 1 H) 3.99 (dd, J=8.99, 4.58 Hz, 2 H) 3.82 - 3.94 (m, 2 H) 3.58 - 3.82 (m, 2 H) 3.42 - 3.58 (m, 4 H) 3.19 - 3.42 (m, 10 H) 3.11 - 3.18 (m, 1 H) 3.06 - 3.09 (m, 1 H) 2.93 (t, J=11.74 Hz, 1 H) 2.52 - 2.76 (m, 1 H) 2.09 - 2.28 (m, 2 H) 1.74 - 1.96 (m, 2 H) 1.55 - 1.71 (m, 8 H) 1.51 (d, J=12.35 Hz, 2 H) 1.43 - 1.46 (m, 1 H) 1.35 - 1.41 (m, 2 H) 1.18 - 1.31 (m, 2 H) 1.06 - 1.18 (m, 4 H) 0.86 - 1.06 (m, 11 H) 0.59 - 0.86 (m, 9 H) **m/z** calcd $C_{37}H_{69}N_7O$ $[M+H]^+$ 628.6, found 628.9 (MS ESI) **Purity** LC-MS: >98% (214 nm, peak area).

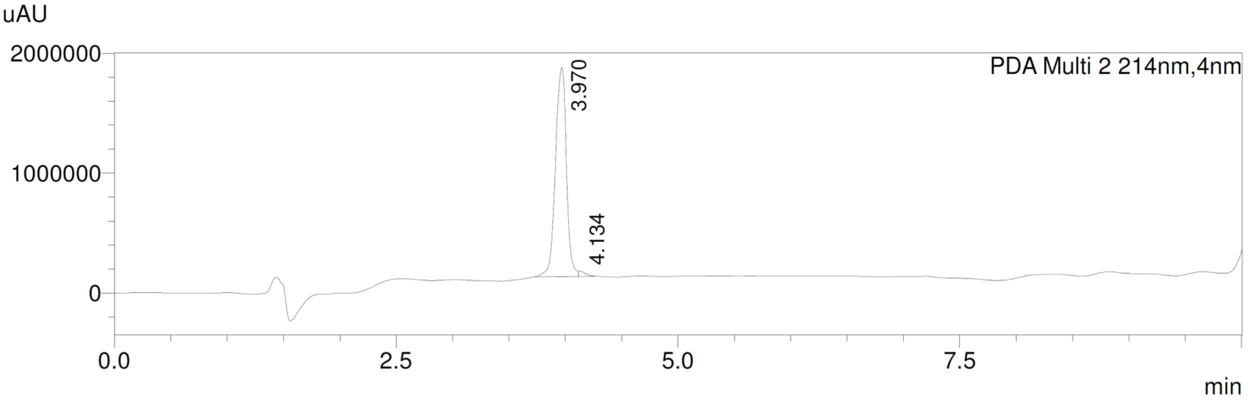

PDA Ch2 214nm

Peak#	Ret. Time	Area	Height	Area%	Conc.
1	3.970	11767788	1745486	98.595	98.595
2	4.134	167655	43003	1.405	1.405
Total		11935443	1788490	100.000	

2718.046 (S)-1-((S)-3-((4-(tert-butyl)cyclohexyl)methyl)-1-((S)-1-cyclohexyl-3-((S)-2-(((R)-2-imino-5-isobutylimidazolidin-1-yl)methyl)pyrrolidin-1-yl)propan-2-yl)-2-iminoimidazolidin-4-yl)ethanol. Compound 2718.046 was synthesized using the following reagents: (100mg) MBHA resin starting material, Boc-D-Leucine-OH (R_1), Boc-L-Proline-OH (R_2), Boc-L-Cyclohexylalanine-OH (R_3), Boc-D-Threonine(Bzl)-OH (R_4), and 4-tert-Butylcyclohexancecarboxylic acid (R_5). The final crude product was purified using HPLC as described above, with a gradient of (B) 0/2, 2/2, 20/27, 25/30, 27/98, 30/98. **Isolated Mass** 6.4mg, **^{1}H NMR** (400 MHz, DMSO-d6) d ppm 10.37 (br. s., 1 H) 8.41 (br. s., 1 H) 3.81 - 4.10 (m, 5 H) 3.55 - 3.81 (m, 5 H) 3.48 (dd, J=14.73, 7.89 Hz, 1 H) 3.29 - 3.42 (m, 2 H) 3.05 - 3.29 (m, 3 H) 2.87 - 3.05 (m, 1 H) 2.66 - 2.87 (m, 2 H) 2.23 - 2.48 (m, 2 H) 2.11 (br. s., 1 H) 1.76 - 1.97 (m, 2 H) 1.64 - 1.76 (m, 5 H) 1.35 - 1.64 (m, 11 H) 1.05 - 1.21 (m, 5 H) 0.86 - 1.04 (m, 11 H) 0.57 - 0.86 (m, 10 H) **m/z** calcd $C_{37}H_{69}N_7O$ $[M+H]^+$ 628.6, found 629.0 (MS ESI) **Purity** LC-MS: >96% (214 nm, peak area).

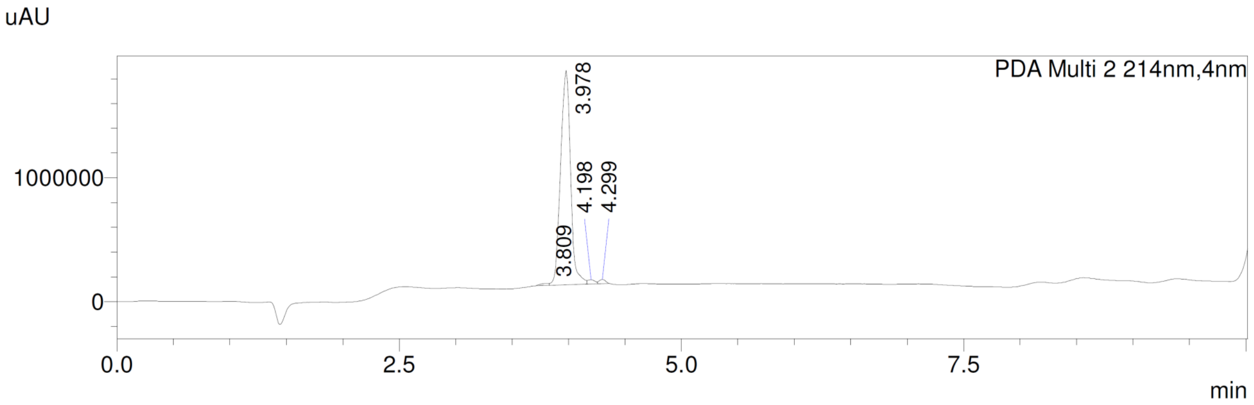

PDA Ch2 214nm

Peak#	Ret. Time	Area	Height	Area%	Conc.
1	3.809	82922	14876	0.753	0.753
2	3.978	10635030	1731798	96.553	96.553
3	4.198	162543	36484	1.476	1.476
4	4.299	134202	35791	1.218	1.218
Total		11014696	1818950	100.000	

2718.047 (S)-1-((S)-3-((4-(tert-butyl)cyclohexyl)methyl)-1-((R)-1-cyclohexyl-3-((R)-2-(((R)-2-imino-5-isobutylimidazolidin-1-yl)methyl)pyrrolidin-1-yl)propan-2-yl)-2-iminoimidazolidin-4-yl)ethanol. Compound 2718.047 was synthesized using the following reagents: (100mg) MBHA resin starting material, Boc-D-Leucine-OH (R_1), Boc-D-Proline-OH (R_2), Boc-D-Cyclohexylalanine-OH (R_3), Boc-D-Threonine(Bzl)-OH (R_4), and 4-tert-Butylcyclohexancecarboxylic acid (R_5). The final crude product was purified using HPLC as described above, with a gradient of (B) 0/2, 2/2, 20/27, 25/30, 27/98, 30/98. **Isolated Mass** 7.5mg, **^{1}H NMR** (400 MHz, DMSO-d6) d ppm 8.29 (br. s., 1 H) 3.86 - 3.99 (m, 2 H) 3.58 - 3.86 (m, 4 H) 3.32 - 3.58 (m, 13 H) 3.05 - 3.32 (m, 6 H) 2.81 - 3.05 (m, 2 H) 2.63 - 2.81 (m, 1 H) 2.22 - 2.48 (m, 2 H) 2.16 (br. s., 1 H) 1.78 - 2.03 (m, 2 H) 1.64 - 1.72 (m, 3 H) 1.56 - 1.63 (m, 4 H) 1.43 - 1.53 (m, 4 H) 1.40 (d, J=6.36 Hz, 1 H) 0.88 - 1.18 (m, 14 H) 0.75 - 0.87 (m, 8 H) **m/z** calcd $C_{37}H_{69}N_7O$ $[M+H]^+$ 628.6, found 628. (MS ESI) **Purity** LC-MS: >99% (214 nm, peak area).

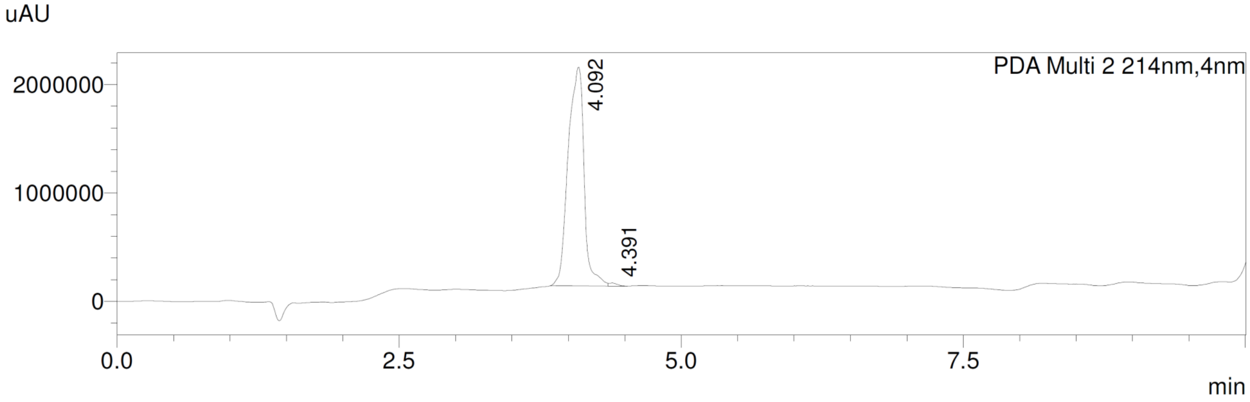

PDA Ch2 214nm

Peak#	Ret. Time	Area	Height	Area%	Conc.
1	4.092	19744725	2017931	99.340	0.000
2	4.391	131174	27216	0.660	0.000
Total		19875898	2045147	100.000	

2718.048 (S)-1-((S)-3-((4-(tert-butyl)cyclohexyl)methyl)-1-((R)-1-cyclohexyl-3-((S)-2-(((S)-2-imino-5-isobutylimidazolidin-1-yl)methyl)pyrrolidin-1-yl)propan-2-yl)-2-iminoimidazolidin-4-yl)ethanol. Compound 2718.048 was synthesized using the following reagents: (100mg) MBHA resin starting material, Boc-L-Leucine-OH (R_1), Boc-L-Proline-OH (R_2), Boc-D-Cyclohexylalanine-OH (R_3), Boc-D-Threonine(Bzl)-OH (R_4), and 4-tert-Butylcyclohexancecarboxylic acid (R_5). The final crude product was purified using HPLC as described above, with a gradient of (B) 0/2, 2/2, 20/27, 25/30, 27/98, 30/98. **Isolated Mass** 7.2mg, **^{1}H NMR** (400 MHz, DMSO-d6) d ppm 8.28 (br. s., 1 H) 4.03 (t, J=9.84 Hz, 1 H) 3.73 - 3.96 (m, 4 H) 3.68 (t, J=9.23 Hz, 1 H) 3.42 - 3.62 (m, 5 H) 3.36 (dd, J=10.09, 3.85 Hz, 5 H) 3.09 - 3.29 (m, 4 H) 2.97 - 3.09 (m, 1 H) 2.90 (dd, J=14.92, 8.44 Hz, 1 H) 2.52 - 2.78 (m, 1 H) 2.09 - 2.30 (m, 3 H) 1.78 - 2.04 (m, 2 H) 1.42 - 1.72 (m, 13 H) 1.39 (d, J=9.29 Hz, 1 H) 1.02 - 1.21 (m, 7 H) 0.84 (s, 8 H) 0.81 - 1.01 (m, 8 H) **m/z** calcd $C_{37}H_{69}N_7O$ $[M+H]^+$ 628.6, found 628.9 (MS ESI) **Purity** LC-MS: >96% (214 nm, peak area).

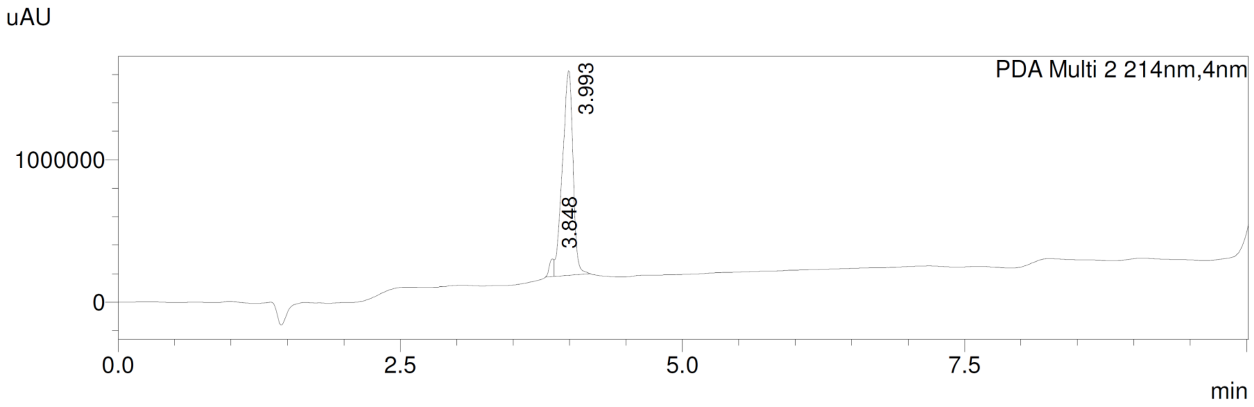

PDA Ch2 214nm

Peak#	Ret. Time	Area	Height	Area%	Conc.
1	3.848	372759	124950	3.844	3.844
2	3.993	9325539	1438128	96.156	96.156
Total		9698297	1563078	100.000	

2718.049 (R)-1-((R)-3-((4-(tert-butyl)cyclohexyl)methyl)-1-((S)-1-cyclohexyl-3-((R)-2-(((S)-2-imino-5-isobutylimidazolidin-1-yl)methyl)pyrrolidin-1-yl)propan-2-yl)-2-iminoimidazolidin-4-yl)ethanol. Compound 2718.049 was synthesized using the following reagents: (100mg) MBHA resin starting material, Boc-L-Leucine-OH (R_1), Boc-D-Proline-OH (R_2), Boc-L-Cyclohexylalanine-OH (R_3), Boc-L-Threonine(Bzl)-OH (R_4), and 4-tert-Butylcyclohexancecarboxylic acid (R_5). The final crude product was purified using HPLC as described above, with a gradient of (B) 0/2, 2/2, 20/27, 25/30, 27/98, 30/98. **Isolated Mass** 7.3mg, **^{1}H NMR** (400 MHz, DMSO-d6) d ppm 8.25 (br. s., 1 H) 3.73 - 4.03 (m, 4 H) 3.66 (t, J=9.29 Hz, 1 H) 3.43 - 3.59 (m, 3 H) 3.37 (br. s., 7 H) 3.09 - 3.34 (m, 7 H) 2.97 - 3.09 (m, 1 H) 2.81 - 2.97 (m, 1 H) 2.67 - 2.81 (m, 1 H) 2.20 - 2.35 (m, 1 H) 2.18 (br. s., 1 H) 1.75 - 1.96 (m, 2 H) 1.63 - 1.75 (m, 4 H) 1.59 (d, J=5.62 Hz, 4 H) 1.31 - 1.54 (m, 6 H) 1.18 - 1.31 (m, 2 H) 1.12 (d, J=7.21 Hz, 3 H) 0.87 - 1.09 (m, 11 H) 0.67 - 0.87 (m, 8 H) **m/z** calcd $C_{37}H_{69}N_7O$ $[M+H]^+$ 628.6, found 628.65 (MS ESI) **Purity** LC-MS: >96% (214 nm, peak area).

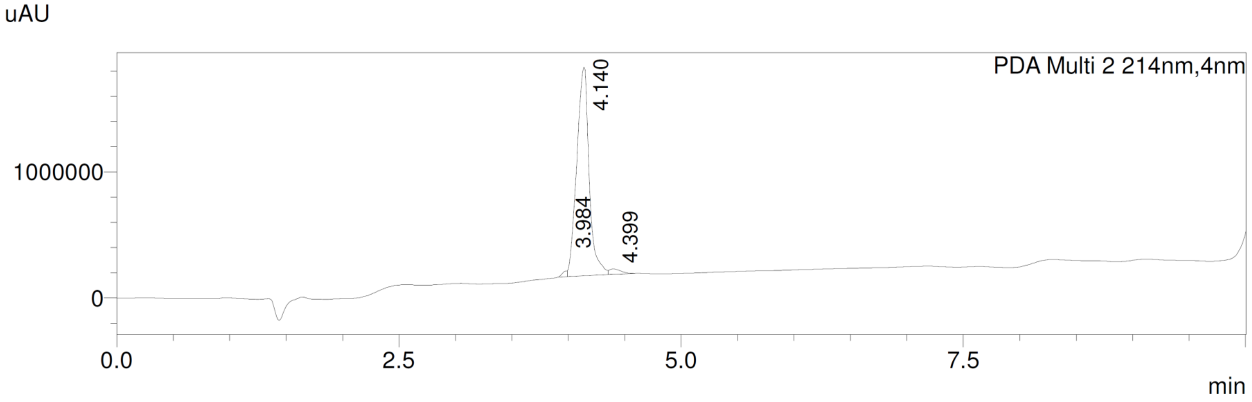

PDA Ch2 214nm

Peak#	Ret. Time	Area	Height	Area%	Conc.
1	3.984	132509	42573	1.014	1.014
2	4.140	12636104	1653554	96.678	96.678
3	4.399	301750	43107	2.309	2.309
Total		13070364	1739233	100.000	

2718.050 (S)-3-((4-(tert-butyl)cyclohexyl)methyl)-1-((R)-1-cyclohexyl-3-((S)-2-(((R)-2-imino-5-isopropylimidazolidin-1-yl)methyl)pyrrolidin-1-yl)propan-2-yl)-4-propylimidazolidin-2-imine. Compound 2718.050 was synthesized using the following reagents: (100mg) MBHA resin starting material, Boc-D-Valine-OH (R_1), Boc-L-Proline-OH (R_2), Boc-D-Cyclohexylalanine-OH (R_3), Boc-L-Norvaline-OH (R_4), and 4-tert-Butylcyclohexancecarboxylic acid (R_5). The final crude product was purified using HPC as described above, with a gradient of (B) 0/2, 2/2, 20/27, 25/30, 27/98, 30/98. **Isolated Mass** 6.9mg, **^{1}H NMR** (400 MHz, DMSO-d6) d ppm 10.38 (br. s., 1 H) 8.28 (br. s., 1 H) 3.97 - 4.14 (m, 1 H) 3.84 - 3.97 (m, 2 H) 3.62 - 3.84 (m, 2 H) 3.32 - 3.62 (m, 7 H) 3.21 - 3.31 (m, 2 H) 3.05 - 3.20 (m, 2 H) 2.99 (dd, J=14.67, 5.99 Hz, 1 H) 2.63 - 2.92 (m, 2 H) 2.15 - 2.36 (m, 2 H) 2.09 (dt, J=6.91, 3.39 Hz, 1 H) 1.73 - 1.94 (m, 2 H) 1.57 - 1.73 (m, 8 H) 1.32 - 1.54 (m, 6 H) 1.23 - 1.31 (m, 3 H) 1.09 - 1.21 (m, 5 H) 0.84 (s, 7 H) 0.73 - 1.02 (m, 13 H) **m/z** calcd $C_{37}H_{69}N_7$ $[M+H]^+$ 612.6, found 612.65 (MS ESI) **Purity** LC-MS: >95% (214 nm, peak area).

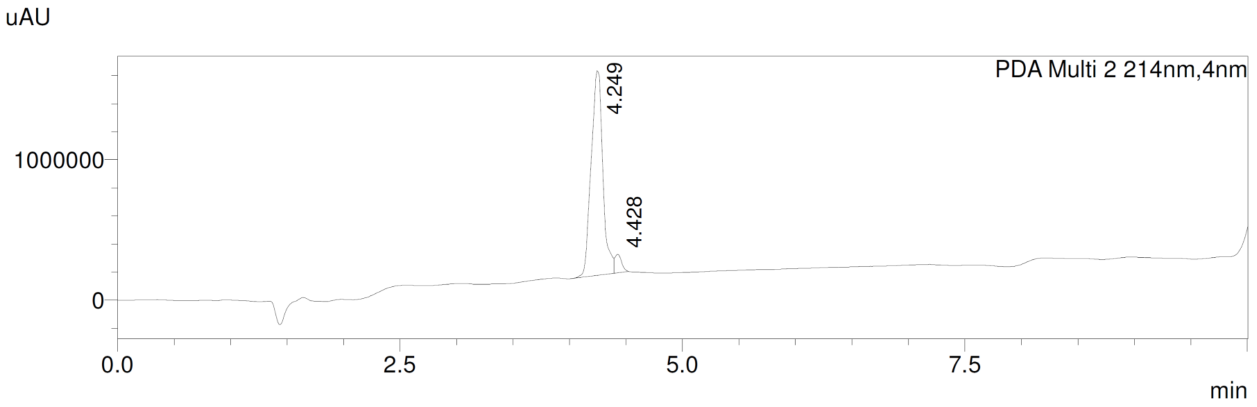

PDA Ch2 214nm

Peak#	Ret. Time	Area	Height	Area%	Conc.
1	4.249	10513682	1459516	95.213	95.213
2	4.428	528618	132762	4.787	4.787
Total		11042300	1592278	100.000	

2718.051 (R)-3-((4-(tert-butyl)cyclohexyl)methyl)-1-((S)-1-cyclohexyl-3-((S)-2-(((R)-2-imino-5-isopropylimidazolidin-1-yl)methyl)pyrrolidin-1-yl)propan-2-yl)-4-propylimidazolidin-2-imine. Compound 2718.051 was synthesized using the following reagents: (100mg) MBHA resin starting material, Boc-D-Valine-OH (R_1), Boc-L-Proline-OH (R_2), Boc-L-Cyclohexylalanine-OH (R_3), Boc-D-Norvaline-OH (R_4), and 4-tert-Butylcyclohexancecarboxylic acid (R_5). The final crude product was purified using HPLC as described above, with a gradient of (B) 0/2, 2/2, 20/27, 25/30, 27/98, 30/98. **Isolated Mass** 6.6mg, **^{1}H NMR** (400 MHz, DMSO-d6) d ppm 8.39 (br. s., 1 H) 3.79 - 4.08 (m, 3 H) 3.56 - 3.79 (m, 2 H) 3.42 - 3.56 (m, 3 H) 3.30 - 3.42 (m, 12 H) 3.25 (dd, J=10.09, 4.34 Hz, 2 H) 2.94 - 3.17 (m, 3 H) 2.85 - 2.92 (m, 1 H) 2.67 - 2.80 (m, 1 H) 2.32 - 2.48 (m, 2 H) 2.13 (td, J=6.72, 3.67 Hz, 1 H) 2.03 (br. s., 1 H) 1.77 - 1.90 (m, 2 H) 1.58 - 1.75 (m, 6 H) 1.33 - 1.54 (m, 7 H) 1.27 (dd, J=15.04, 7.46 Hz, 1 H) 1.03 - 1.21 (m, 5 H) 0.84 (s, 8 H) 0.81 - 1.03 (m, 6 H) 0.77 (d, J=6.72 Hz, 3 H) **m/z** calcd $C_{37}H_{69}N_7$ $[M+H]^+$ 612.6, found 613.0 (MS ESI) **Purity** LC-MS: >99% (214 nm, peak area).

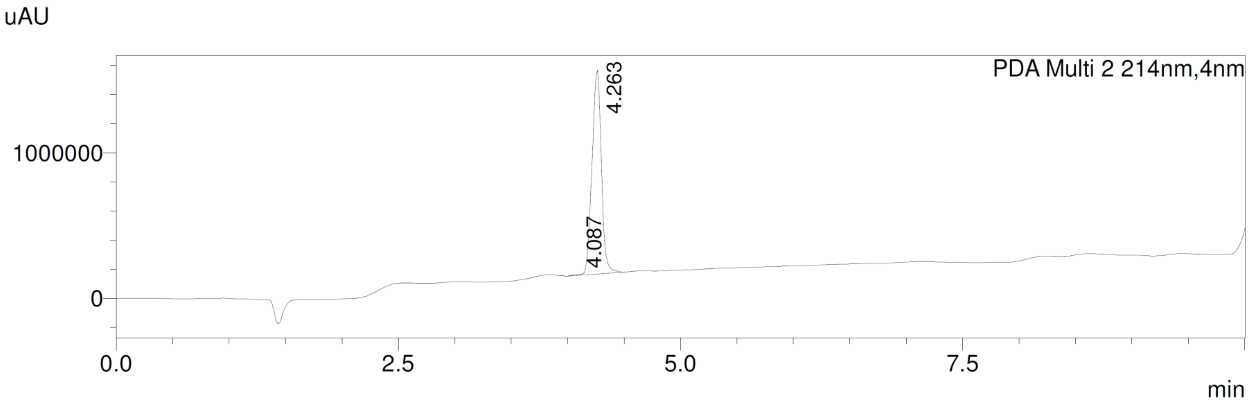

PDA Ch2 214nm

Peak#	Ret. Time	Area	Height	Area%	Conc.
1	4.087	35387	7045	0.438	0.438
2	4.263	8038237	1403566	99.562	99.562
Total		8073624	1410610	100.000	

2718.052 (R)-3-((4-(tert-butyl)cyclohexyl)methyl)-1-((R)-1-cyclohexyl-3-((R)-2-(((R)-2-imino-5-isopropylimidazolidin-1-yl)methyl)pyrrolidin-1-yl)propan-2-yl)-4-propylimidazolidin-2-imine. Compound 2718.052 was synthesized using the following reagents: (100mg) MBHA resin starting material, Boc-D-Valine-OH (R_1), Boc-D-Proline-OH (R_2), Boc-D-Cyclohexylalanine-OH (R_3), Boc-D-Norvaline-OH (R_4), and 4-tert-Butylcyclohexancecarboxylic acid (R_5). The final crude product was purified using HPLC as described above, with a gradient of (B) 0/2, 2/2, 20/27, 25/30, 27/98, 30/98. **Isolated Mass** 6.5mg, **^{1}H NMR** (400 MHz, DMSO-d6) d ppm 8.42 (br. s., 1 H) 4.04 (dt, J=9.26, 4.72 Hz, 1 H) 3.96 (br. s., 1 H) 3.67 - 3.92 (m, 2 H) 3.31 - 3.61 (m, 11 H) 3.16 - 3.31 (m, 2 H) 2.93 - 3.16 (m, 3 H) 2.65 - 2.93 (m, 2 H) 2.22 - 2.40 (m, 2 H) 2.15 (td, J=6.60, 3.79 Hz, 1 H) 2.07 (br. s., 1 H) 1.78 - 1.95 (m, 2 H) 1.56 - 1.78 (m, 7 H) 1.33 - 1.56 (m, 7 H) 1.02 - 1.30 (m, 6 H) 0.72 - 1.02 (m, 19 H) **m/z** calcd $C_{37}H_{69}N_7$ $[M+H]^+$ 612.6, found 612.65 (MS ESI) **Purity** LC-MS: >98% (214 nm, peak area).

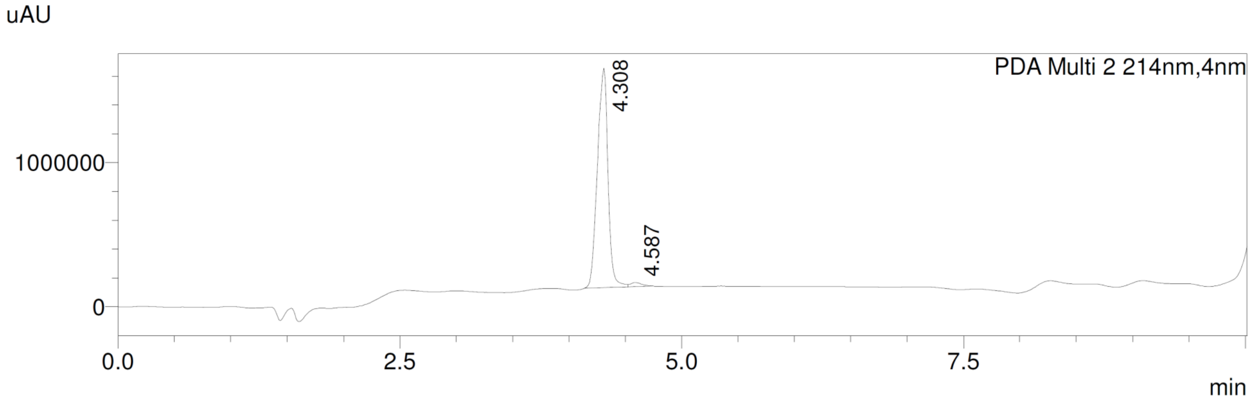

PDA Ch2 214nm

Peak#	Ret. Time	Area	Height	Area%	Conc.
1	4.308	9941972	1522385	98.057	98.057
2	4.587	196973	27430	1.943	1.943
Total		10138946	1549815	100.000	

2718.053 (R)-3-((4-(tert-butyl)cyclohexyl)methyl)-1-((R)-1-cyclohexyl-3-((S)-2-(((S)-2-imino-5-isopropylimidazolidin-1-yl)methyl)pyrrolidin-1-yl)propan-2-yl)-4-propylimidazolidin-2-imine. Compound 2718.053 was synthesized using the following reagents: (100mg) MBHA resin starting material, Boc-L-Valine-OH (R_1), Boc-L-Proline-OH (R_2), Boc-D-Cyclohexylalanine-OH (R_3), Boc-D-Norvaline-OH (R_4), and 4-tert-Butylcyclohexancecarboxylic acid (R_5). The final crude product was purified using HPLC as described above, with a gradient of (B) 0/2, 2/2, 20/27, 25/30, 27/98, 30/98. **Isolated Mass** 6.8mg, **^{1}H NMR** (400 MHz, DMSO-d6) d ppm 8.29 (br. s., 1 H) 3.97 - 4.05 (m, 1 H) 3.73 - 3.95 (m, 3 H) 3.52 - 3.60 (m, 2 H) 3.29 - 3.50 (m, 8 H) 3.04 - 3.19 (m, 2 H) 2.97 (dd, J=14.86, 5.69 Hz, 1 H) 2.59 - 2.88 (m, 2 H) 2.13 - 2.42 (m, 2 H) 2.02 - 2.13 (m, 2 H) 1.80 - 2.02 (m, 2 H) 1.57 - 1.80 (m, 8 H) 1.32 - 1.55 (m, 7 H) 1.22 - 1.30 (m, 2 H) 1.07 - 1.21 (m, 5 H) 0.80 - 1.05 (m, 17 H) 0.75 (d, J=6.85 Hz, 3 H) **m/z** calcd $C_{37}H_{69}N_7$ $[M+H]^+$ 612.6, found 613.0 (MS ESI) **Purity** LC-MS: >96% (214 nm, peak area).

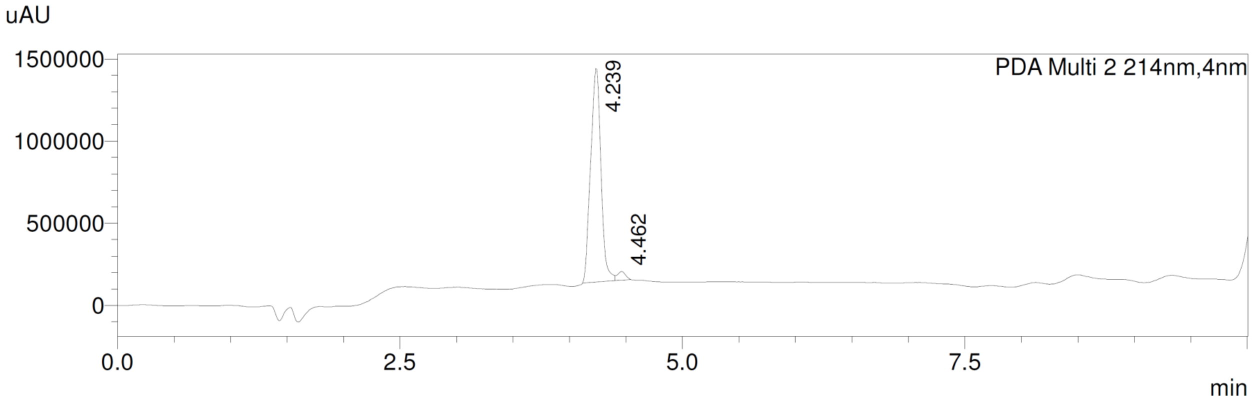

PDA Ch2 214nm

Peak#	Ret. Time	Area	Height	Area%	Conc.
1	4.239	8276647	1301200	96.949	96.949
2	4.462	260469	53744	3.051	3.051
Total		8537116	1354944	100.000	

2718.054 (S)-3-((4-(tert-butyl)cyclohexyl)methyl)-1-((S)-1-cyclohexyl-3-((R)-2-(((S)-2-imino-5-isopropylimidazolidin-1-yl)methyl)pyrrolidin-1-yl)propan-2-yl)-4-propylimidazolidin-2-imine. Compound 2718.054 was synthesized using the following reagents: (100mg) MBHA resin starting material, Boc-L-Valine-OH (R_1), Boc-D-Proline-OH (R_2), Boc-L-Cyclohexylalanine-OH (R_3), Boc-L-Norvaline-OH (R_4), and 4-tert-Butylcyclohexancecarboxylic acid (R_5). The final crude product was purified using HPLC as described above, with a gradient of (B) 0/2, 2/2, 20/27, 25/30, 27/98, 30/98. **Isolated Mass** 7.0mg, **^{1}H NMR** (400 MHz, DMSO-d6) d ppm 8.31 (br. s., 1 H) 3.86 - 4.07 (m, 2 H) 3.79 (dd, J=14.86, 10.94 Hz, 2 H) 3.42 - 3.69 (m, 5 H) 3.38 (br. s., 11 H) 3.09 - 3.34 (m, 5 H) 2.81 - 3.07 (m, 3 H) 2.11 - 2.35 (m, 2 H) 2.08 (br. s., 1 H) 1.64 - 1.92 (m, 5 H) 1.61 (br. s., 2 H) 1.34 - 1.56 (m, 6 H) 1.05 - 1.32 (m, 6 H) 0.84 (s, 7 H) 0.80 - 1.05 (m, 6 H) 0.74 (d, J=6.72 Hz, 2 H) **m/z** calcd $C_{37}H_{69}N_7$ $[M+H]^+$ 612.6, found 612.95 (MS ESI) **Purity** LC-MS: >98% (214 nm, peak area).

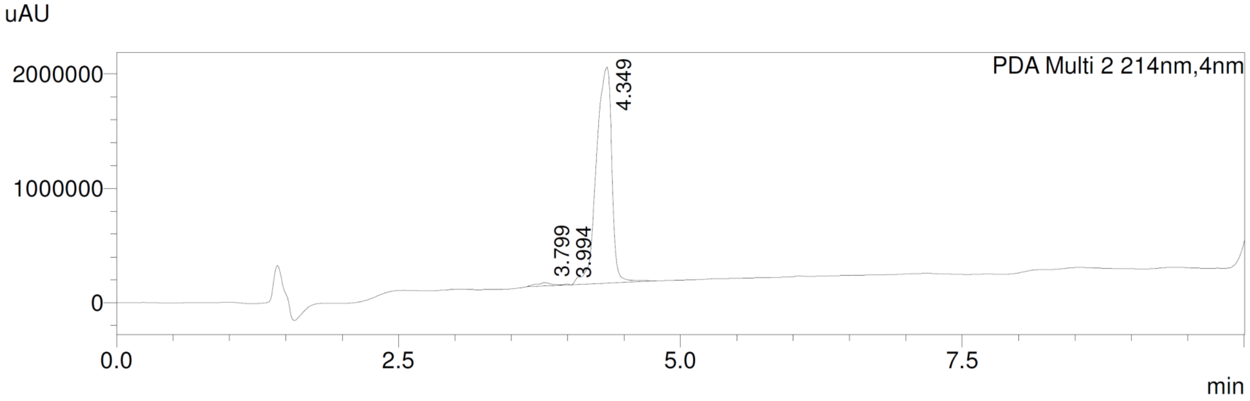

PDA Ch2 214nm

Peak#	Ret. Time	Area	Height	Area%	Conc.
1	3.799	261366	28444	1.435	1.435
2	3.994	27753	9816	0.152	0.152
3	4.349	17927279	1889802	98.413	98.413
Total		18216398	1928063	100.000	

2718.055 (S)-1-((S)-3-((4-(tert-butyl)cyclohexyl)methyl)-2-imino-1-((R)-1-((S)-2-(((R)-2-imino-5-isobutylimidazolidin-1-yl)methyl)pyrrolidin-1-yl)-3-(naphthalen-2-yl)propan-2-yl)imidazolidin-4-yl)ethanol. Compound 2718.055 was synthesized using the following reagents: (100mg) MBHA resin starting material, Boc-D-Leucine-OH (R_1), Boc-L-Proline-OH (R_2), Boc-D-2-Napthylalanine-OH (R_3), Boc-D-Threonine(Bzl)-OH (R_4), and 4-tert-Butylcyclohexancecarboxylic acid (R_5). The final crude product was purified using HPLC as described above, with a gradient of (B) 0/2, 2/2, 20/27, 25/30, 27/98, 30/98. **Isolated Mass** 6.8mg, **^{1}H NMR** (400 MHz, DMSO-d6) d ppm 7.99 (br. s., 1 H) 7.71 - 7.93 (m, 4 H) 7.52 - 7.63 (m, 1 H) 7.21 - 7.50 (m, 2 H) 4.43 - 4.59 (m, 1 H) 3.79 - 4.04 (m, 2 H) 3.50 - 3.79 (m, 5 H) 3.28 - 3.50 (m, 6 H) 3.02 - 3.28 (m, 5 H) 2.85 - 3.02 (m, 2 H) 2.78 (dd, J=7.64, 3.73 Hz, 1 H) 2.45 (br. s., 1 H) 2.10 - 2.40 (m, 1 H) 1.77 - 1.95 (m, 1 H) 1.56 - 1.77 (m, 4 H) 1.35 - 1.56 (m, 2 H) 1.31 (d, J=11.49 Hz, 1 H) 1.21 (d, J=14.31 Hz, 1 H) 0.87 - 1.11 (m, 9 H) 0.83 (d, J=6.24 Hz, 1 H) 0.55 - 0.78 (m, 10 H) **m/z** calcd $C_{41}H_{65}N_7O$ $[M+H]^+$ 672.5, found 672.6 (MS ESI) **Purity** LC-MS: >96% (214 nm, peak area).

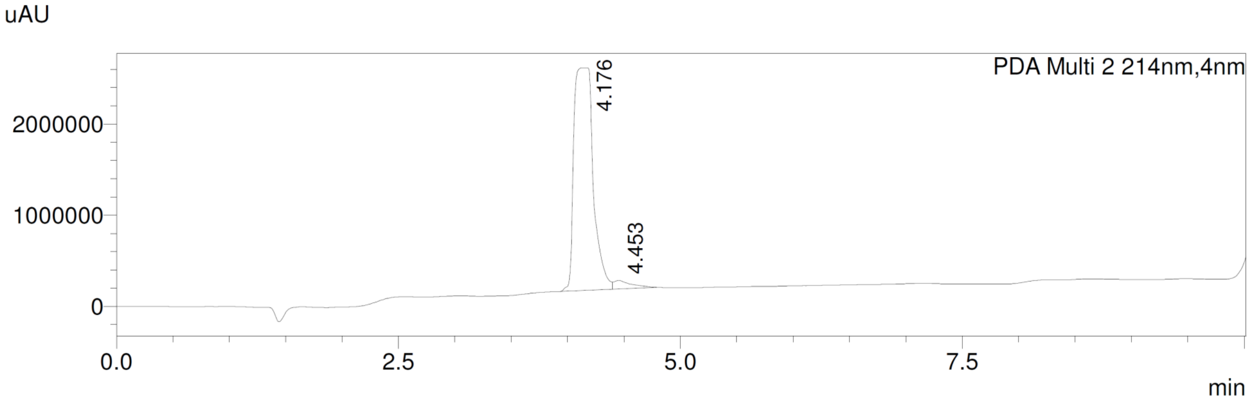

PDA Ch2 214nm

Peak#	Ret. Time	Area	Height	Area%	Conc.
1	4.176	29124056	2441848	96.671	96.671
2	4.453	1002828	93208	3.329	3.329
Total		30126884	2535056	100.000	

2718.056 (S)-1-((S)-3-((4-(tert-butyl)cyclohexyl)methyl)-2-imino-1-((R)-1-((S)-2-(((R)-2-imino-5-isobutylimidazolidin-1-yl)methyl)pyrrolidin-1-yl)-3-(1H-indol-3-yl)propan-2-yl)imidazolidin-4-yl)ethanol. Compound 2718.056 was synthesized using the following reagents: (100mg) MBHA resin starting material, Boc-D-Leucine-OH (R_1), Boc-L-Proline-OH (R_2), Boc-D-Tryptophan-OH (R_3), Boc-D-Threonine(Bzl)-OH (R_4), and 4-tert-Butylcyclohexancecarboxylic acid (R_5). The final crude product was purified using HPLC as described above, with a gradient of (B) 0/2, 2/2, 20/27, 25/30, 27/98, 30/98. **Isolated Mass** 6.4mg, **^{1}H NMR** (400 MHz, DMSO-d6) d ppm 8.08 - 8.27 (m, 1 H) 7.14 - 7.25 (m, 1 H) 6.93 - 7.13 (m, 1 H) 6.87 (t, J=7.89 Hz, 1 H) 3.89 - 3.99 (m, 1 H) 3.73 - 3.86 (m, 2 H) 3.65 (d, J=9.17 Hz, 1 H) 3.60 (d, J=5.50 Hz, 1 H) 3.28 - 3.54 (m, 8 H) 3.14 - 3.27 (m, 3 H) 2.84 - 3.14 (m, 4 H) 2.76 (br. s., 1 H) 2.45 (br. s., 1 H) 2.17 - 2.41 (m, 1 H) 2.08 (s, 1 H) 1.84 (dd, J=12.10, 8.07 Hz, 1 H) 1.63 - 1.76 (m, 2 H) 1.60 (br. s., 2 H) 1.30 - 1.56 (m, 4 H) 1.04 (d, J=6.11 Hz, 10 H) 0.99 - 1.09 (m, 1 H) 0.77 - 0.97 (m, 13 H) **m/z** calcd $C_{39}H_{64}N_8O$ $[M+H]^+$ 661.5, found 661.4 (MS ESI) **Purity** LC-MS: >99% (214 nm, peak area).

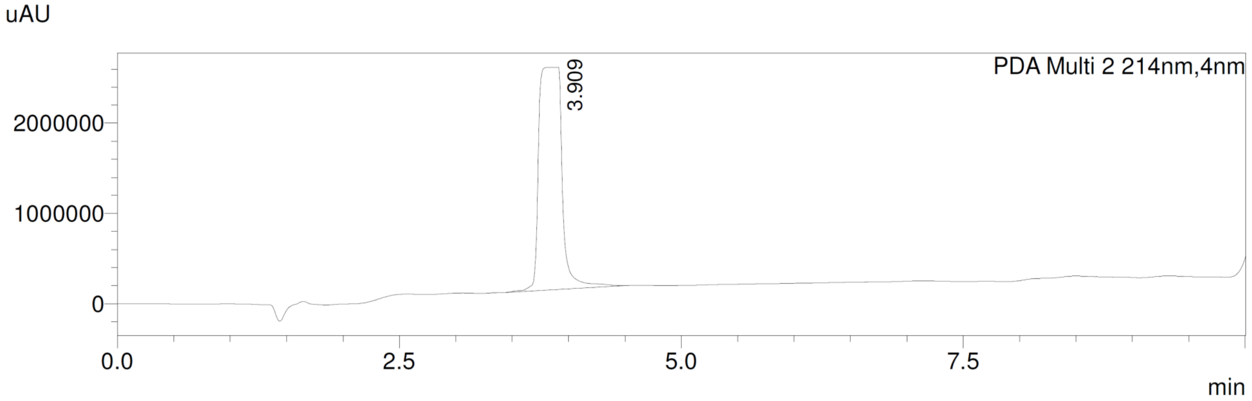

PDA Ch2 214nm

Peak#	Ret. Time	Area	Height	Area%	Conc.
1	3.909	34845962	2459477	100.000	100.000
Total		34845962	2459477	100.000	

2718.057 4-((R)-2-((S)-3-((4-(tert-butyl)cyclohexyl)methyl)-4-((S)-1-hydroxyethyl)-2-iminoimidazolidin-1-yl)-3-((S)-2-(((R)-2-imino-5-isobutylimidazolidin-1-yl)methyl)pyrrolidin-1-yl)propyl)phenol. Compound 2718.057 was synthesized using the following reagents: (100mg) MBHA resin starting material, Boc-D-Leucine-OH (R_1), Boc-L-Proline-OH (R_2), Boc-D-Tyrosine-OH (R_3), Boc-D-Threonine(Bzl)-OH (R_4), and 4-tert-Butylcyclohexancecarboxylic acid (R_5). The final crude product was purified using HPLC as described above, with a gradient of (B) 0/2, 2/2, 20/26, 25/29, 27/98, 30/98. **Isolated Mass** 6.0mg, **^{1}H NMR** (400 MHz, DMSO-d6) d ppm 8.44 (br. s., 1 H) 7.92 (br. s., 1 H) 7.08 (d, J=8.44 Hz, 1 H) 6.65 (d, J=8.44 Hz, 1 H) 4.19 (d, J=4.65 Hz, 2 H) 3.73 - 3.95 (m, 5 H) 3.46 - 3.71 (m, 5 H) 3.15 - 3.43 (m, 4 H) 3.10 (dd, J=14.73, 6.54 Hz, 1 H) 2.95 - 3.05 (m, 1 H) 2.74 (dd, J=13.69, 4.52 Hz, 1 H) 2.63 (dd, J=14.06, 9.90 Hz, 1 H) 2.36 (dd, J=12.53, 3.48 Hz, 1 H) 2.11 - 2.30 (m, 1 H) 1.74 - 1.96 (m, 1 H) 1.54 - 1.74 (m, 3 H) 1.29 - 1.54 (m, 4 H) 1.11 - 1.29 (m, 1 H) 1.04 (d, J=6.11 Hz, 19 H) 0.80 - 1.10 (m, 6 H) **m/z** calcd $C_{37}H_{63}N_7O_2$ $[M+H]^+$ 638.5, found 638.4 (MS ESI) **Purity** LC-MS: >98% (214 nm, peak area).

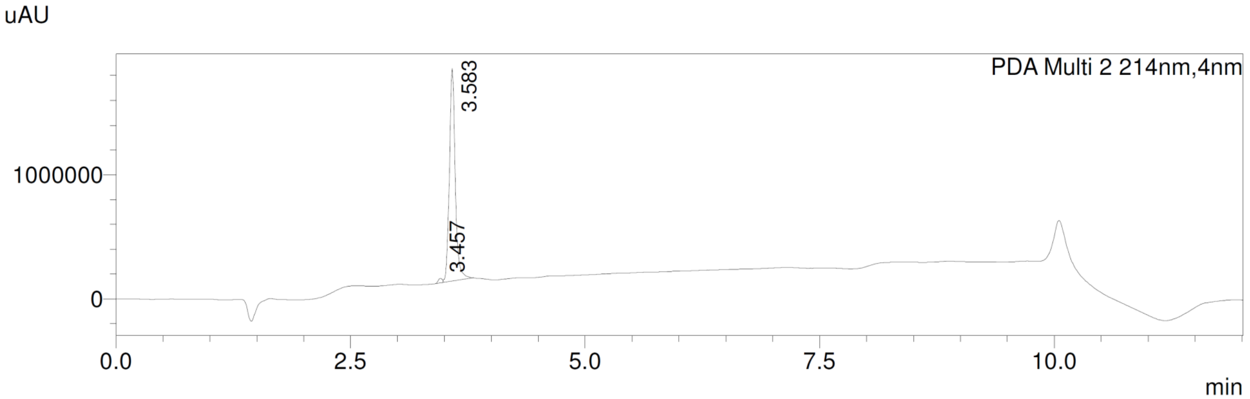

PDA Ch2 214nm

Peak#	Ret. Time	Area	Height	Area%	Conc.
1	3.457	120191	35924	1.595	1.595
2	3.583	7414747	1716757	98.405	98.405
Total		7534938	1752681	100.000	

2718.059 (S)-1-((S)-3-((4-(tert-butyl)cyclohexyl)methyl)-1-((R)-1-cyclohexyl-2-((S)-2-(((R)-2-imino-5-isobutylimidazolidin-1-yl)methyl)pyrrolidin-1-yl)ethyl)-2-iminoimidazolidin-4-yl)ethanol. Compound 2718.059 was synthesized using the following reagents: (100mg) MBHA resin starting material, Boc-D-Leucine-OH (R_1), Boc-L-Proline-OH (R_2), Boc-D-Cyclohexylglycine-OH (R_3), Boc-D-Threonine(Bzl)-OH (R_4), and 4-tert-Butylcyclohexancecarboxylic acid (R_5). The final crude product was purified using HPLC as described above, with a gradient of (B) 0/2, 2/2, 20/27, 25/30, 27/98, 30/98. **Isolated Mass** 6.5mg, **^{1}H NMR** (400 MHz, DMSO-d6) d ppm 8.25 (br. s., 1 H) 3.86 - 3.95 (m, 2 H) 3.63 - 3.81 (m, 6 H) 3.43 - 3.62 (m, 4 H) 3.13 - 3.43 (m, 6 H) 2.79 - 3.06 (m, 2 H) 2.68 (dt, J=3.76, 1.85 Hz, 1 H) 2.43 (dd, J=12.41, 3.24 Hz, 1 H) 1.96 - 2.25 (m, 2 H) 1.78 - 1.93 (m, 1 H) 1.54 - 1.78 (m, 7 H) 1.47 - 1.54 (m, 3 H) 1.28 - 1.47 (m, 4 H) 1.04 (d, J=6.11 Hz, 12 H) 1.02 - 1.21 (m, 2 H) 0.92 (dd, J=12.65, 5.93 Hz, 5 H) 0.84 (s, 6 H) **m/z** calcd $C_{36}H_{67}N_7O$ $[M+H]^+$ 614.5, found 614.65 (MS ESI) **Purity** LC-MS: >99% (214 nm, peak area).

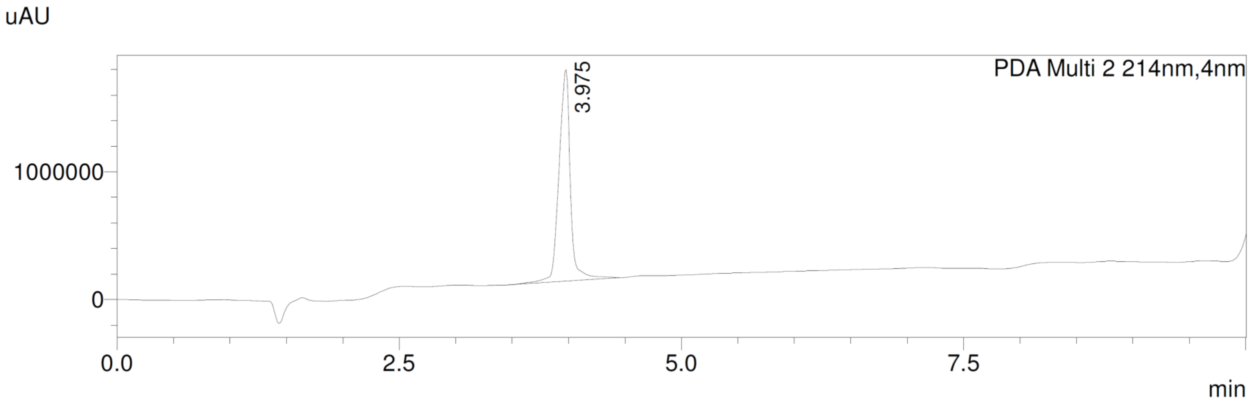

PDA Ch2 214nm

Peak#	Ret. Time	Area	Height	Area%	Conc.
1	3.975	11621561	1655802	100.000	100.000
Total		11621561	1655802	100.000	

2718.060 (S)-1-((S)-3-((4-(tert-butyl)cyclohexyl)methyl)-2-imino-1-((R)-1-((S)-2-(((R)-2-imino-5-isobutylimidazolidin-1-yl)methyl)pyrrolidin-1-yl)hexan-2-yl)imidazolidin-4-yl)ethanol. Compound 2718.060 was synthesized using the following reagents: (100mg) MBHA resin starting material, Boc-D-Leucine-OH (R_1), Boc-L-Proline-OH (R_2), Boc-D-Norleucine-OH (R_3), Boc-D-Threonine(Bzl)-OH (R_4), and 4-tert-Butylcyclohexancecarboxylic acid (R_5). The final crude product was purified using HPLC as described above, with a gradient of (B) 0/2, 2/2, 20/27, 25/30, 27/98, 30/98. **Isolated Mass** 5.4mg, **^{1}H NMR** (400 MHz, DMSO-d6) d ppm 8.27 (br. s., 1 H) 3.84 - 4.03 (m, 3 H) 3.71 - 3.83 (m, 4 H) 3.42 - 3.70 (m, 6 H) 3.13 - 3.42 (m, 6 H) 2.81 - 3.08 (m, 2 H) 2.60 - 2.81 (m, 1 H) 2.18 - 2.38 (m, 1 H) 2.15 (br. s., 1 H) 1.76 - 1.90 (m, 1 H) 1.54 - 1.76 (m, 4 H) 1.34 - 1.54 (m, 6 H) 1.27 (td, J=14.27, 7.52 Hz, 2 H) 1.04 (d, J=6.11 Hz, 13 H) 1.02 - 1.17 (m, 1 H) 0.84 (s, 7 H) 0.81 - 1.00 (m, 6 H) **m/z** calcd $C_{34}H_{65}N_7O$ $[M+H]^+$ 588.9, found 588.9 (MS ESI) **Purity** LC-MS: >99% (214 nm, peak area).

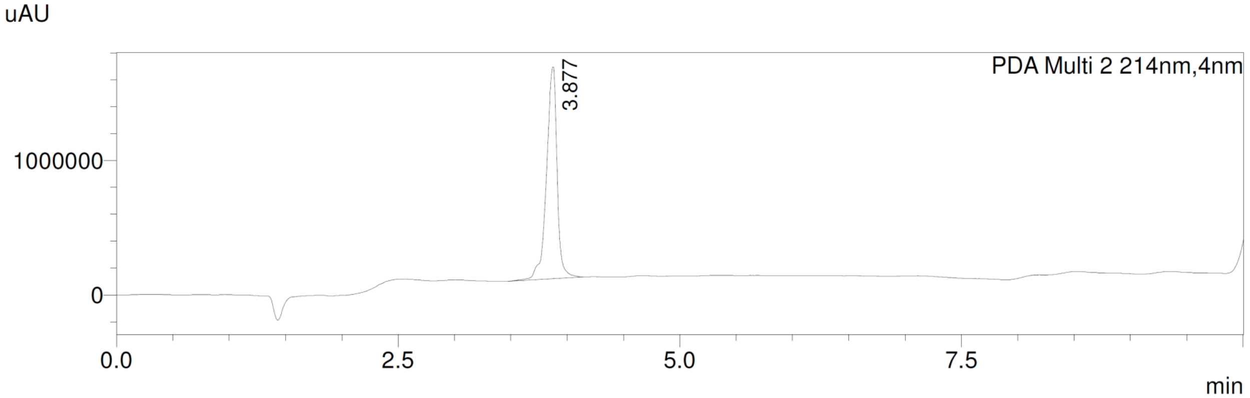

PDA Ch2 214nm

Peak#	Ret. Time	Area	Height	Area%	Conc.
1	3.877	10286842	1574625	100.000	100.000
Total		10286842	1574625	100.000	

2718.061 (S)-1-((S)-3-((4-(tert-butyl)cyclohexyl)methyl)-2-imino-1-((R)-1-((S)-2-(((R)-2-imino-5-isobutylimidazolidin-1-yl)methyl)pyrrolidin-1-yl)propan-2-yl)imidazolidin-4-yl)ethanol. Compound 2718.061 was synthesized using the following reagents: (100mg) MBHA resin starting material, Boc-D-Leucine-OH (R_1), Boc-L-Proline-OH (R_2), Boc-D-Alanine-OH (R_3), Boc-D-Threonine(Bzl)-OH (R_4), and 4-tert-Butylcyclohexancecarboxylic acid (R_5). The final crude product was purified using HPLC as described above, with a gradient of (B) 0/2, 2/2, 20/26, 25/29, 27/98, 30/98. **Isolated Mass** 6.3mg, **^{1}H NMR** (400 MHz, DMSO-d6) d ppm 8.44 (br. s., 2 H) 4.02 - 4.32 (m, 1 H) 3.85 - 4.02 (m, 2 H) 3.52 - 3.81 (m, 8 H) 3.28 - 3.49 (m, 4 H) 3.12 - 3.28 (m, 3 H) 2.83 - 3.01 (m, 2 H) 2.58 - 2.83 (m, 1 H) 2.16 - 2.35 (m, 2 H) 2.04 - 2.16 (m, 1 H) 1.76 - 1.94 (m, 1 H) 1.66 - 1.73 (m, 1 H) 1.35 - 1.64 (m, 7 H) 1.04 (d, J=6.11 Hz, 13 H) 1.01 - 1.28 (m, 1 H) 0.84 (s, 6 H) 0.79 - 1.01 (m, 4 H) **m/z** calcd $C_{31}H_{59}N_7O$ $[M+H]^+$ 546.5, found 546.65 (MS ESI) **Purity** LC-MS: >97% (214 nm, peak area).

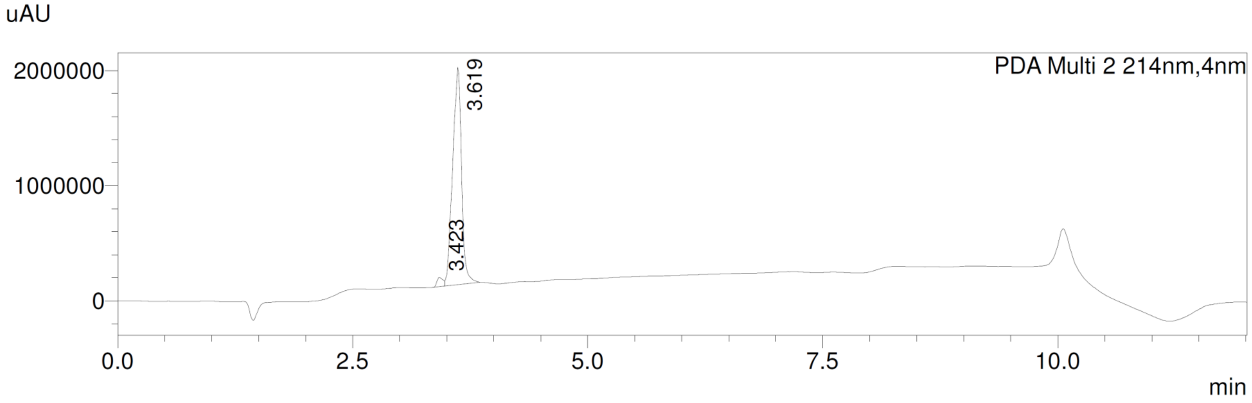

PDA Ch2 214nm

Peak#	Ret. Time	Area	Height	Area%	Conc.
1	3.423	371550	81843	2.912	2.912
2	3.619	12389467	1889955	97.088	97.088
Total		12761017	1971798	100.000	

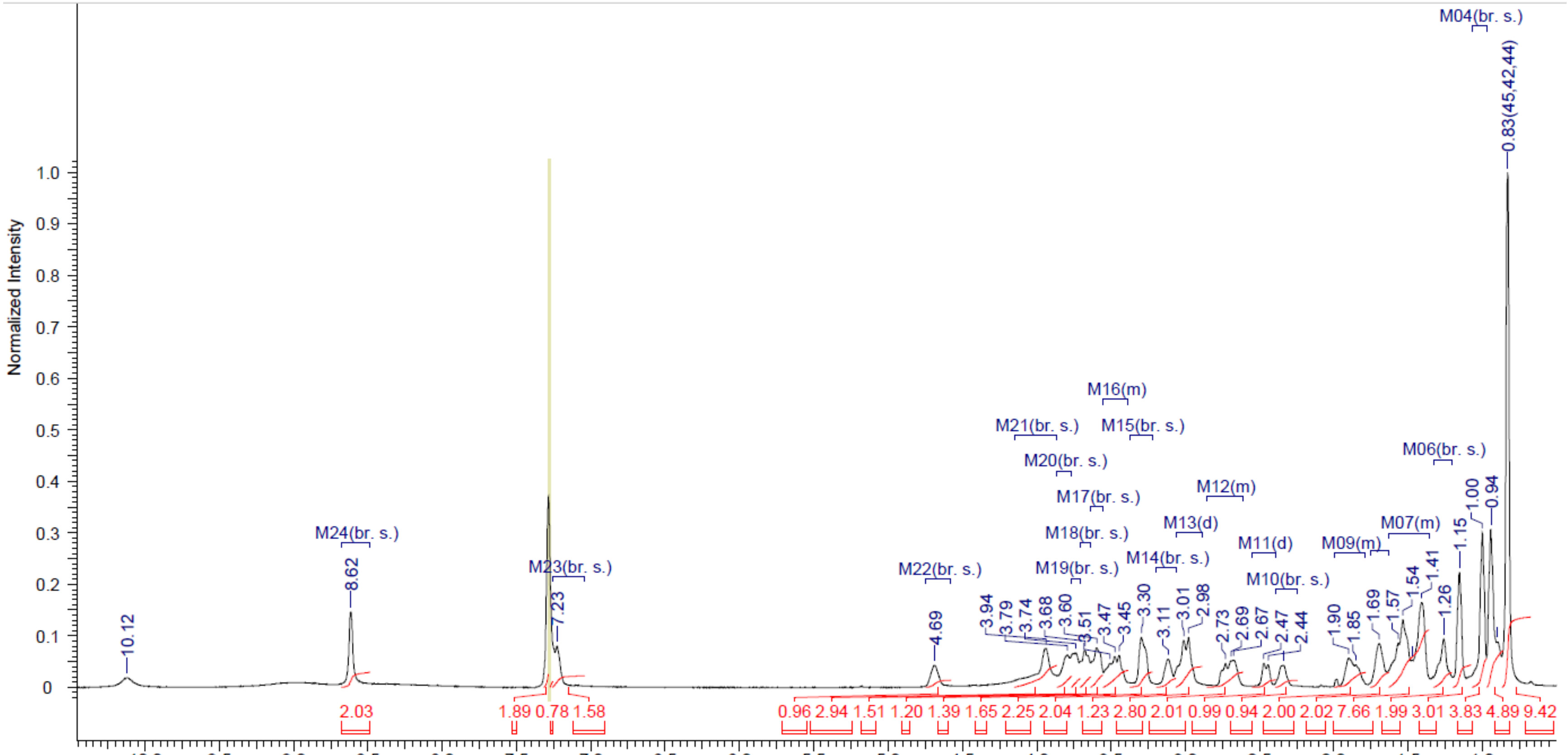

2718.001

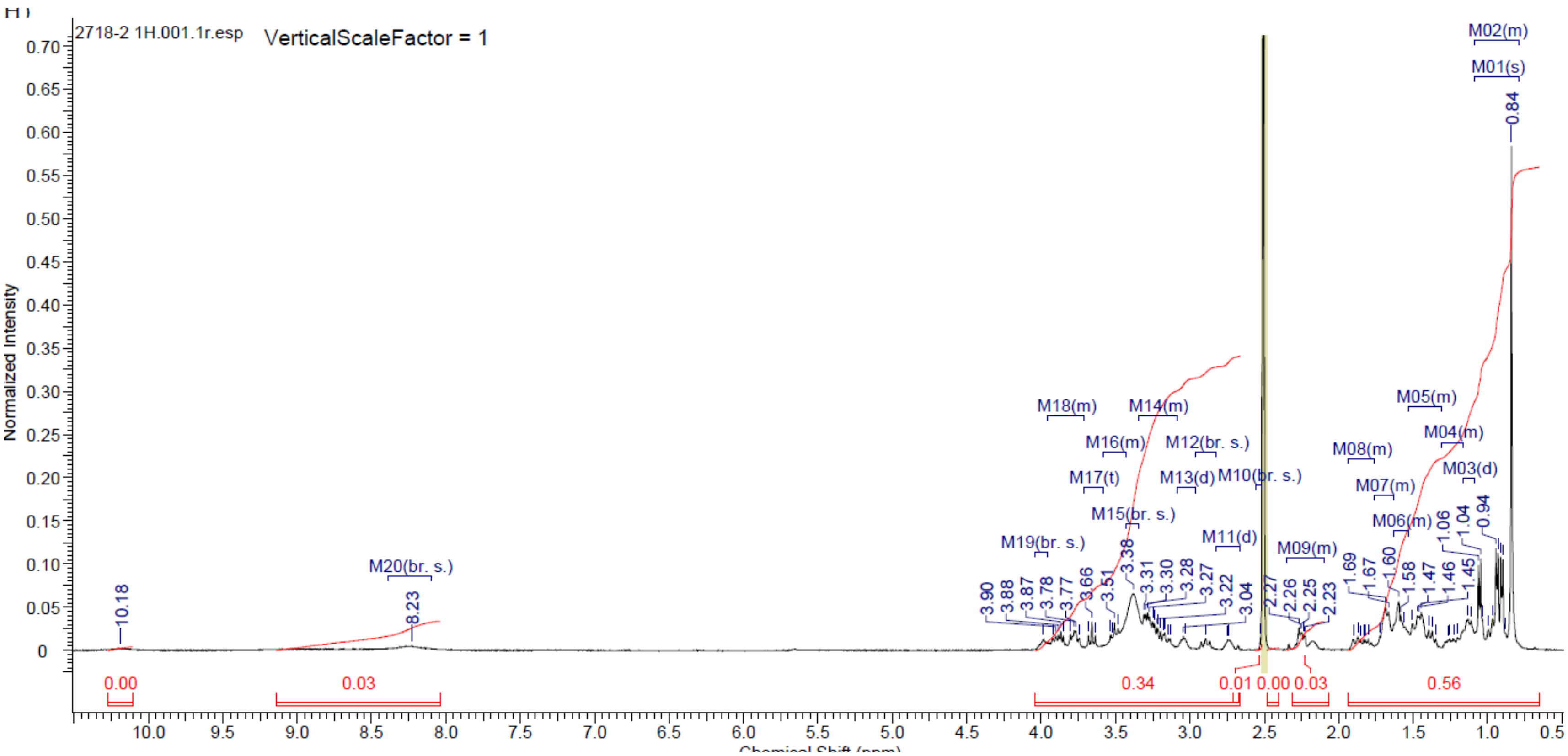

2718-2 1H.001.1r.esp
VerticalScaleFactor = 1
Normalized Intensity
M01(s)
M02(m)
M03(d)
M04(m)
M05(m)
M06(m)
M07(m)
M08(m)
M09(m)
M10(br. s.)
M11(d)
M12(br. s.)
M13(d)
M14(m)
M15(br. s.)
M16(m)
M17(t)
M18(m)
M19(br. s.)
M20(br. s.)
0.00
0.03
0.34
0.01
0.00
0.03
0.56

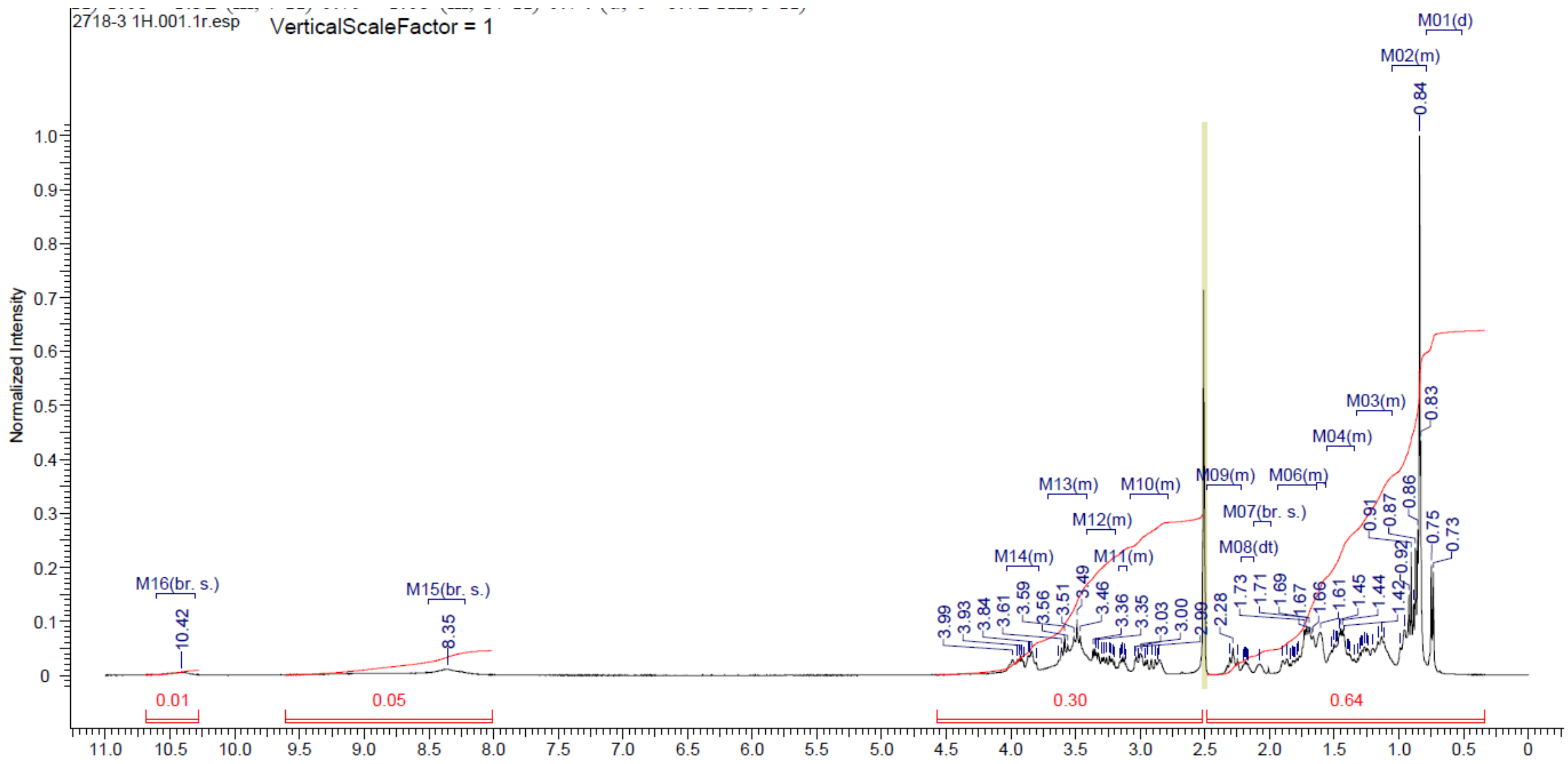
2718-3 1H.001.1r.esp
VerticalScaleFactor = 1
Normalized Intensity
M01(d)
M02(m)
M03(m)
M04(m)
M06(m)
M07(br. s.)
M08(dt)
M09(m)
M10(m)
M11(m)
M12(m)
M13(m)
M14(m)
M15(br. s.)
M16(br. s.)
10.42
8.35
3.99
3.93
3.84
3.61
3.59
3.56
3.51
3.49
3.46
3.36
3.35
3.03
3.00
2.99
2.28
1.73
1.71
1.69
1.67
1.66
1.61
1.45
1.44
1.42
0.92
0.91
0.87
0.86
0.84
0.83
0.75
0.73
0.01
0.05
0.30
0.64

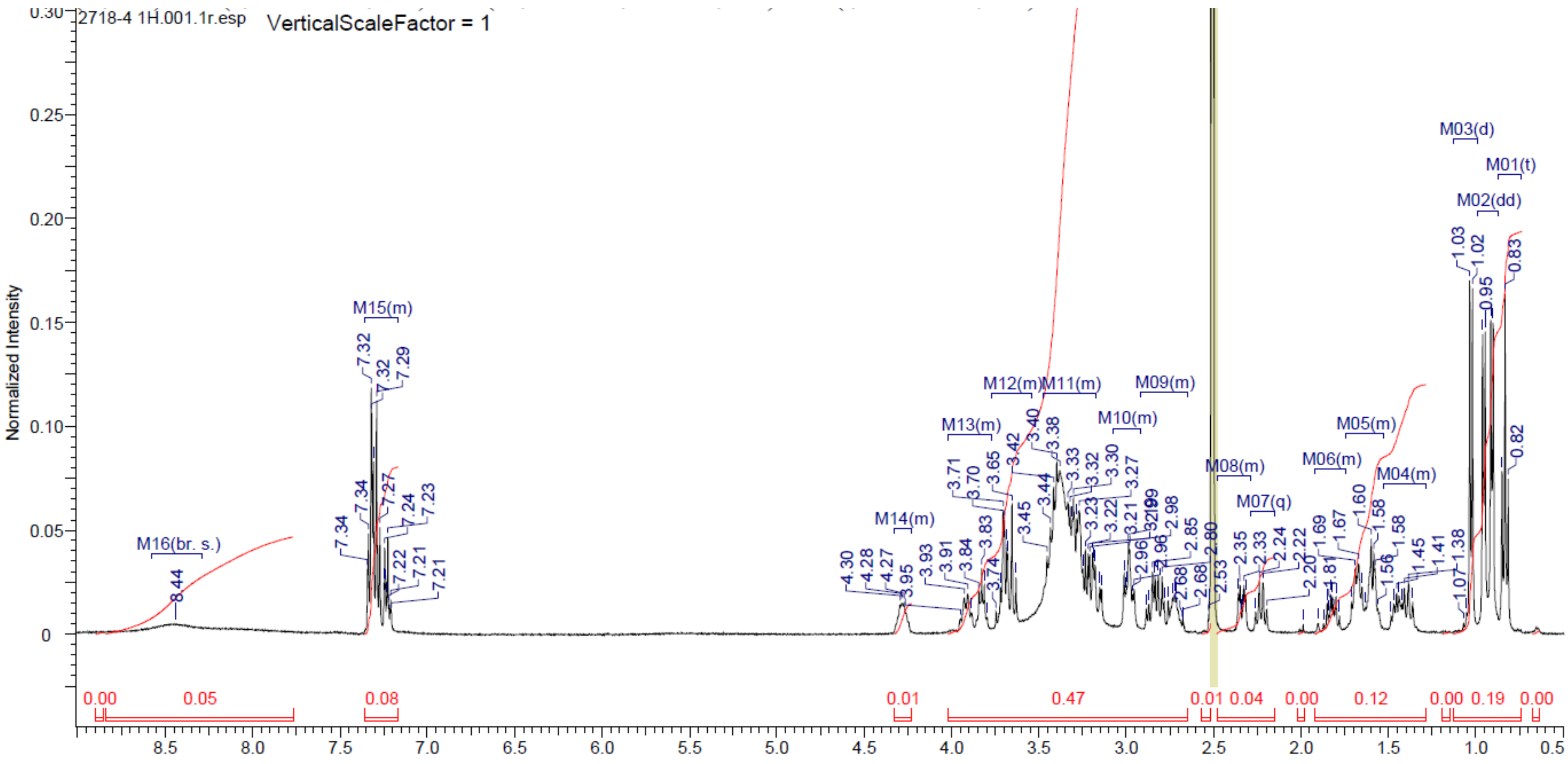
2718-4 1H.001.1r.esp
VerticalScaleFactor = 1
Normalized Intensity
M16(br. s.)
M15(m)
M14(m)
M13(m)
M12(m)
M11(m)
M10(m)
M09(m)
M08(m)
M07(q)
M06(m)
M05(m)
M04(m)
M03(d)
M02(dd)
M01(t)
0.00
0.05
0.08
0.01
0.47
0.01
0.04
0.00
0.12
0.00
0.19
0.00

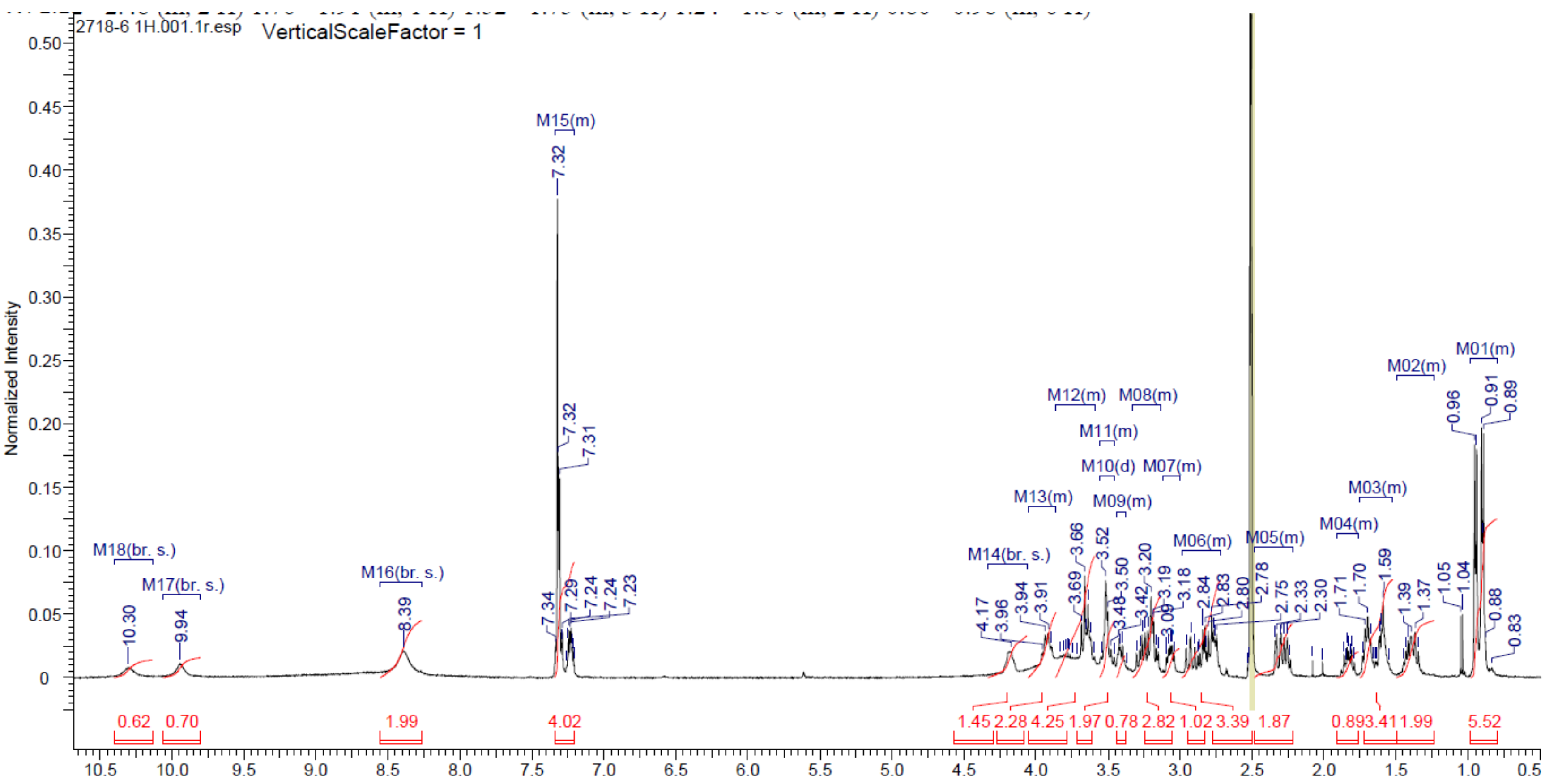
2718-6 1H.001.1r.esp
VerticalScaleFactor = 1
Normalized Intensity
M15(m)
M18(br. s.)
M17(br. s.)
M16(br. s.)
M14(br. s.)
M13(m)
M12(m)
M08(m)
M11(m)
M10(d)
M07(m)
M09(m)
M06(m)
M05(m)
M04(m)
M03(m)
M02(m)
M01(m)
0.62
0.70
1.99
4.02
1.45
2.28
4.25
1.97
0.78
2.82
1.02
3.39
1.87
0.89
3.41
1.99
5.52

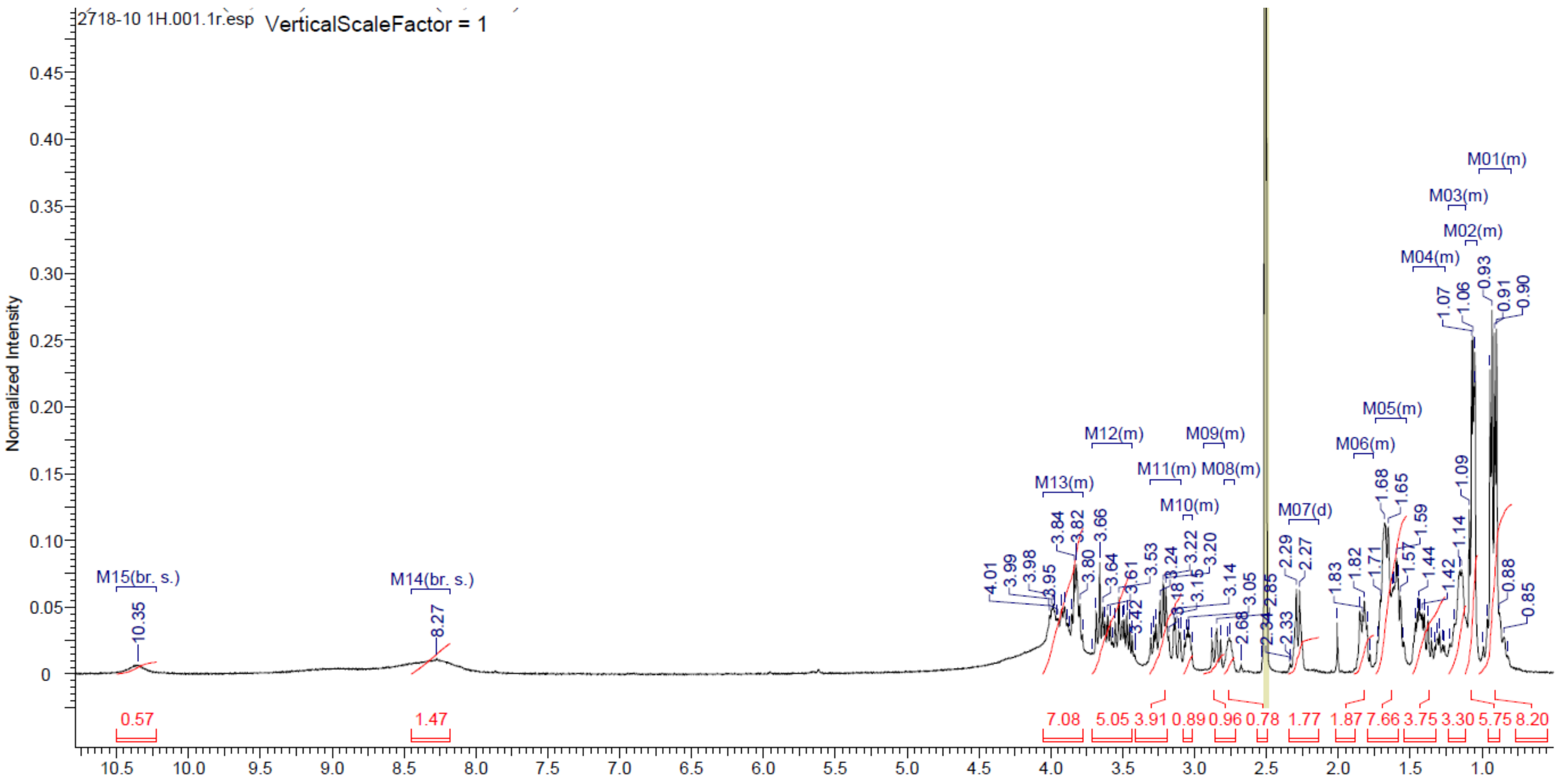

2718-10 1H.001.1r.esp
VerticalScaleFactor = 1
Normalized Intensity
M15(br. s.)
M14(br. s.)
M13(m)
M12(m)
M11(m)
M10(m)
M09(m)
M08(m)
M07(d)
M06(m)
M05(m)
M04(m)
M03(m)
M02(m)
M01(m)
0.57
1.47
7.08
5.05
3.91
0.89
0.96
0.78
1.77
1.87
7.66
3.75
3.30
5.75
8.20

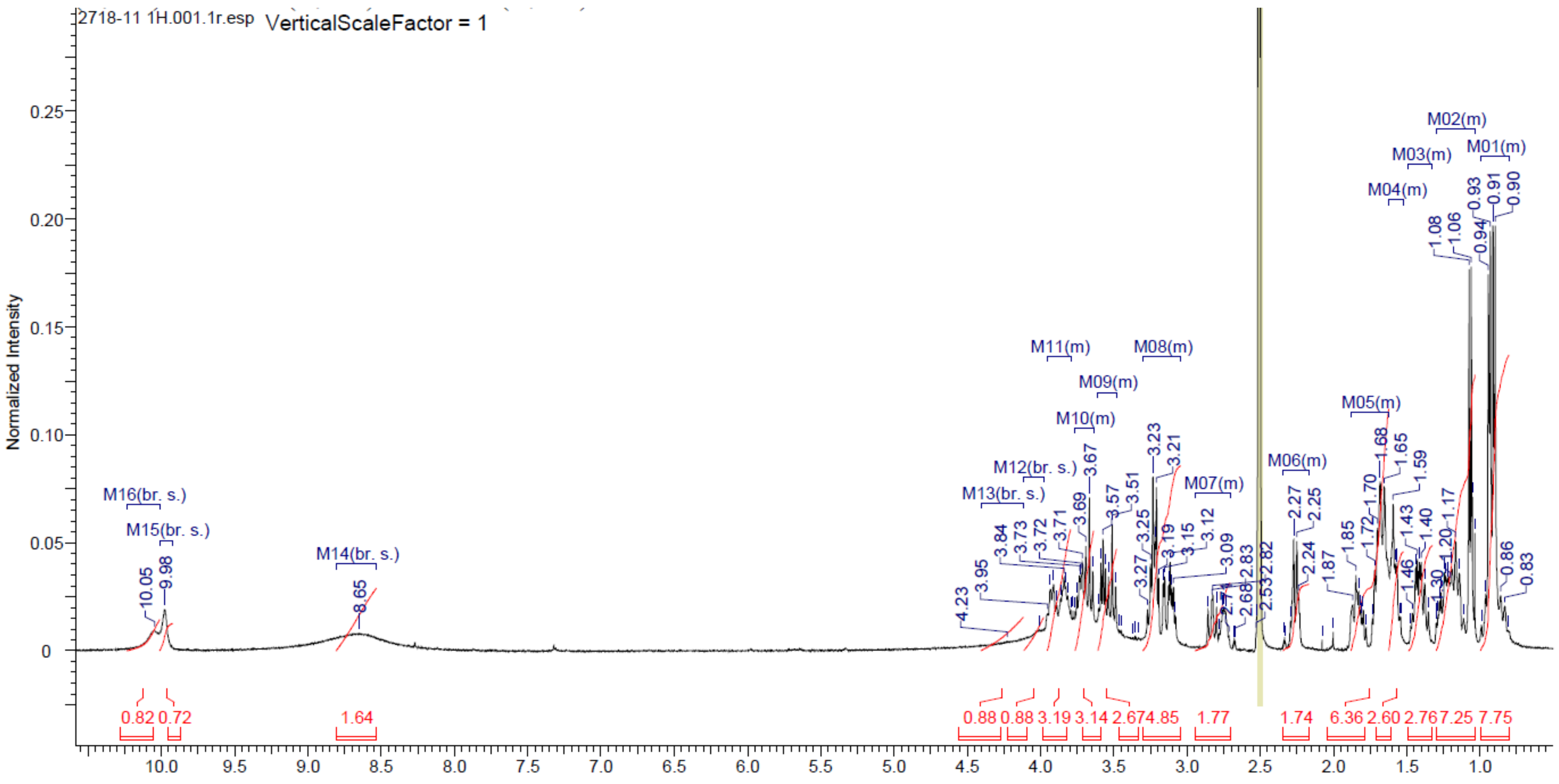
2718-11 1H.001.1r.esp
VerticalScaleFactor = 1
Normalized Intensity
M01(m)
M02(m)
M03(m)
M04(m)
M05(m)
M06(m)
M07(m)
M08(m)
M09(m)
M10(m)
M11(m)
M12(br. s.)
M13(br. s.)
M14(br. s.)
M15(br. s.)
M16(br. s.)
0.82 0.72 1.64 0.88 0.88 3.19 3.14 2.67 4.85 1.77 1.74 6.36 2.60 2.76 7.25 7.75

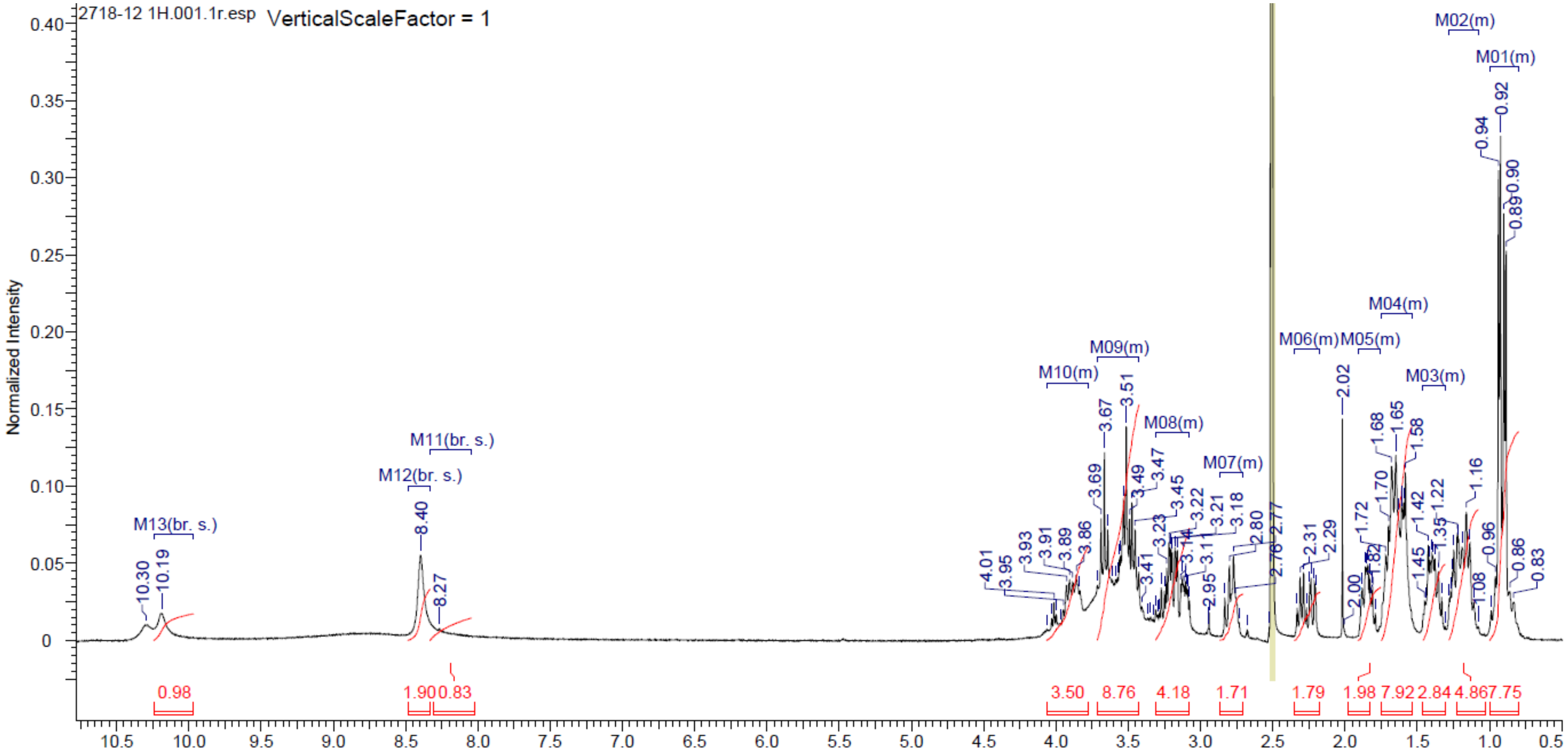

2718-12 1H.001.1r.esp
VerticalScaleFactor = 1
Normalized Intensity
M13(br. s.)
M12(br. s.)
M11(br. s.)
M10(m)
M09(m)
M08(m)
M07(m)
M06(m)
M05(m)
M04(m)
M03(m)
M02(m)
M01(m)
0.98
1.90
0.83
3.50
8.76
4.18
1.71
1.79
1.98
7.92
2.84
4.86
7.75

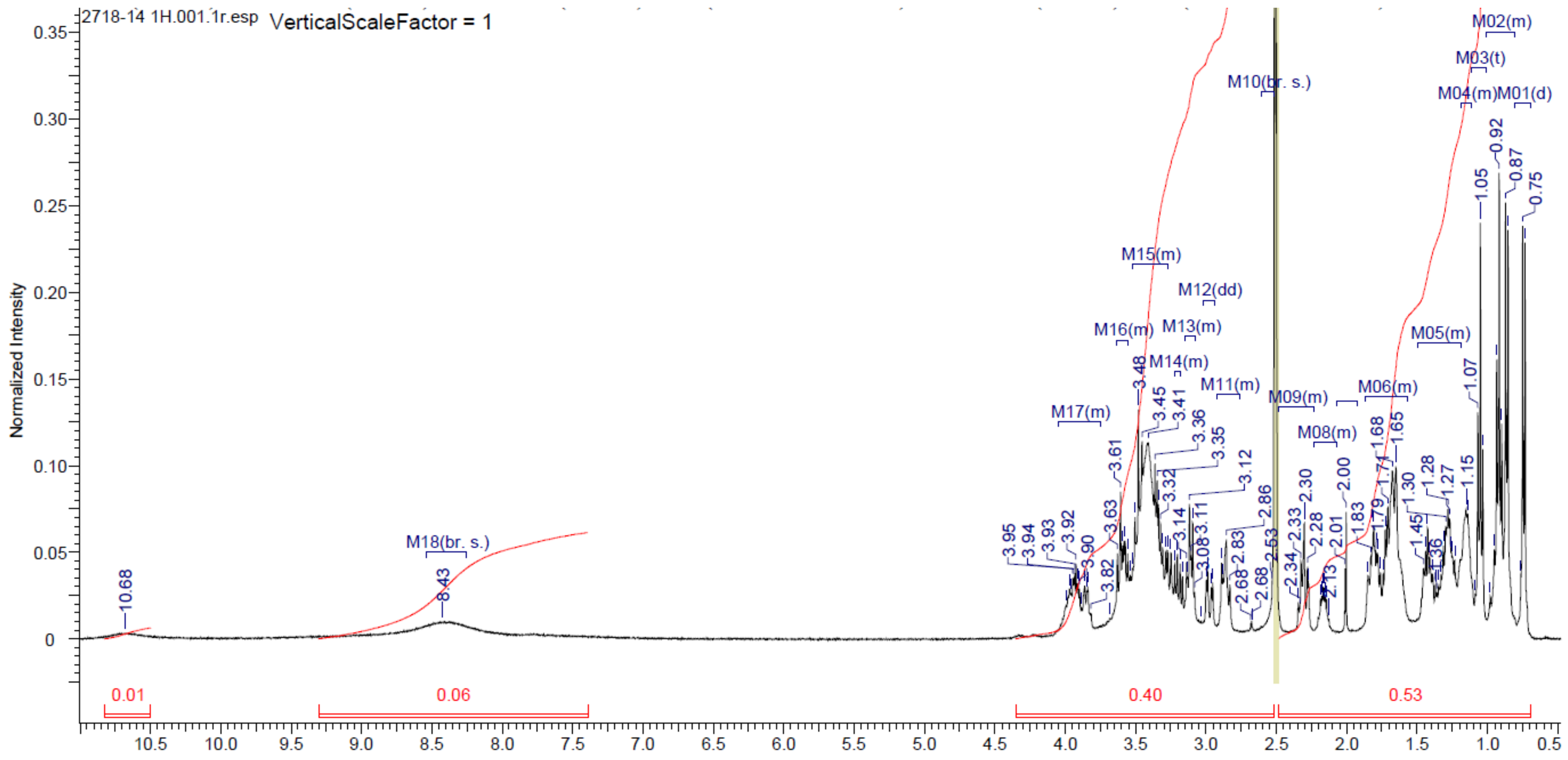

2718-14 1H.001.1r.esp
VerticalScaleFactor = 1
Normalized Intensity
M01(d)
M02(m)
M03(t)
M04(m)
M05(m)
M06(m)
M08(m)
M09(m)
M10(br. s.)
M11(m)
M12(dd)
M13(m)
M14(m)
M15(m)
M16(m)
M17(m)
M18(br. s.)
10.68
8.43
0.01
0.06
0.40
0.53

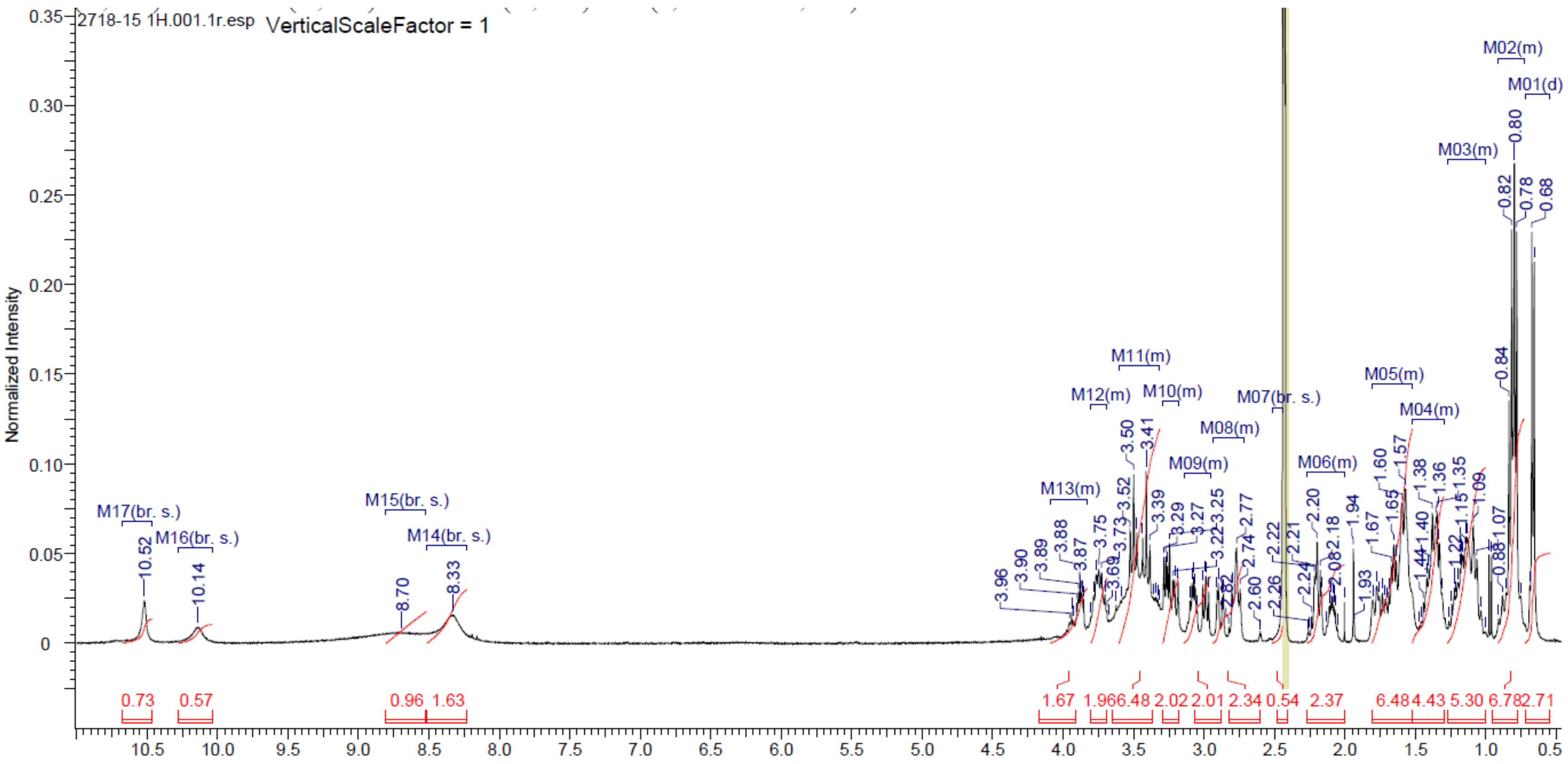

2718-15 1H.001.1r.esp
VerticalScaleFactor = 1
Normalized Intensity
M17(br. s.)
M16(br. s.)
M15(br. s.)
M14(br. s.)
M13(m)
M12(m)
M11(m)
M10(m)
M09(m)
M08(m)
M07(br. s.)
M06(m)
M05(m)
M04(m)
M03(m)
M02(m)
M01(d)
0.73
0.57
0.96
1.63
1.67
1.96
6.48
2.02
2.01
2.34
0.54
2.37
6.48
4.43
5.30
6.78
2.71

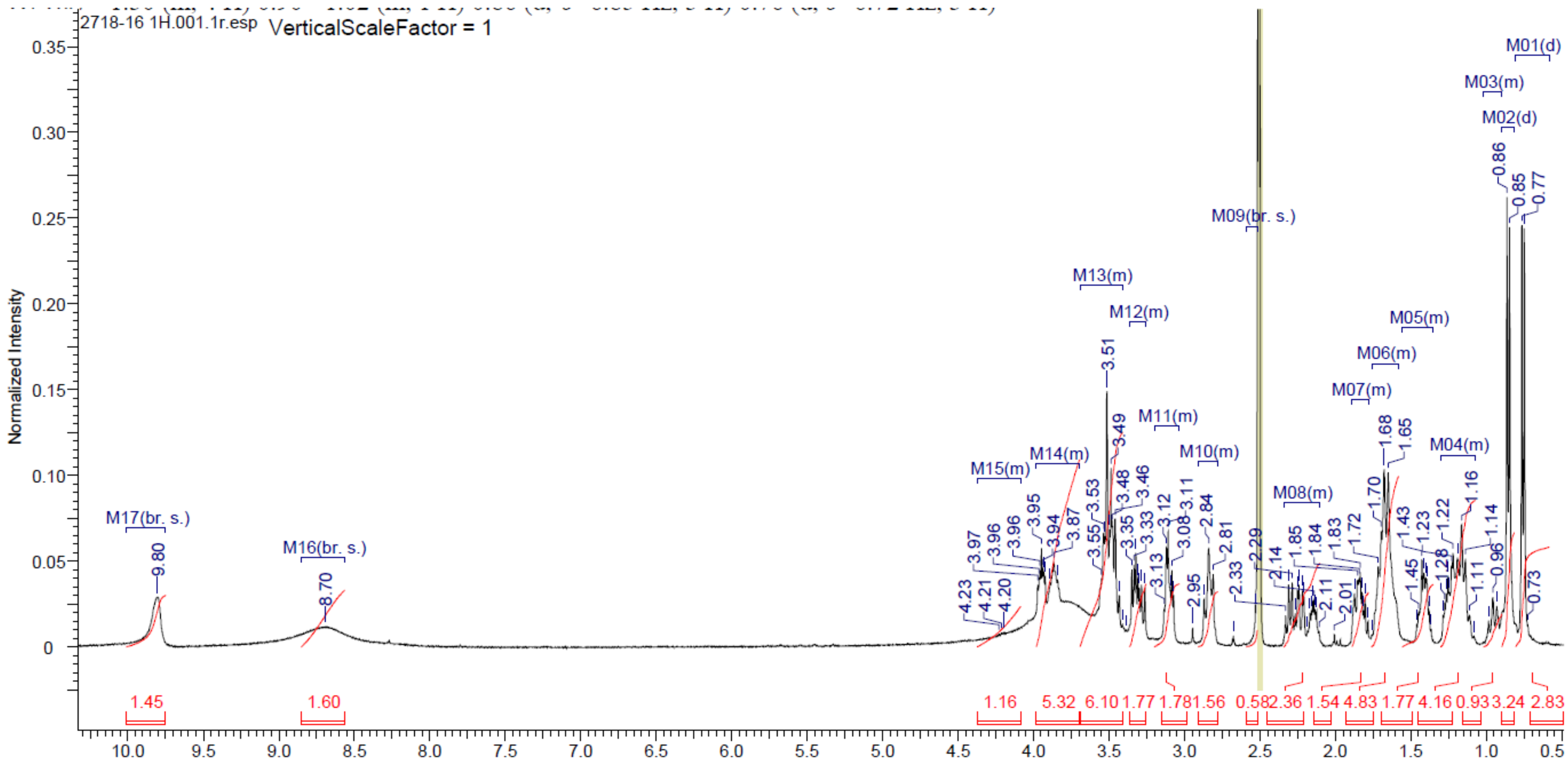
2718-16 1H.001.1r.esp
VerticalScaleFactor = 1
Normalized Intensity
M01(d)
M02(d)
M03(m)
M04(m)
M05(m)
M06(m)
M07(m)
M08(m)
M09(br. s.)
M10(m)
M11(m)
M12(m)
M13(m)
M14(m)
M15(m)
M16(br. s.)
M17(br. s.)
1.45
1.60
1.16
5.32
6.10
1.77
1.78
1.56
0.58
2.36
1.54
4.83
1.77
4.16
0.93
3.24
2.83

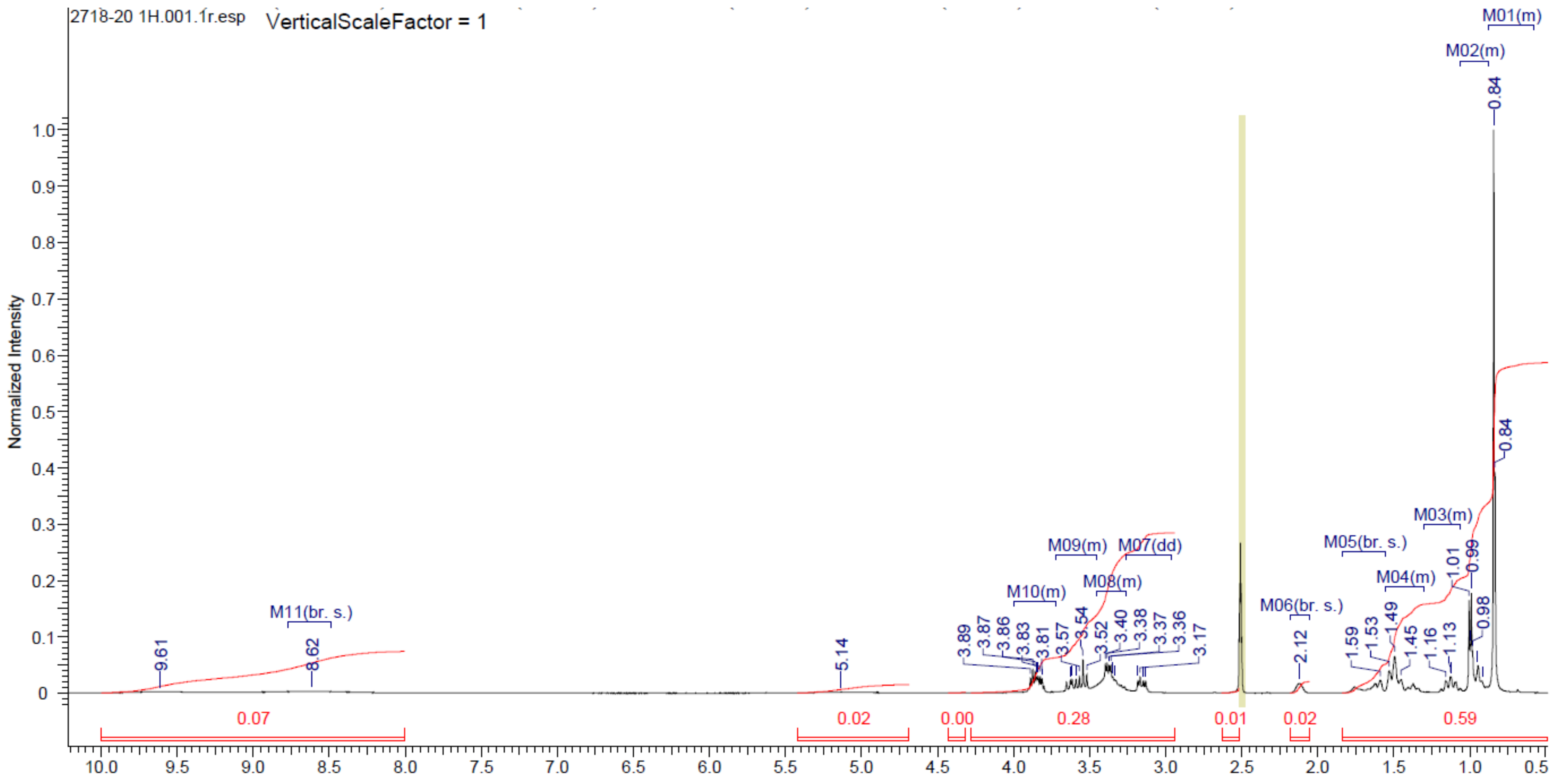

2718-20 1H.001.1r.esp
VerticalScaleFactor = 1
Normalized Intensity
M01(m)
M02(m)
M03(m)
M04(m)
M05(br. s.)
M06(br. s.)
M07(dd)
M08(m)
M09(m)
M10(m)
M11(br. s.)
0.07
0.02
0.00
0.28
0.01
0.02
0.59

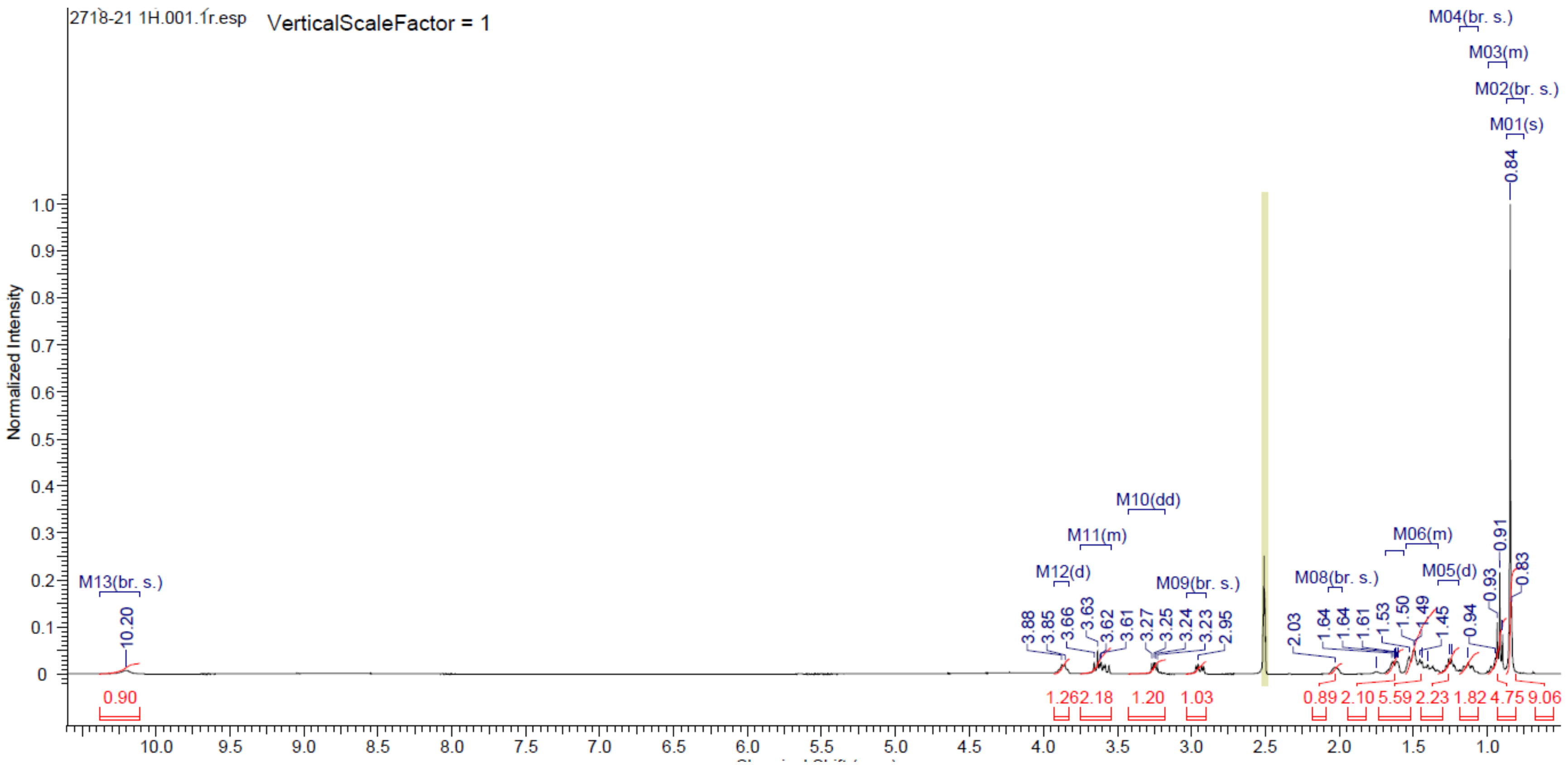

2718-21 1H.001.1r.esp
VerticalScaleFactor = 1
Normalized Intensity
M13(br. s.)
M12(d)
M11(m)
M10(dd)
M09(br. s.)
M08(br. s.)
M06(m)
M05(d)
M04(br. s.)
M03(m)
M02(br. s.)
M01(s)
10.20
3.88
3.85
3.66
3.63
3.62
3.61
3.27
3.25
3.24
3.23
2.95
2.03
1.64
1.64
1.61
1.53
1.50
1.49
1.45
0.94
0.93
0.91
0.84
0.83
0.90
1.26
2.18
1.20
1.03
0.89
2.10
5.59
2.23
1.82
4.75
9.06

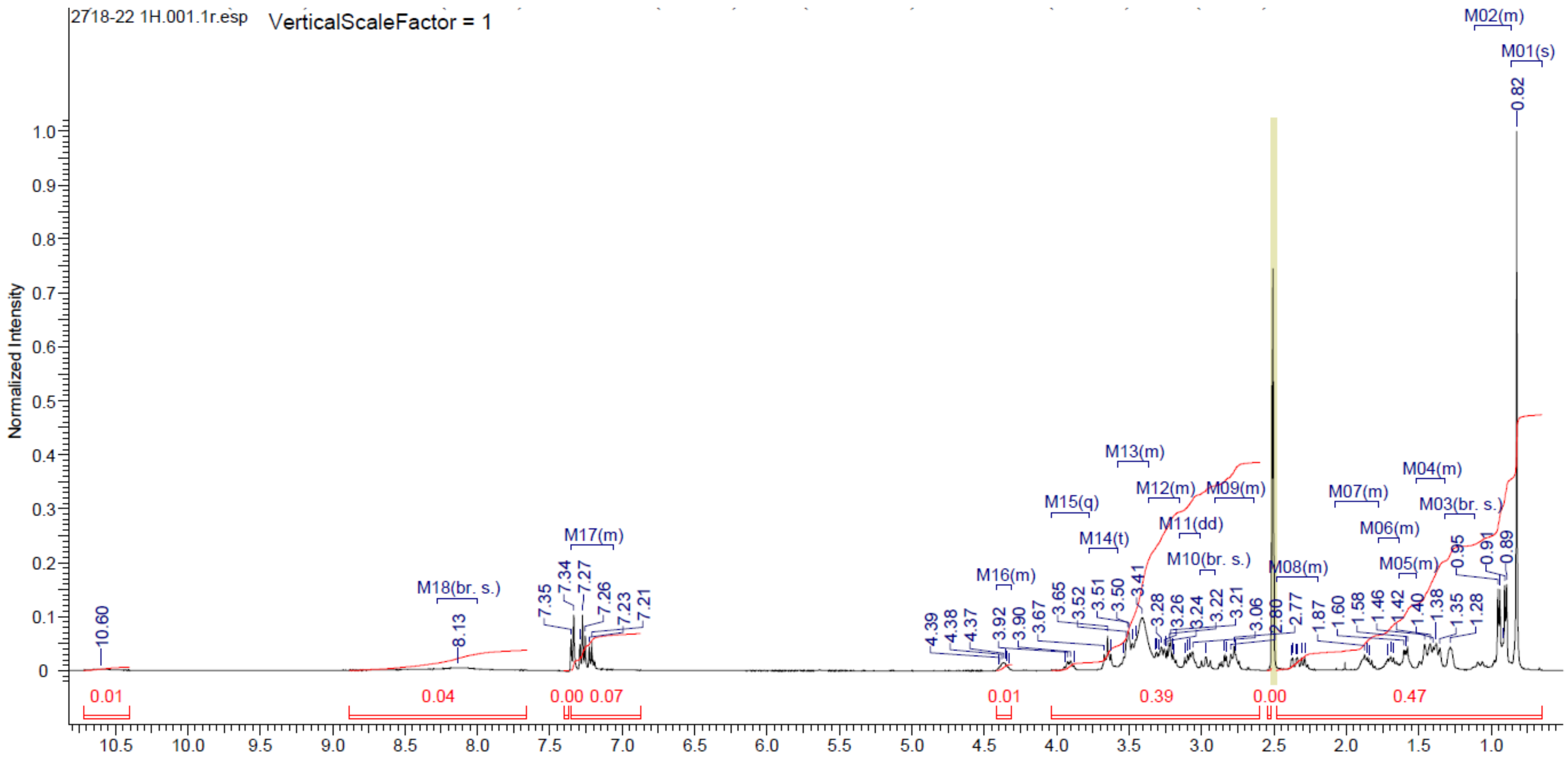

2718-22 1H.001.1r.esp
VerticalScaleFactor = 1
Normalized Intensity
M01(s)
M02(m)
M03(br. s.)
M04(m)
M05(m)
M06(m)
M07(m)
M08(m)
M09(m)
M10(br. s.)
M11(dd)
M12(m)
M13(m)
M14(t)
M15(q)
M16(m)
M17(m)
M18(br. s.)
0.01
0.04
0.00 0.07
0.01
0.39
0.00
0.47

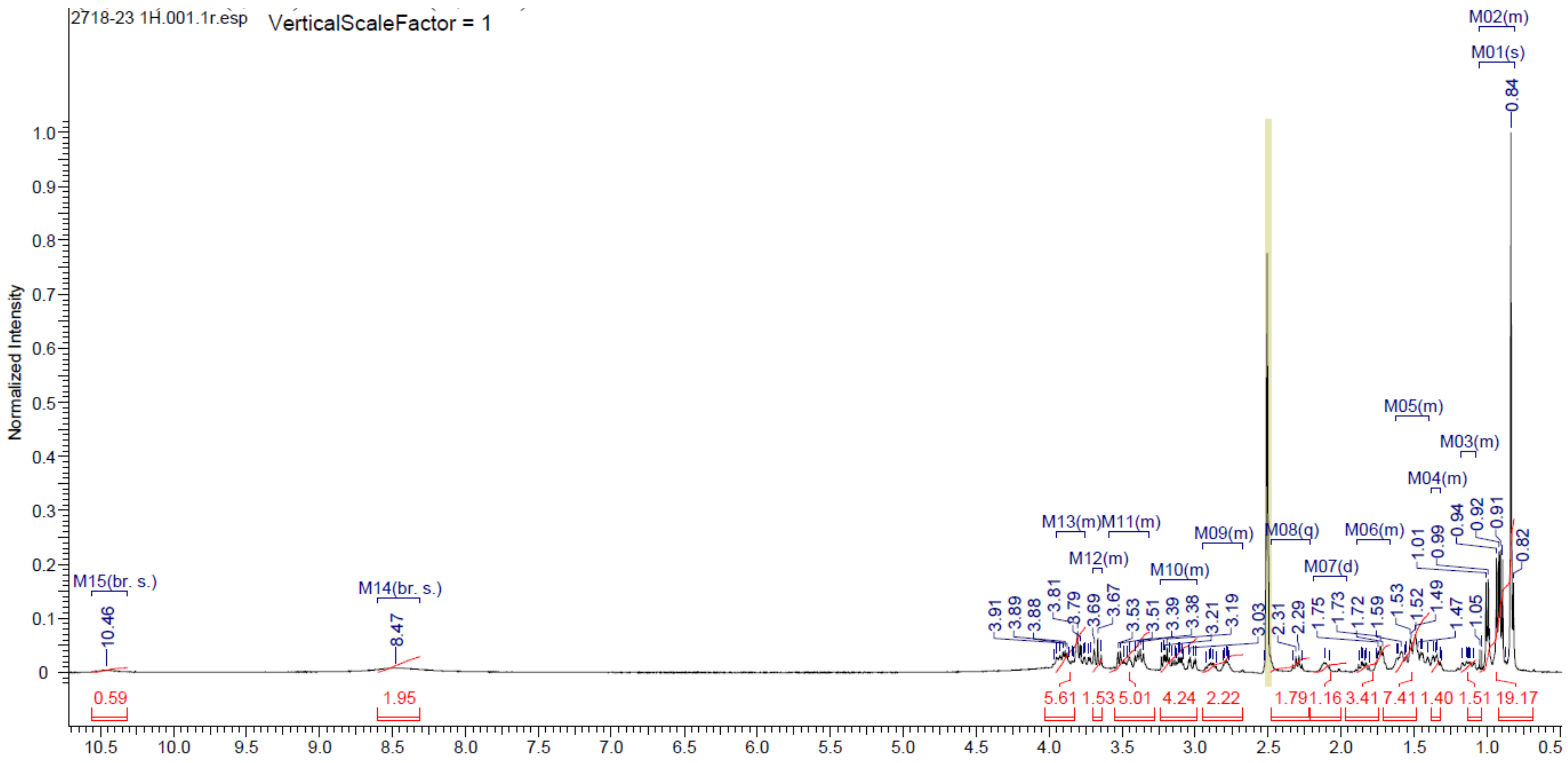

2718-23 1H.001.1r.esp
VerticalScaleFactor = 1
Normalized Intensity
M01(s)
M02(m)
M03(m)
M04(m)
M05(m)
M06(m)
M07(d)
M08(q)
M09(m)
M10(m)
M11(m)
M12(m)
M13(m)
M14(br. s.)
M15(br. s.)
10.46
8.47
3.91
3.89
3.88
3.81
3.79
3.69
3.67
3.53
3.51
3.39
3.38
3.21
3.19
3.03
2.31
2.29
1.75
1.73
1.72
1.59
1.53
1.52
1.49
1.47
1.05
1.01
0.99
0.94
0.92
0.91
0.84
0.82
0.59
1.95
5.61
1.53
5.01
4.24
2.22
1.79
1.16
3.41
7.41
1.40
1.51
19.17

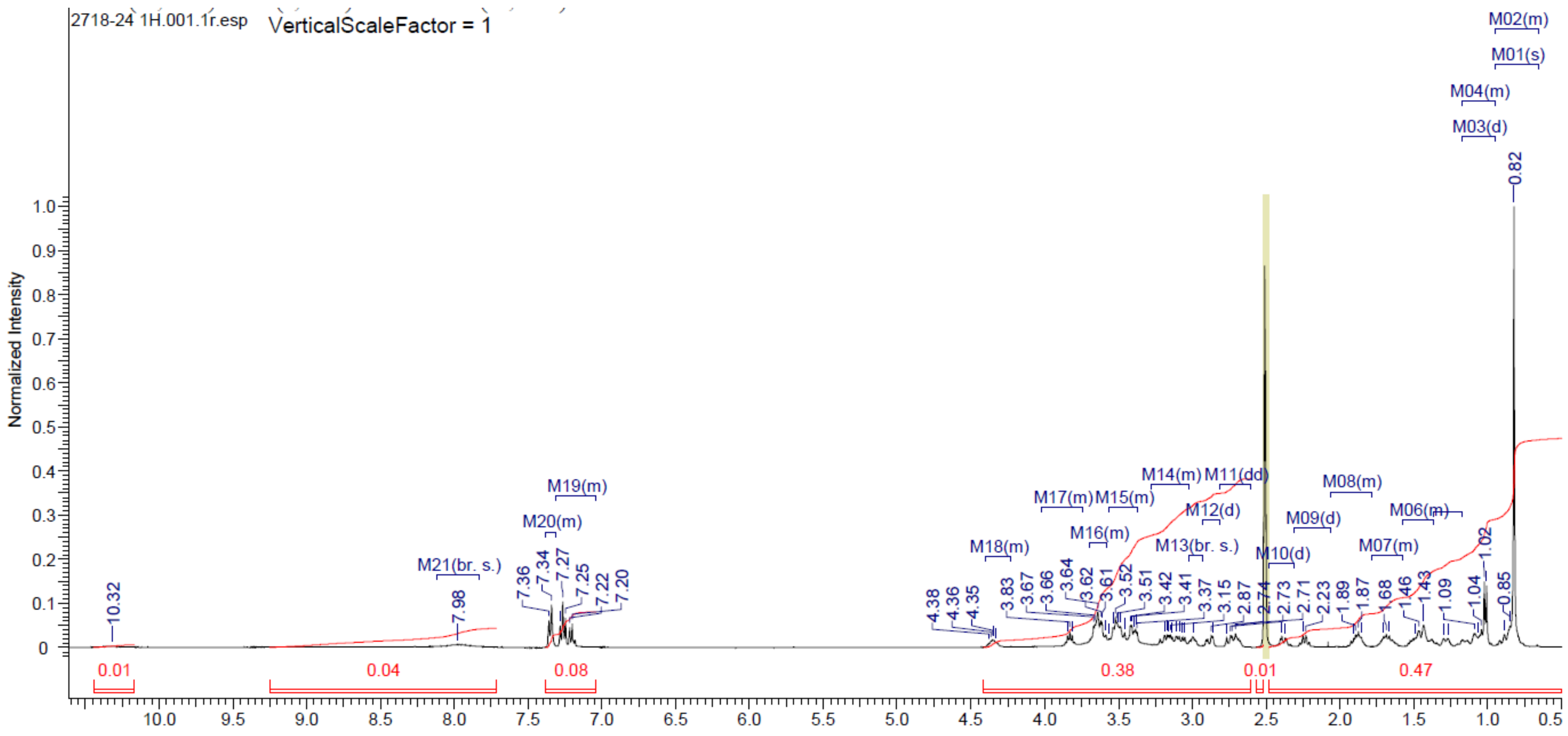

2718-24 1H.001.1r.esp
VerticalScaleFactor = 1
Normalized Intensity
M01(s)
M02(m)
M03(d)
M04(m)
M06(m)
M07(m)
M08(m)
M09(d)
M10(d)
M11(dd)
M12(d)
M13(br. s.)
M14(m)
M15(m)
M16(m)
M17(m)
M18(m)
M19(m)
M20(m)
M21(br. s.)
10.32
7.98
7.36
7.34
7.27
7.25
7.22
7.20
4.38
4.36
4.35
3.83
3.67
3.66
3.64
3.62
3.61
3.52
3.51
3.42
3.41
3.37
3.15
2.87
2.74
2.73
2.71
2.23
1.89
1.87
1.68
1.46
1.43
1.09
1.04
1.02
0.85
0.82
0.01
0.04
0.08
0.38
0.01
0.47

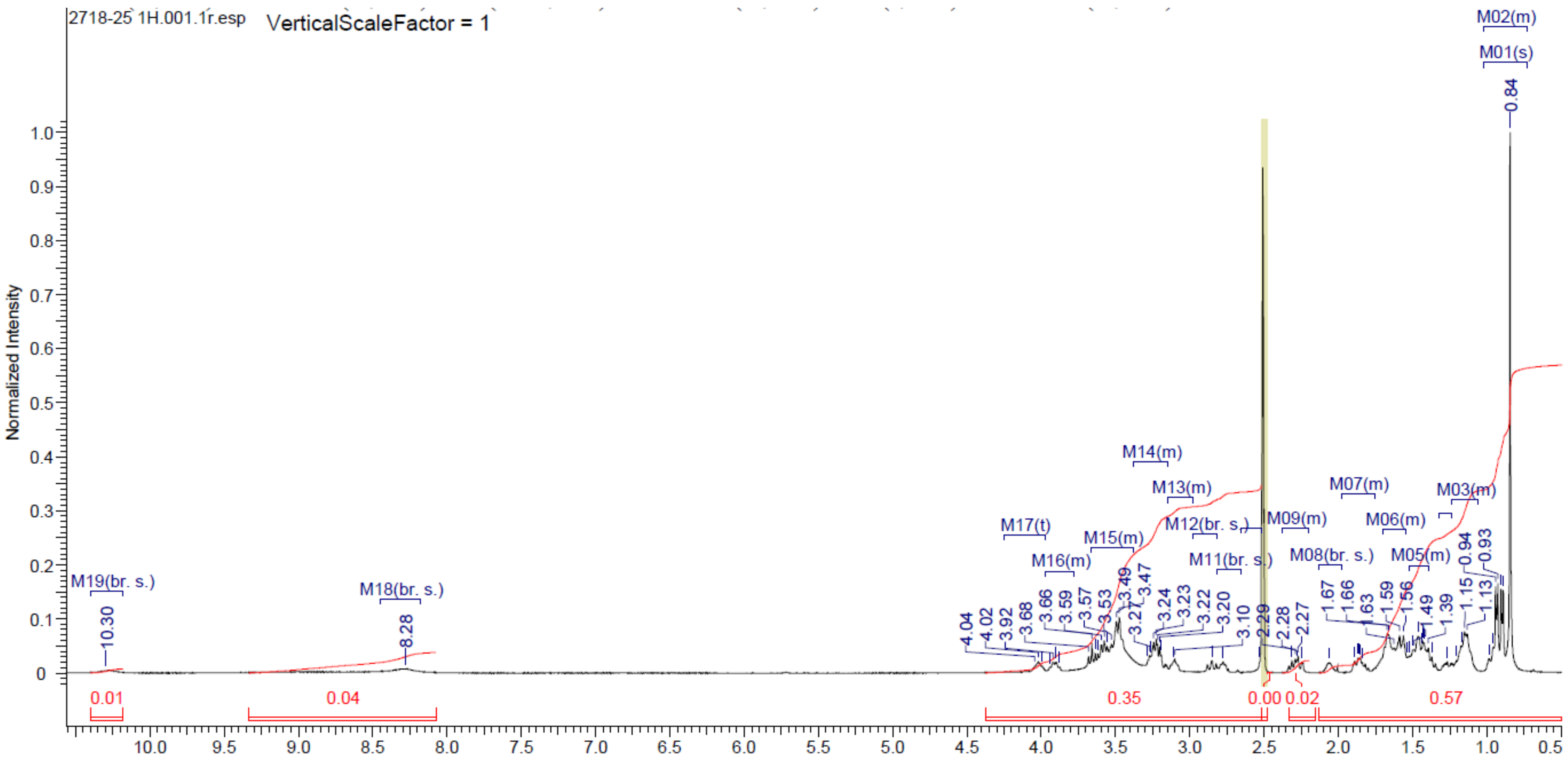

2718-25 1H.001.1r.esp
VerticalScaleFactor = 1
Normalized Intensity
M01(s)
M02(m)
M03(m)
M05(m)
M06(m)
M07(m)
M08(br. s.)
M09(m)
M11(br. s.)
M12(br. s.)
M13(m)
M14(m)
M15(m)
M16(m)
M17(t)
M18(br. s.)
M19(br. s.)
0.01
0.04
0.35
0.00
0.02
0.57

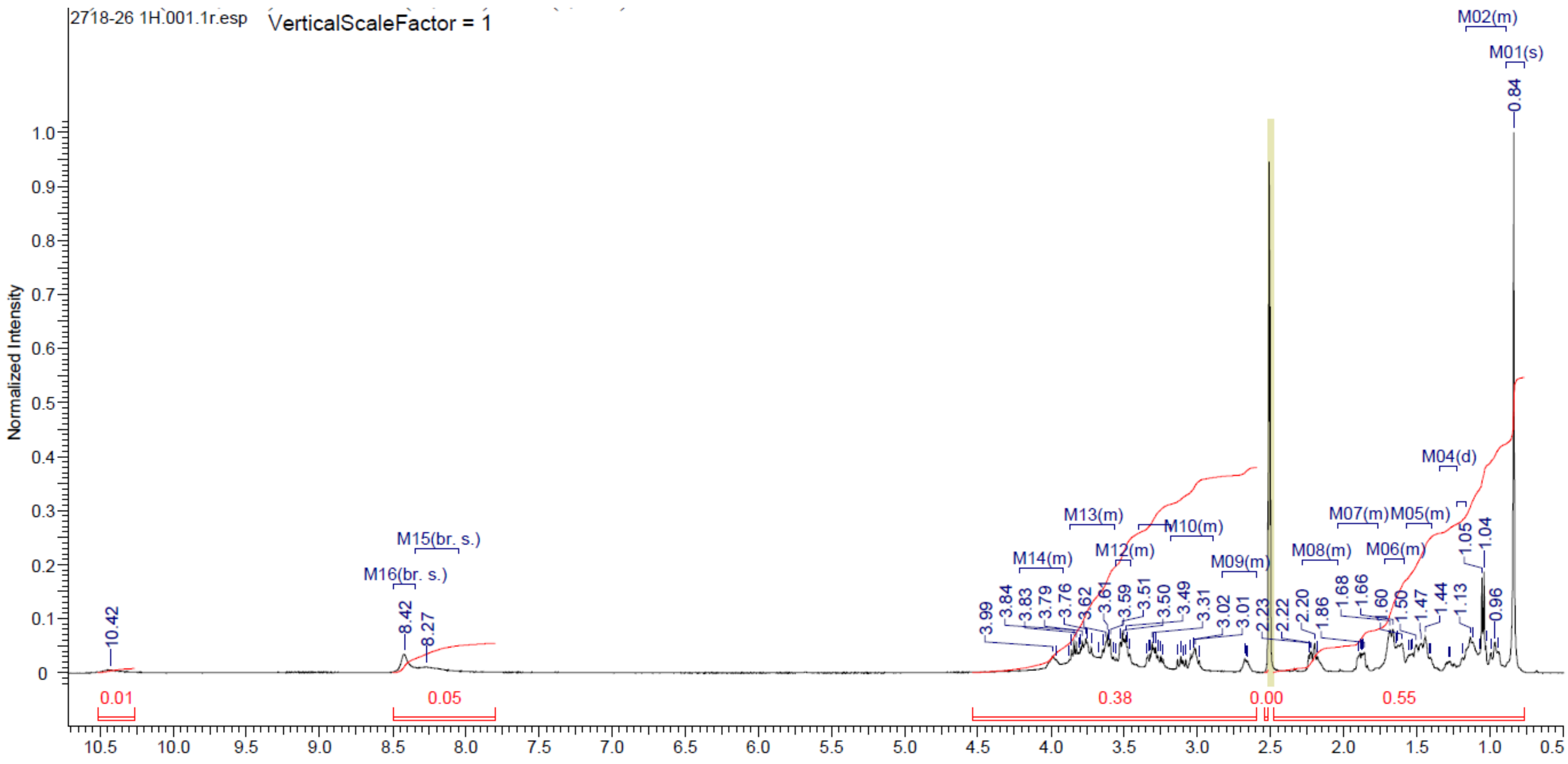

2718-26 1H.001.1r.esp
VerticalScaleFactor = 1
Normalized Intensity
M01(s)
M02(m)
M04(d)
M05(m)
M06(m)
M07(m)
M08(m)
M09(m)
M10(m)
M12(m)
M13(m)
M14(m)
M15(br. s.)
M16(br. s.)
0.01
0.05
0.38
0.00
0.55

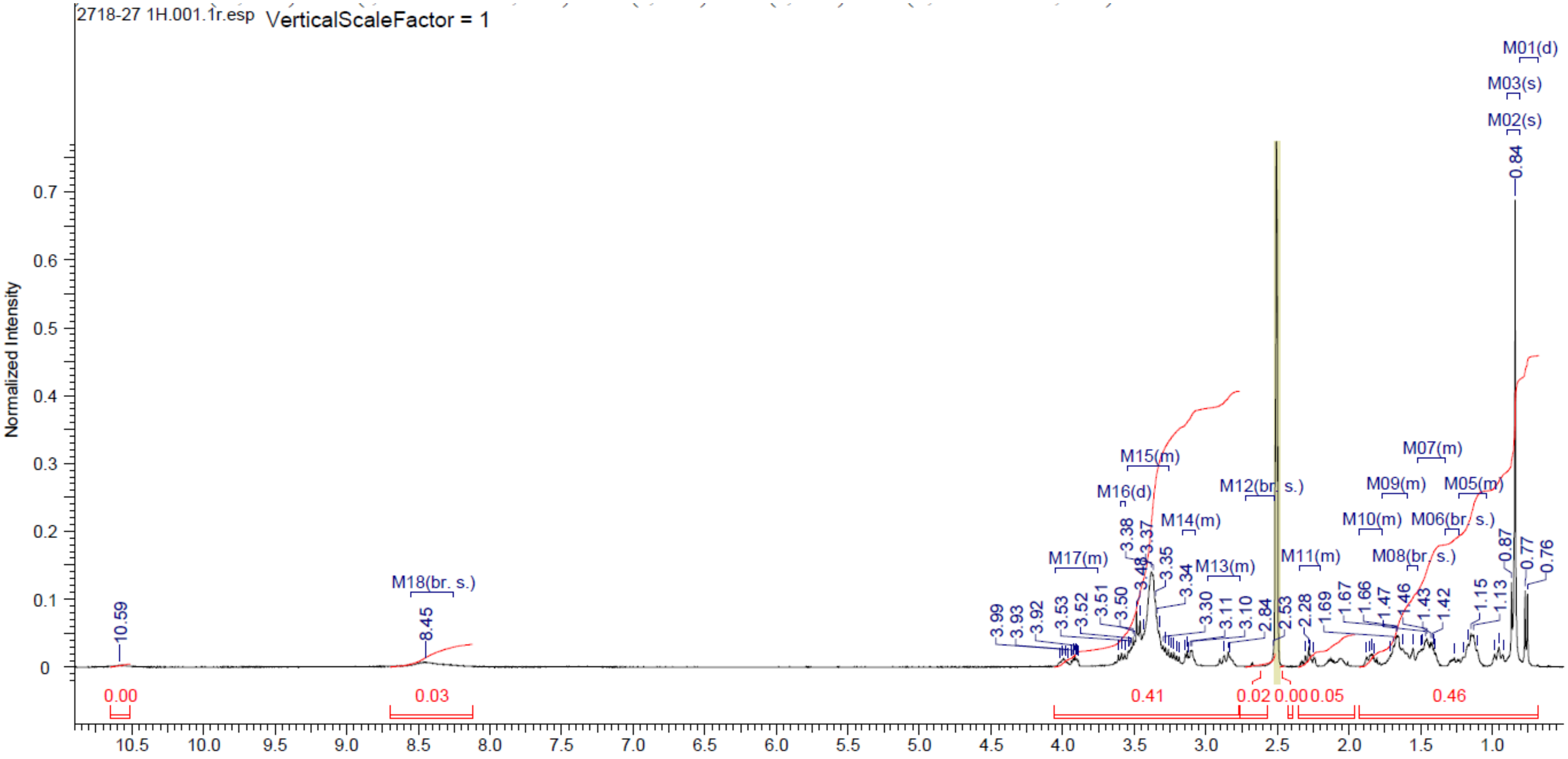
2718-27 1H.001.1r.esp
VerticalScaleFactor = 1
Normalized Intensity
M01(d)
M02(s)
M03(s)
M05(m)
M06(br. s.)
M07(m)
M08(br. s.)
M09(m)
M10(m)
M11(m)
M12(br. s.)
M13(m)
M14(m)
M15(m)
M16(d)
M17(m)
M18(br. s.)
0.00
0.03
0.41
0.02
0.00
0.05
0.46

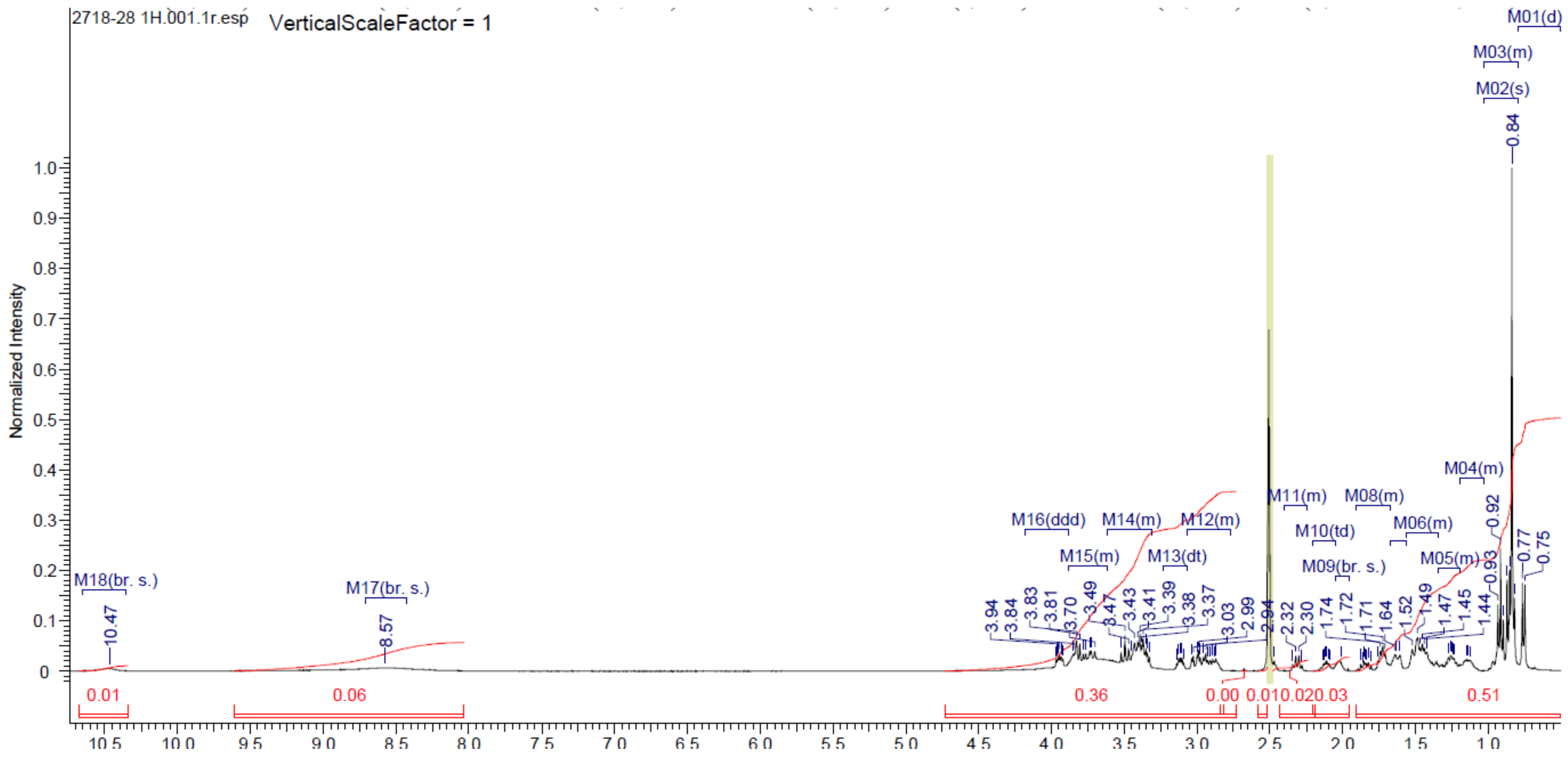
2718-28 1H.001.1r.esp
VerticalScaleFactor = 1
Normalized Intensity
M01(d)
M02(s)
M03(m)
M04(m)
M05(m)
M06(m)
M08(m)
M09(br. s.)
M10(td)
M11(m)
M12(m)
M13(dt)
M14(m)
M15(m)
M16(ddd)
M17(br. s.)
M18(br. s.)
10.47
8.57
3.94
3.84
3.83
3.81
3.70
3.49
3.47
3.43
3.41
3.39
3.38
3.37
3.03
2.99
2.94
2.32
2.30
1.74
1.72
1.71
1.64
1.52
1.49
1.47
1.45
1.44
0.93
0.92
0.84
0.77
0.75
0.01
0.06
0.36
0.00
0.01
0.02
0.03
0.51

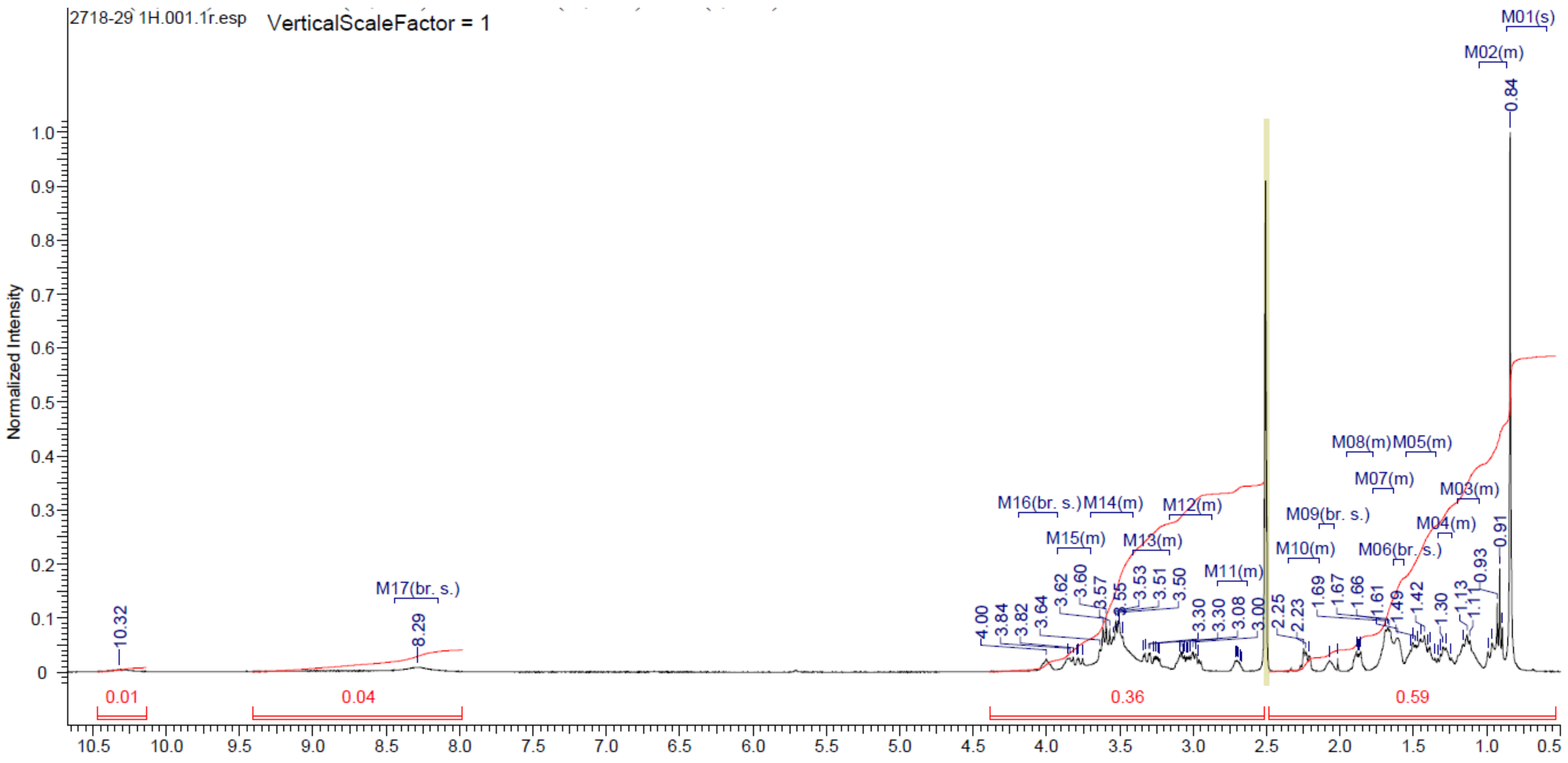
2718-29 1H.001.1r.esp
VerticalScaleFactor = 1
Normalized Intensity
M01(s)
M02(m)
M03(m)
M04(m)
M05(m)
M06(br. s.)
M07(m)
M08(m)
M09(br. s.)
M10(m)
M11(m)
M12(m)
M13(m)
M14(m)
M15(m)
M16(br. s.)
M17(br. s.)
0.01
0.04
0.36
0.59

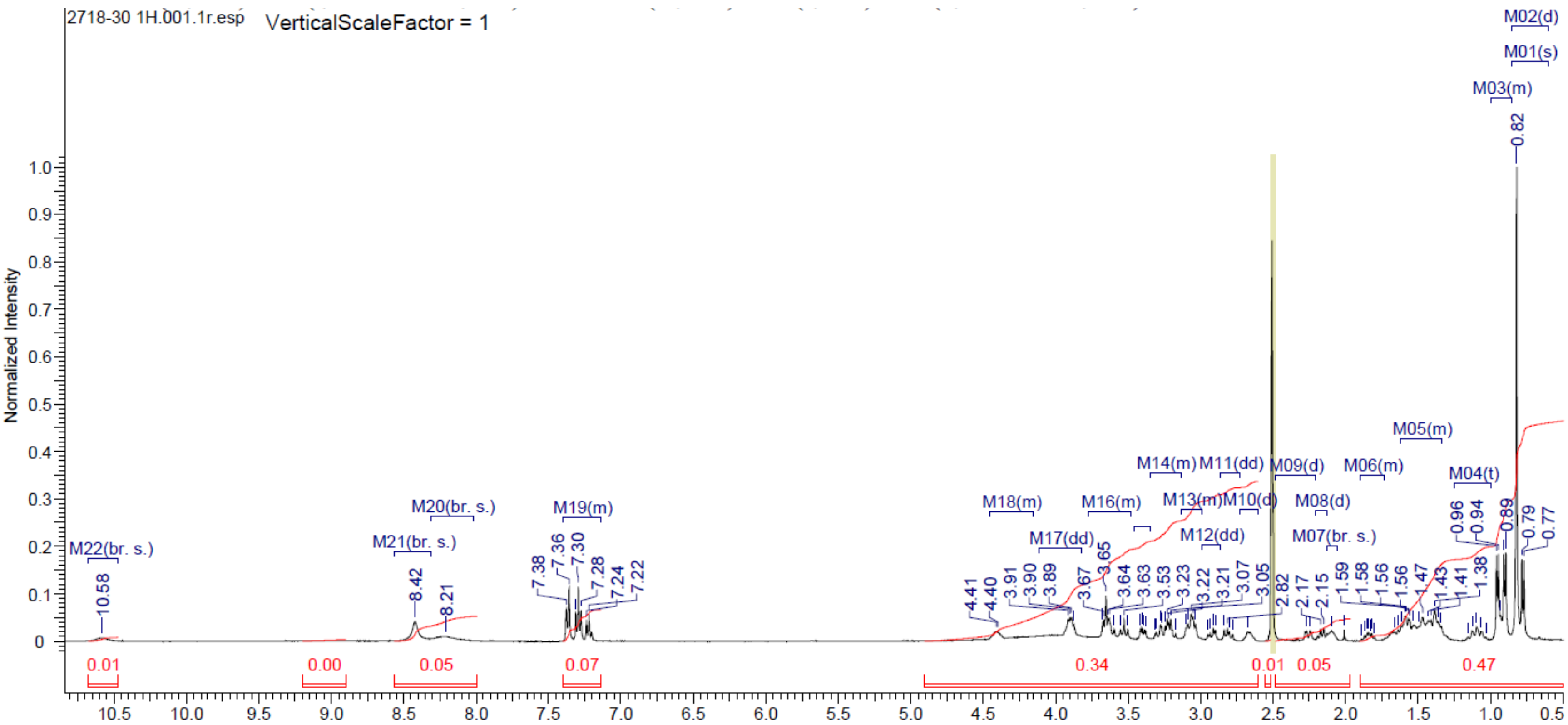

2718-30 1H.001.1r.esp
VerticalScaleFactor = 1
Normalized Intensity
M01(s)
M02(d)
M03(m)
M04(t)
M05(m)
M06(m)
M07(br. s.)
M08(d)
M09(d)
M10(d)
M11(dd)
M12(dd)
M13(m)
M14(m)
M16(m)
M17(dd)
M18(m)
M19(m)
M20(br. s.)
M21(br. s.)
M22(br. s.)
0.01
0.00
0.05
0.07
0.34
0.01
0.05
0.47

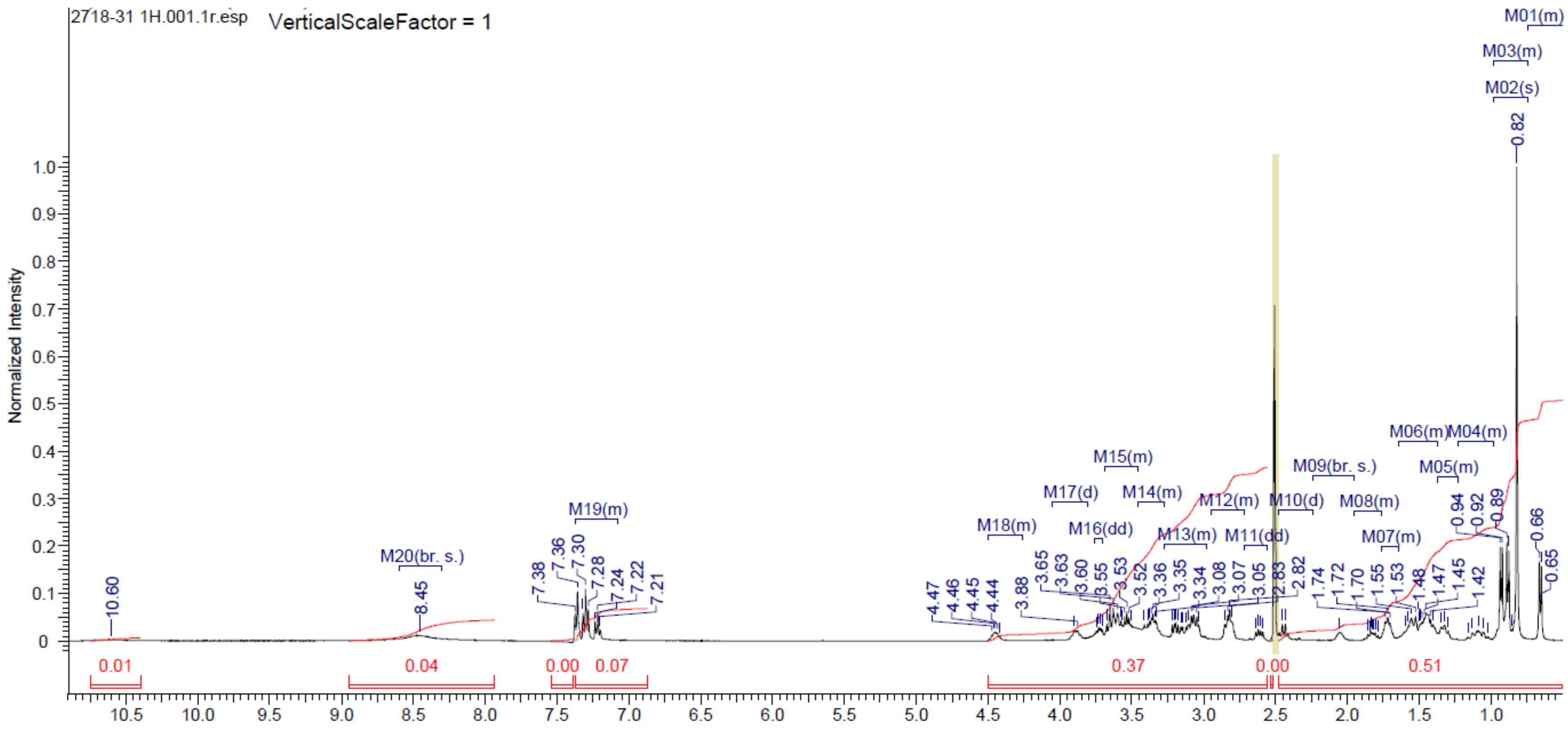
2718-31 1H.001.1r.esp
VerticalScaleFactor = 1
Normalized Intensity
M01(m)
M03(m)
M02(s)
M20(br. s.)
M19(m)
M18(m)
M17(d)
M16(dd)
M15(m)
M14(m)
M13(m)
M12(m)
M11(dd)
M10(d)
M09(br. s.)
M08(m)
M07(m)
M06(m)
M05(m)
M04(m)
10.60
8.45
7.38
7.36
7.30
7.28
7.24
7.22
7.21
4.47
4.46
4.45
4.44
3.88
3.65
3.63
3.60
3.55
3.53
3.52
3.36
3.35
3.34
3.08
3.07
3.05
2.83
2.82
1.74
1.72
1.70
1.55
1.53
1.48
1.47
1.45
1.42
0.94
0.92
0.89
0.82
0.66
0.65
0.01
0.04
0.00
0.07
0.37
0.00
0.51

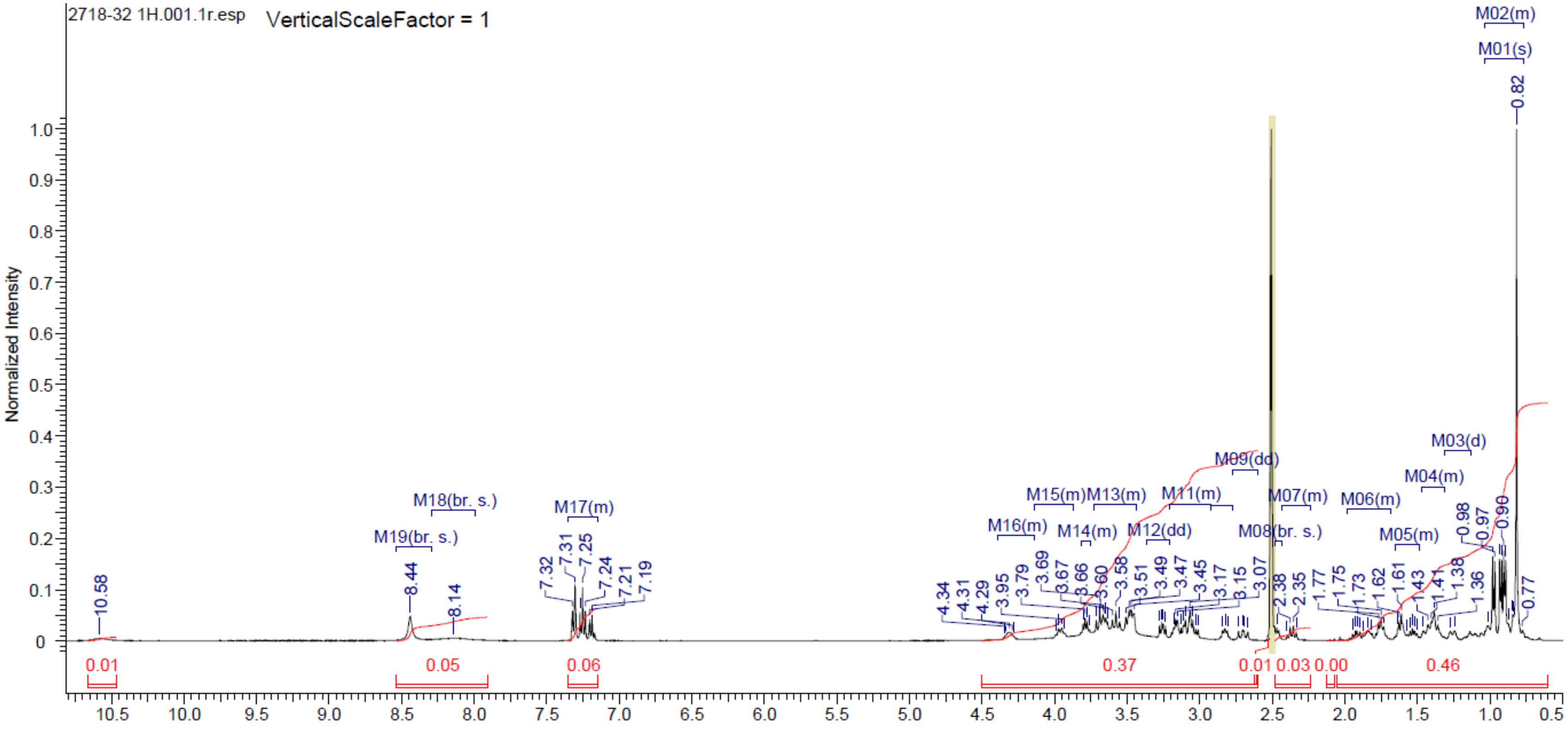

2718-32 1H.001.1r.esp
VerticalScaleFactor = 1
Normalized Intensity
M01(s)
M02(m)
M03(d)
M04(m)
M05(m)
M06(m)
M07(m)
M08(br. s.)
M09(dd)
M11(m)
M12(dd)
M13(m)
M14(m)
M15(m)
M16(m)
M17(m)
M18(br. s.)
M19(br. s.)
0.01
0.05
0.06
0.37
0.01
0.03
0.00
0.46

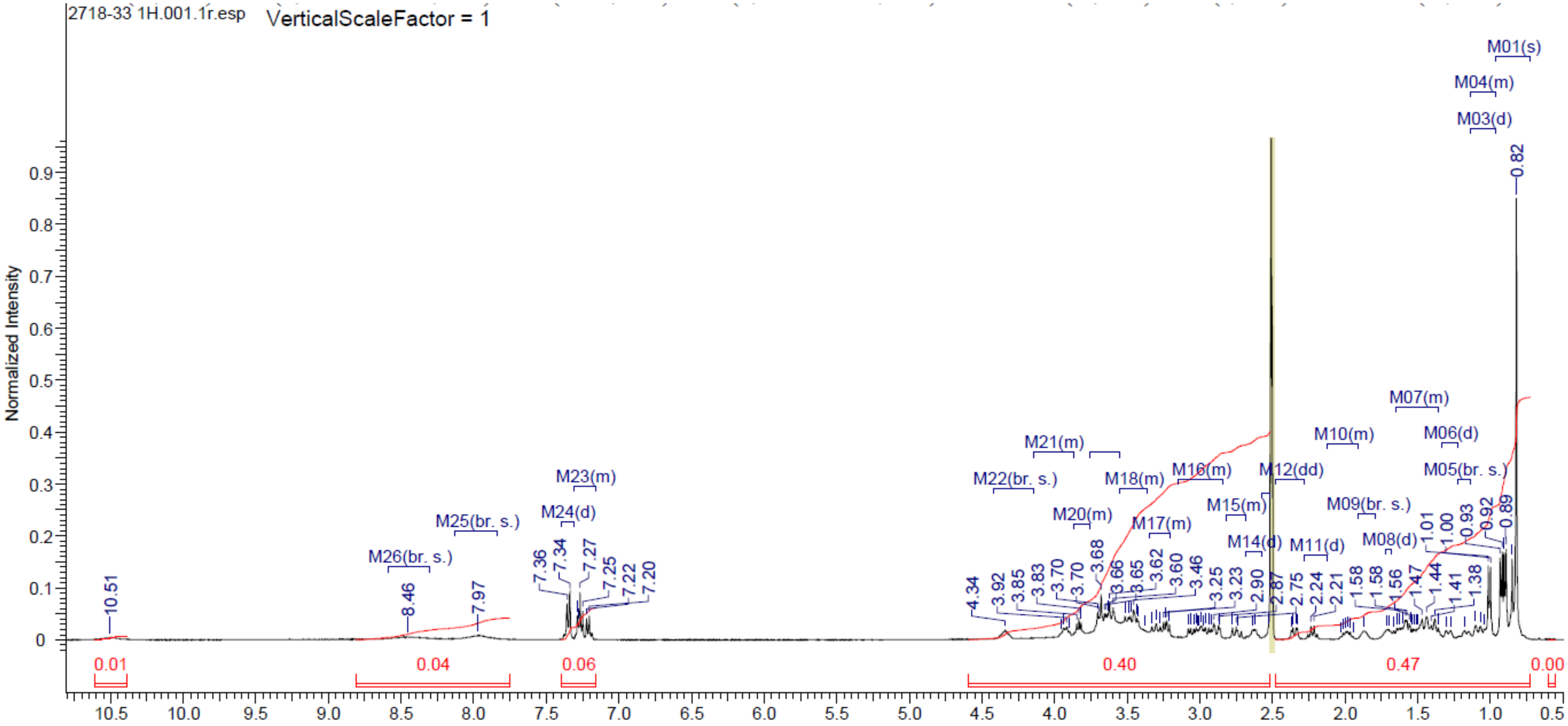

2718-33 1H.001.1r.esp
VerticalScaleFactor = 1
Normalized Intensity
M01(s)
M04(m)
M03(d)
M07(m)
M06(d)
M05(br. s.)
M10(m)
M09(br. s.)
M08(d)
M12(dd)
M11(d)
M14(d)
M15(m)
M16(m)
M17(m)
M18(m)
M20(m)
M21(m)
M22(br. s.)
M23(m)
M24(d)
M25(br. s.)
M26(br. s.)
10.51
8.46
7.97
7.36
7.34
7.27
7.25
7.22
7.20
4.34
3.92
3.85
3.83
3.70
3.70
3.68
3.66
3.65
3.62
3.60
3.46
3.25
3.23
2.90
2.87
2.75
2.24
2.21
1.58
1.58
1.56
1.47
1.44
1.41
1.38
1.01
1.00
0.93
0.92
0.89
0.82
0.01
0.04
0.06
0.40
0.47
0.00

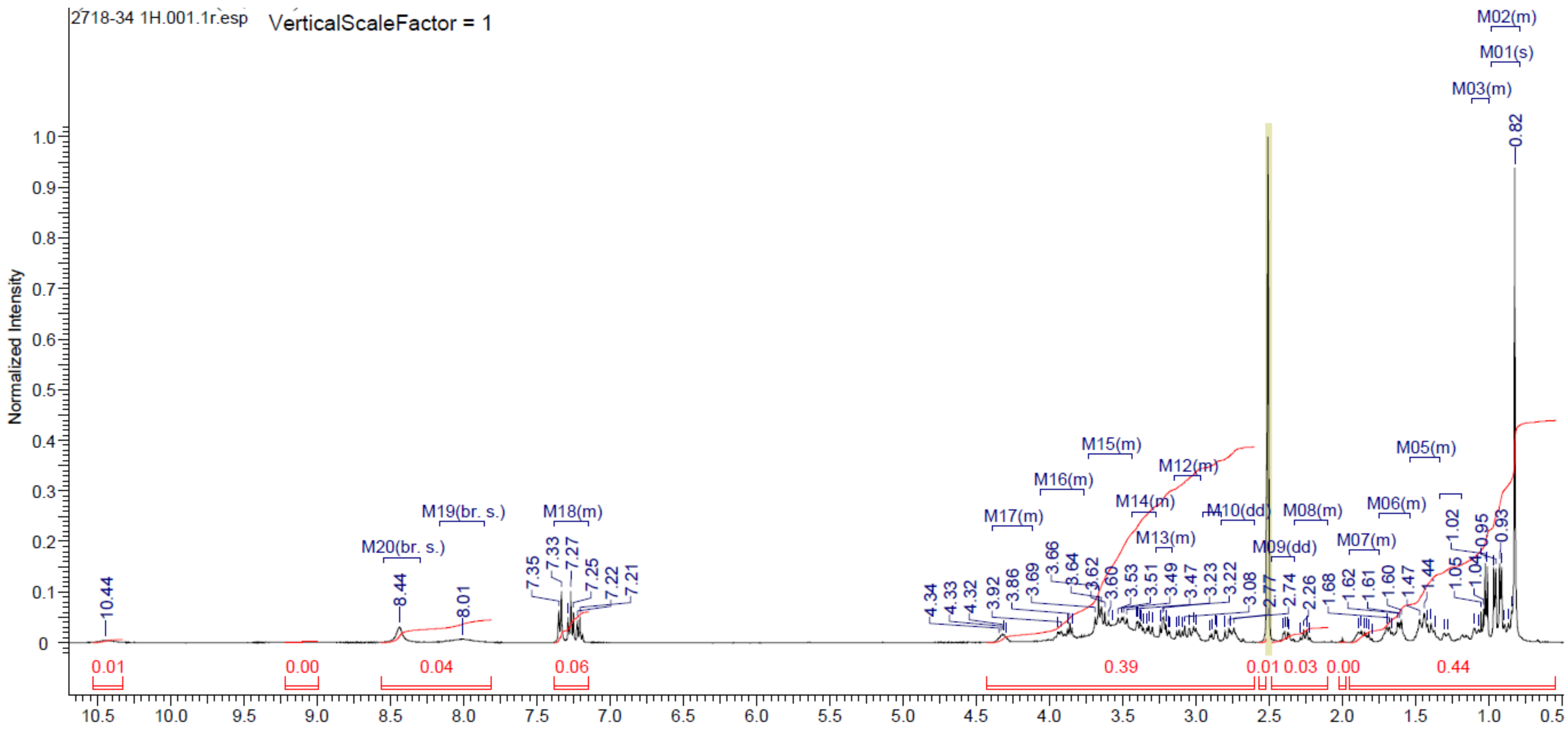

2718-34 1H.001.1r.esp
VerticalScaleFactor = 1
Normalized Intensity
M02(m)
M01(s)
M03(m)
M15(m)
M12(m)
M16(m)
M14(m)
M17(m)
M13(m)
M10(dd)
M08(m)
M09(dd)
M07(m)
M06(m)
M05(m)
M20(br. s.)
M19(br. s.)
M18(m)
10.44
8.44
8.01
7.35
7.33
7.27
7.25
7.22
7.21
4.34
4.33
4.32
3.92
3.86
3.69
3.66
3.64
3.62
3.60
3.53
3.51
3.49
3.47
3.23
3.22
3.08
2.77
2.74
2.26
1.68
1.62
1.61
1.60
1.47
1.44
1.05
1.04
1.02
0.95
0.93
0.82
0.01
0.00
0.04
0.06
0.39
0.01
0.03
0.00
0.44

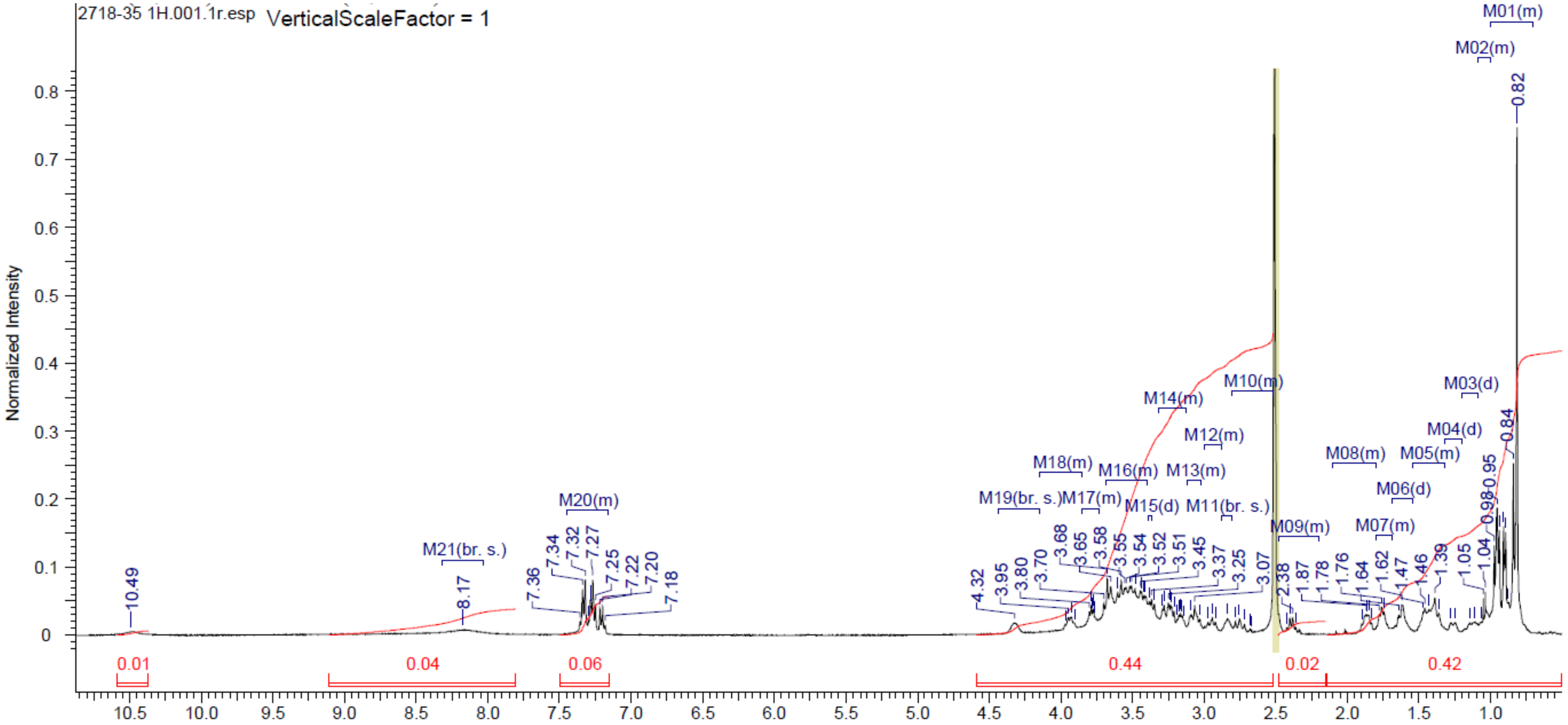

2718-35 1H.001.1r.esp
VerticalScaleFactor = 1
Normalized Intensity
M01(m)
M02(m)
M03(d)
M04(d)
M05(m)
M06(d)
M07(m)
M08(m)
M09(m)
M10(m)
M11(br. s.)
M12(m)
M13(m)
M14(m)
M15(d)
M16(m)
M17(m)
M18(m)
M19(br. s.)
M20(m)
M21(br. s.)
0.01
0.04
0.06
0.44
0.02
0.42

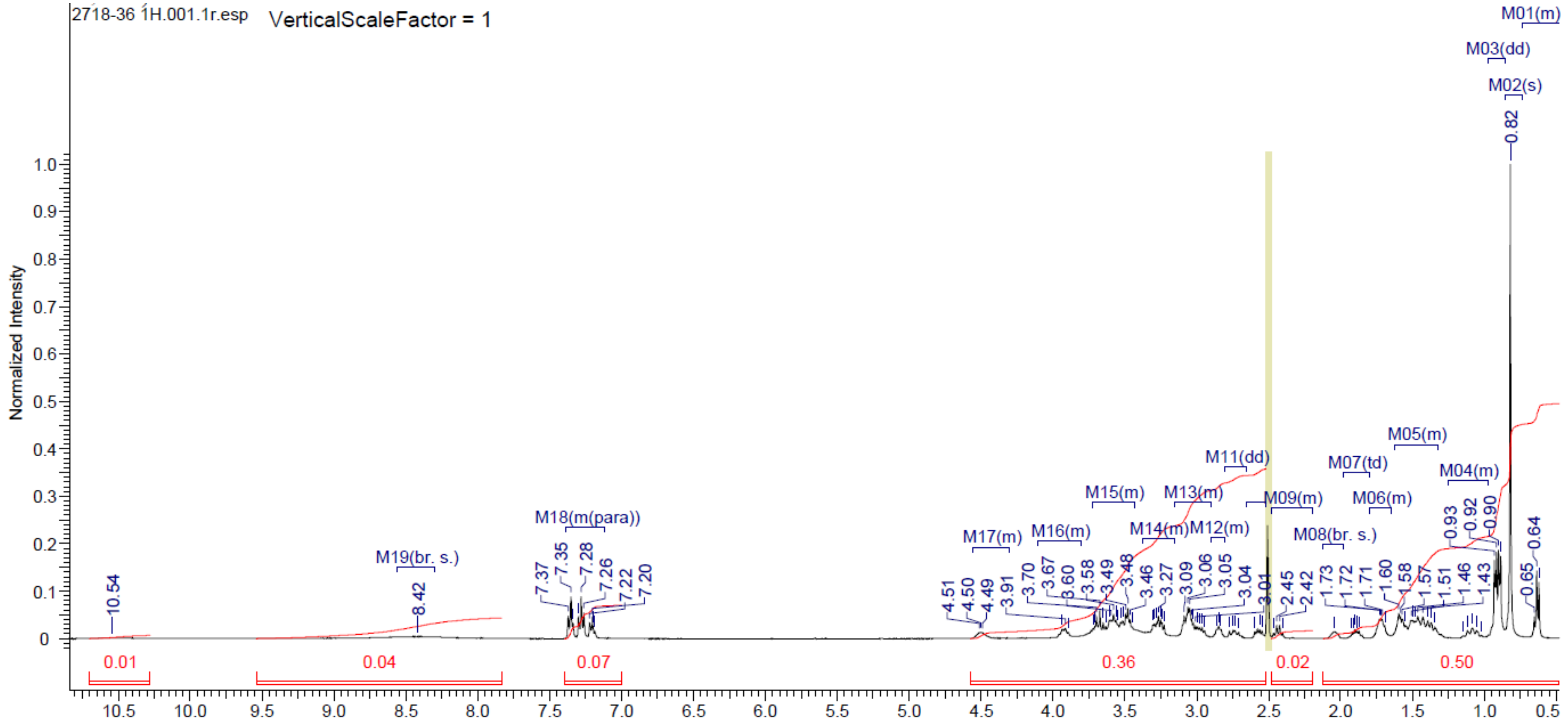

2718-36 1H.001.1r.esp
VerticalScaleFactor = 1
Normalized Intensity
M01(m)
M03(dd)
M02(s)
M05(m)
M11(dd)
M07(td)
M04(m)
M15(m)
M13(m)
M09(m)
M06(m)
M18(m(para))
M17(m)
M16(m)
M14(m)
M12(m)
M08(br. s.)
M19(br. s.)
0.01
0.04
0.07
0.36
0.02
0.50

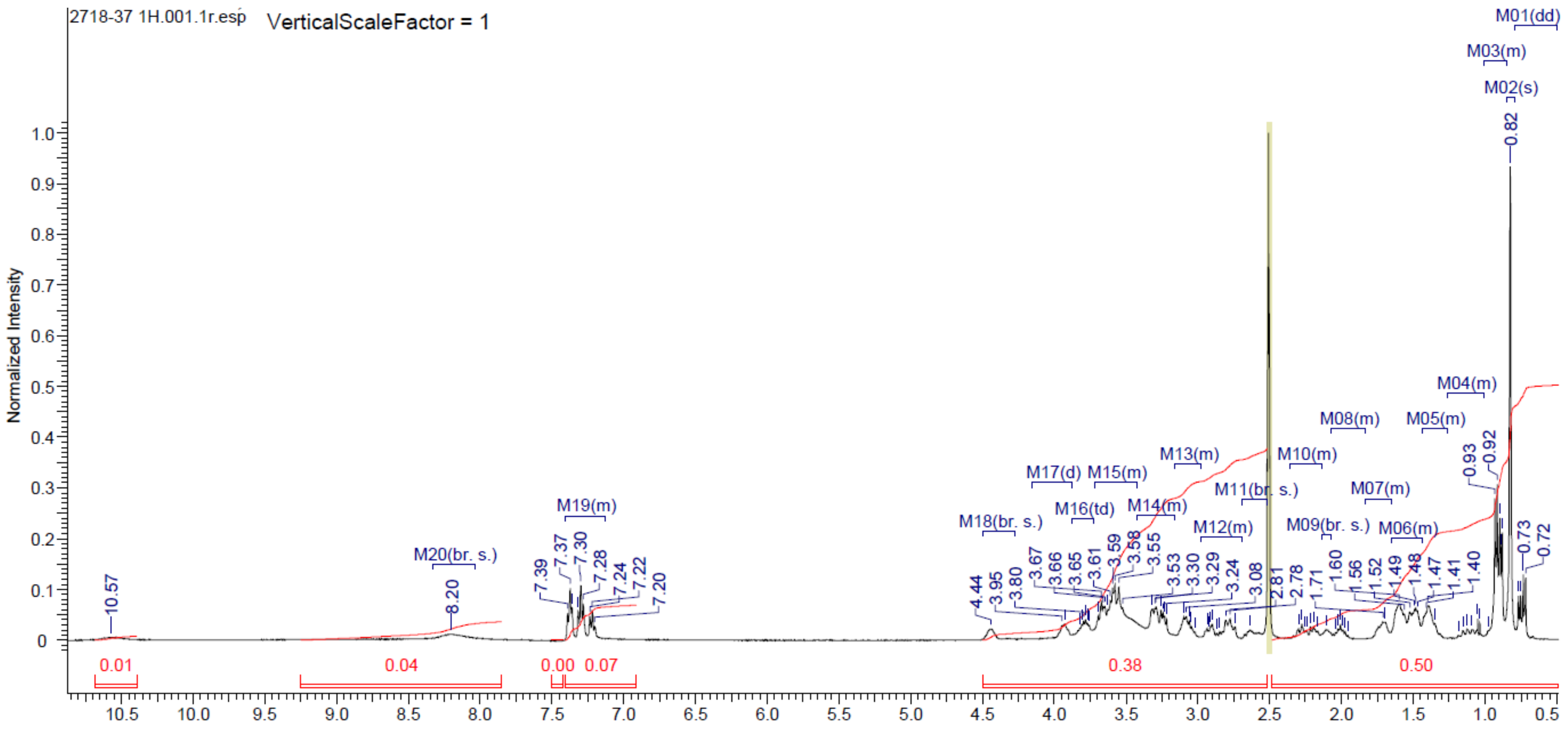

2718-37 1H.001.1r.esp
VerticalScaleFactor = 1
Normalized Intensity
M01(dd)
M02(s)
M03(m)
M04(m)
M05(m)
M06(m)
M07(m)
M08(m)
M09(br. s.)
M10(m)
M11(br. s.)
M12(m)
M13(m)
M14(m)
M15(m)
M16(td)
M17(d)
M18(br. s.)
M19(m)
M20(br. s.)
0.01
0.04
0.00
0.07
0.38
0.50

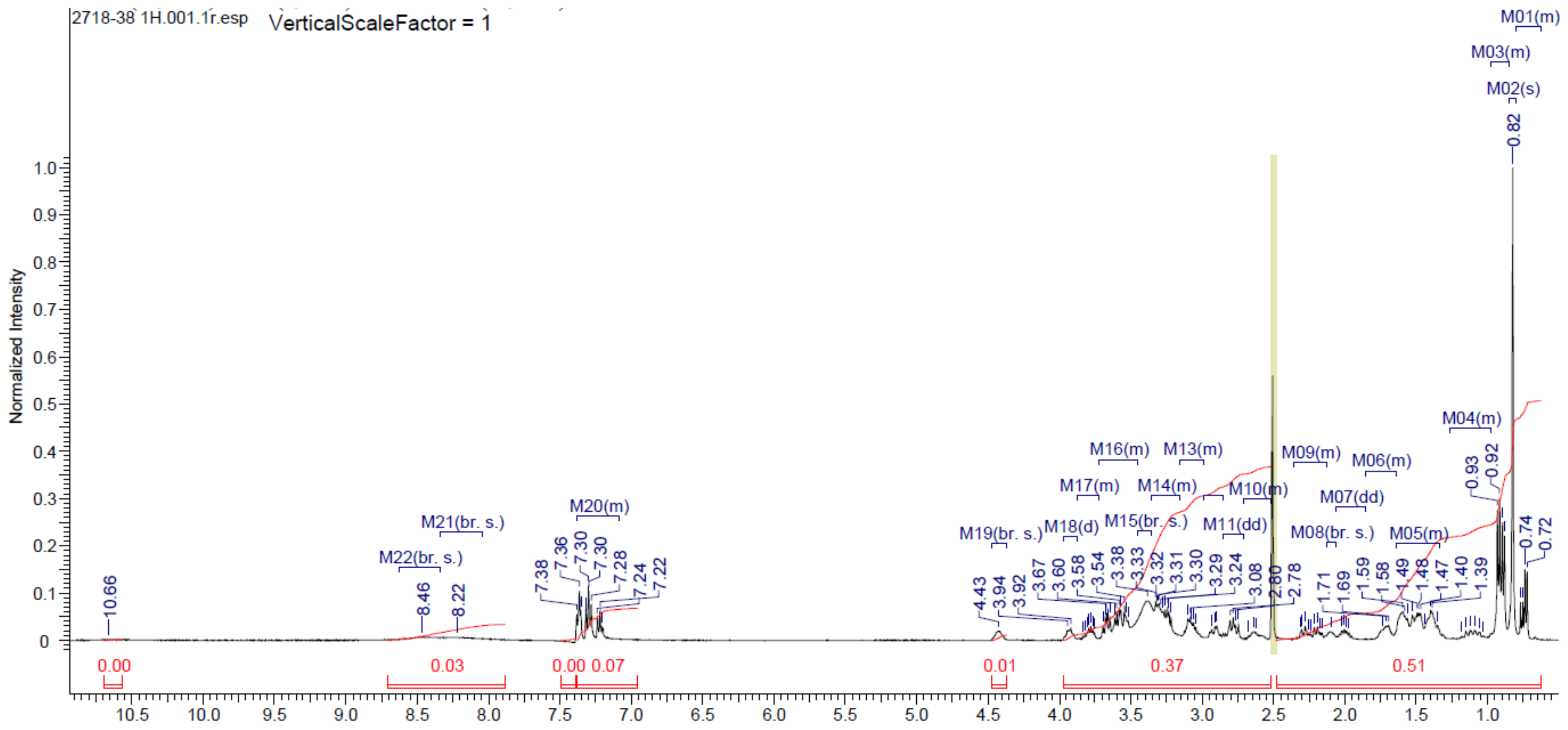

2718-38 1H.001.1r.esp
VerticalScaleFactor = 1
Normalized Intensity
M01(m)
M02(s)
M03(m)
M04(m)
M05(m)
M06(m)
M07(dd)
M08(br. s.)
M09(m)
M10(m)
M11(dd)
M13(m)
M14(m)
M15(br. s.)
M16(m)
M17(m)
M18(d)
M19(br. s.)
M20(m)
M21(br. s.)
M22(br. s.)
0.00
0.03
0.00 0.07
0.01
0.37
0.51

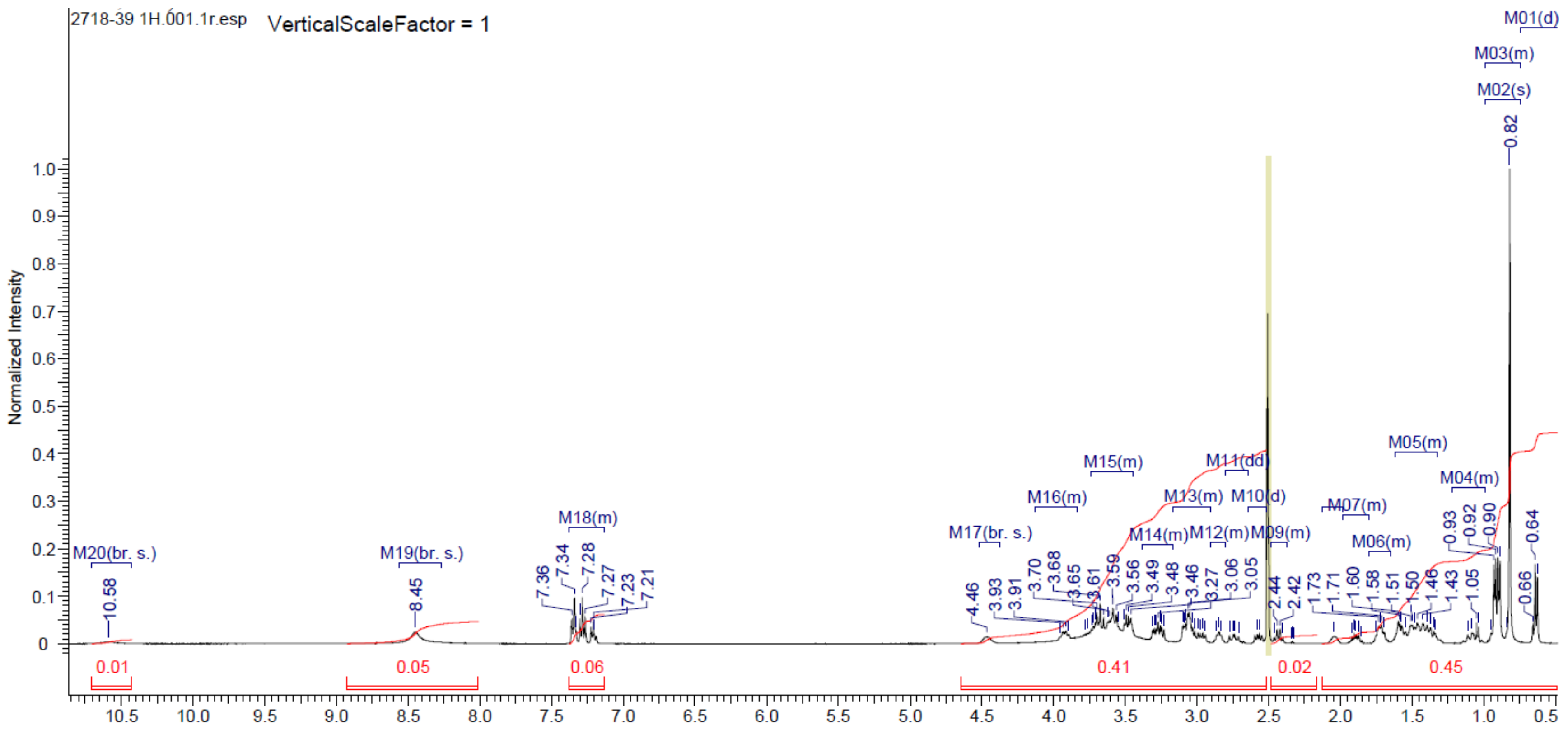

2718-39 1H.001.1r.esp
VerticalScaleFactor = 1
Normalized Intensity
M01(d)
M02(s)
M03(m)
M04(m)
M05(m)
M06(m)
M07(m)
M09(m)
M10(d)
M11(dd)
M12(m)
M13(m)
M14(m)
M15(m)
M16(m)
M17(br. s.)
M18(m)
M19(br. s.)
M20(br. s.)
0.01
0.05
0.06
0.41
0.02
0.45

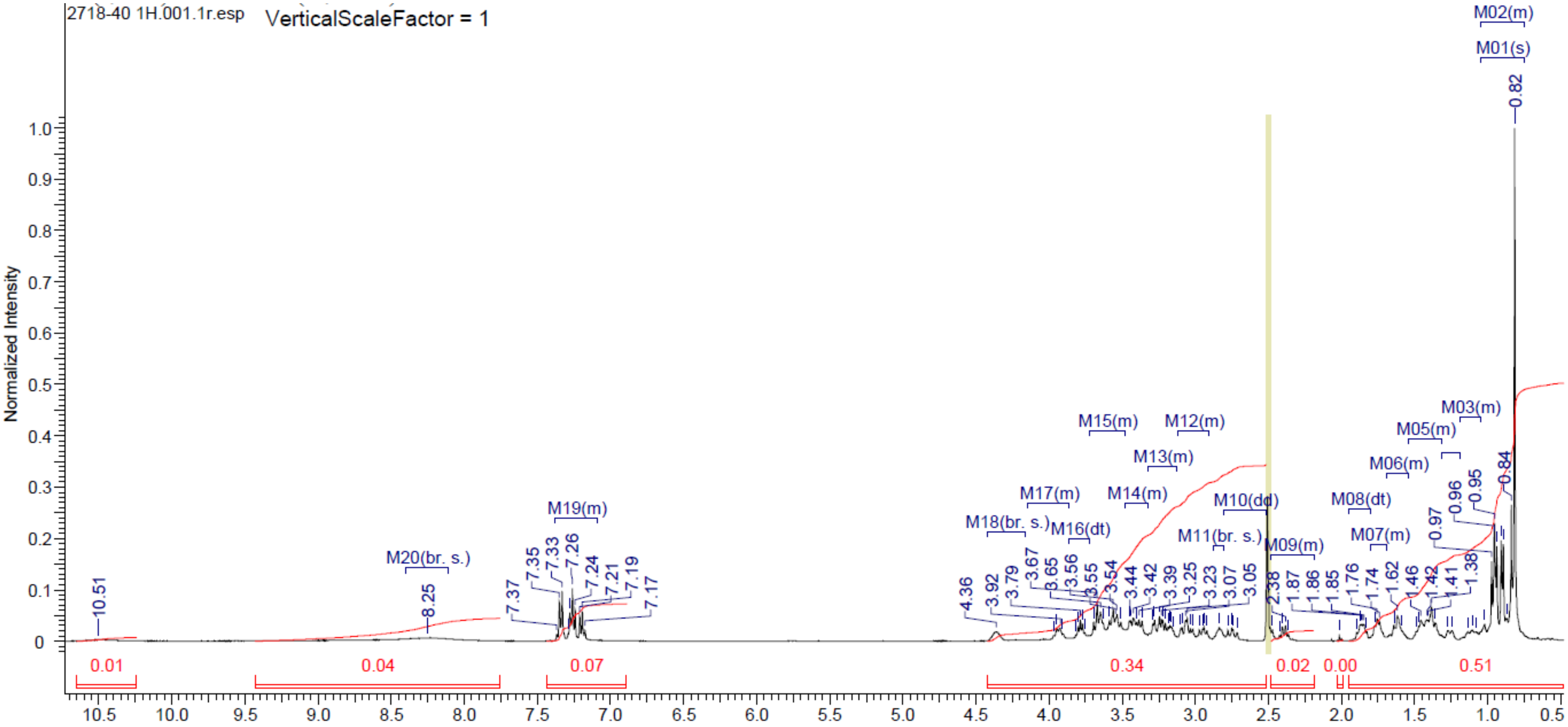

2718-40 1H.001.1r.esp
VerticalScaleFactor = 1
Normalized Intensity
M01(s)
M02(m)
M03(m)
M05(m)
M06(m)
M07(m)
M08(dt)
M09(m)
M10(dd)
M11(br. s.)
M12(m)
M13(m)
M14(m)
M15(m)
M16(dt)
M17(m)
M18(br. s.)
M19(m)
M20(br. s.)
0.01
0.04
0.07
0.34
0.02
0.00
0.51

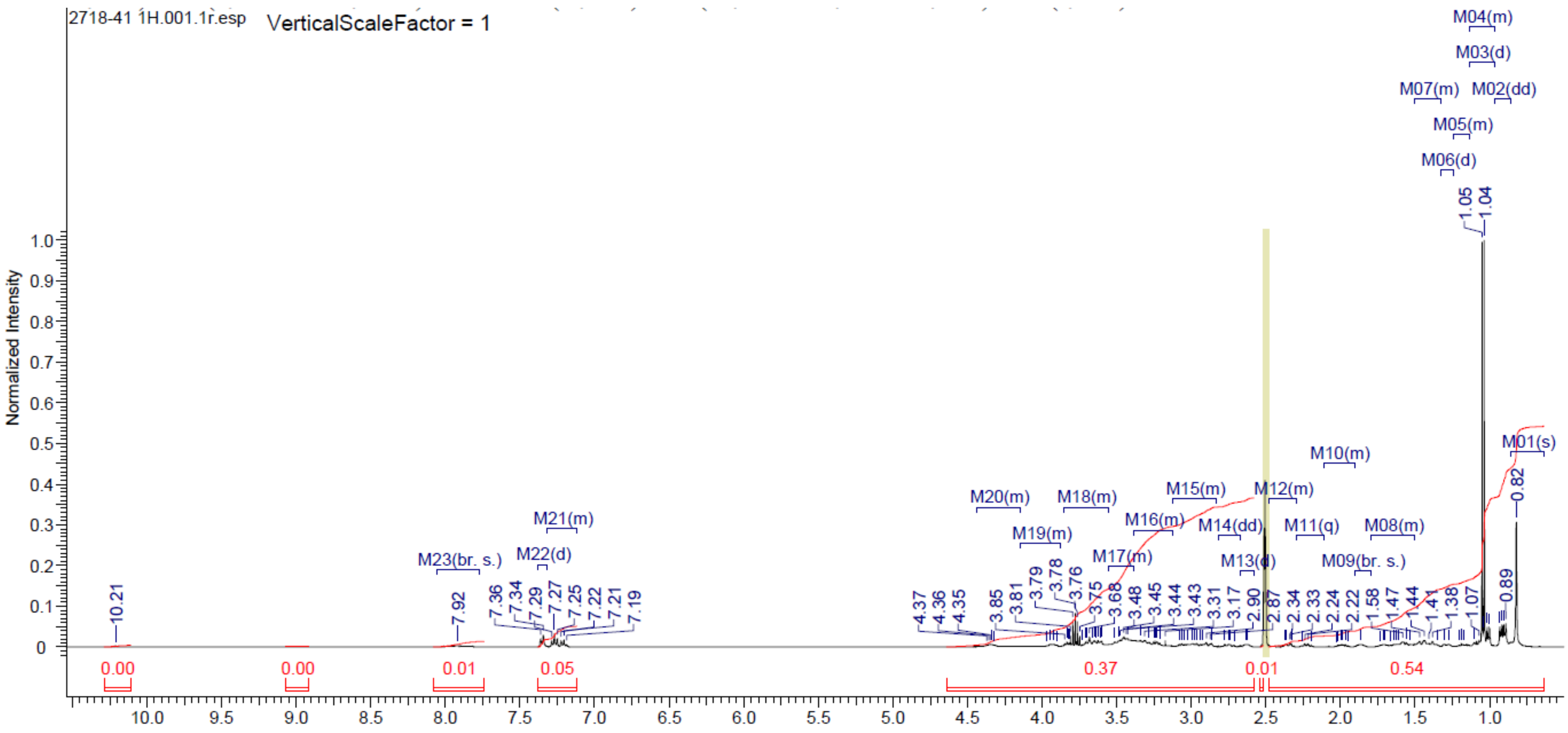
2718-41 1H.001.1r.esp
VerticalScaleFactor = 1
Normalized Intensity
M01(s)
M02(dd)
M03(d)
M04(m)
M05(m)
M06(d)
M07(m)
M08(m)
M09(br. s.)
M10(m)
M11(q)
M12(m)
M13(d)
M14(dd)
M15(m)
M16(m)
M17(m)
M18(m)
M19(m)
M20(m)
M21(m)
M22(d)
M23(br. s.)
0.00
0.00
0.01
0.05
0.37
0.01
0.54

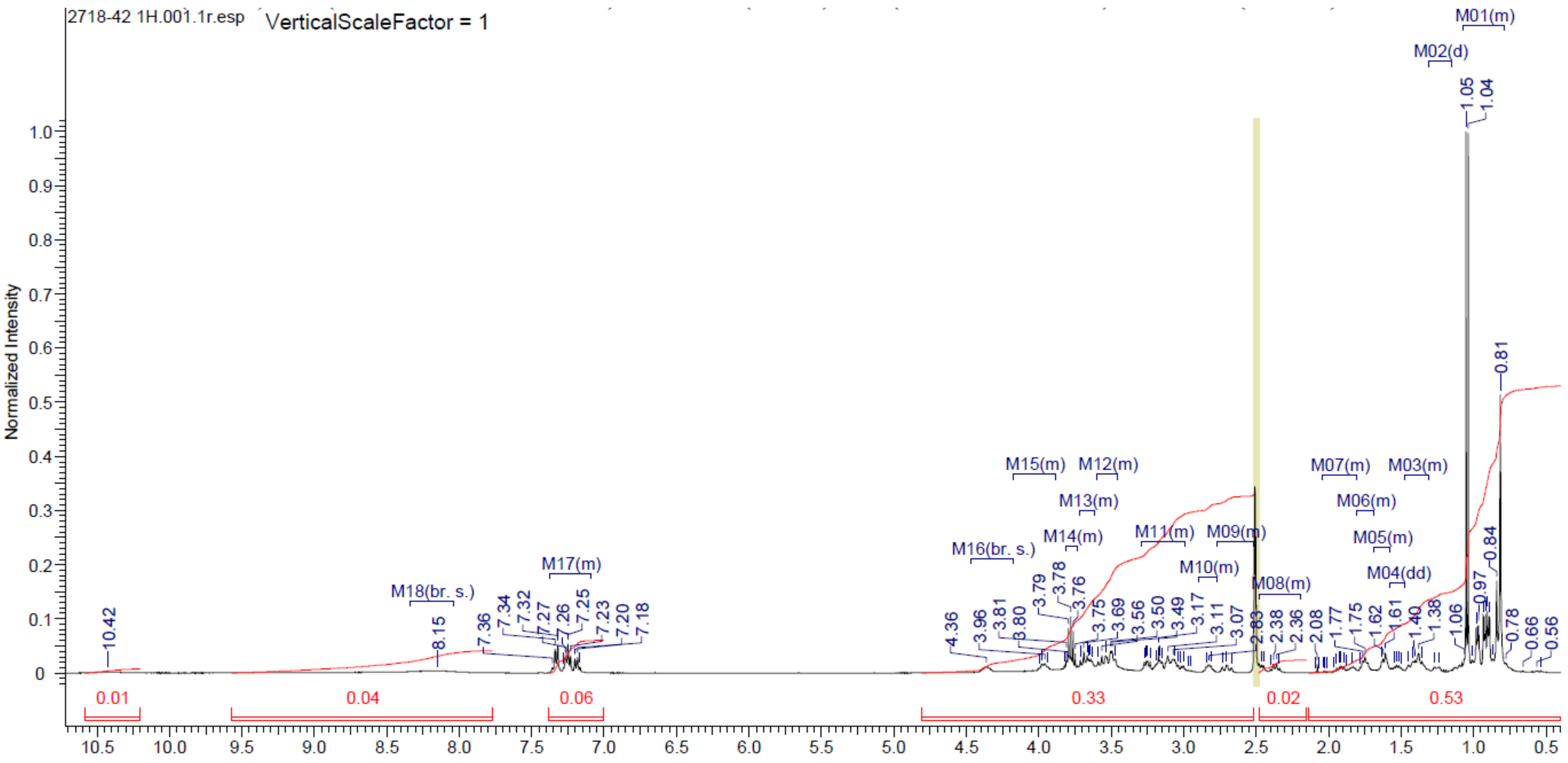
2718-42 1H.001.1r.esp
VerticalScaleFactor = 1
Normalized Intensity
M01(m)
M02(d)
M03(m)
M04(dd)
M05(m)
M06(m)
M07(m)
M08(m)
M09(m)
M10(m)
M11(m)
M12(m)
M13(m)
M14(m)
M15(m)
M16(br. s.)
M17(m)
M18(br. s.)
0.01
0.04
0.06
0.33
0.02
0.53

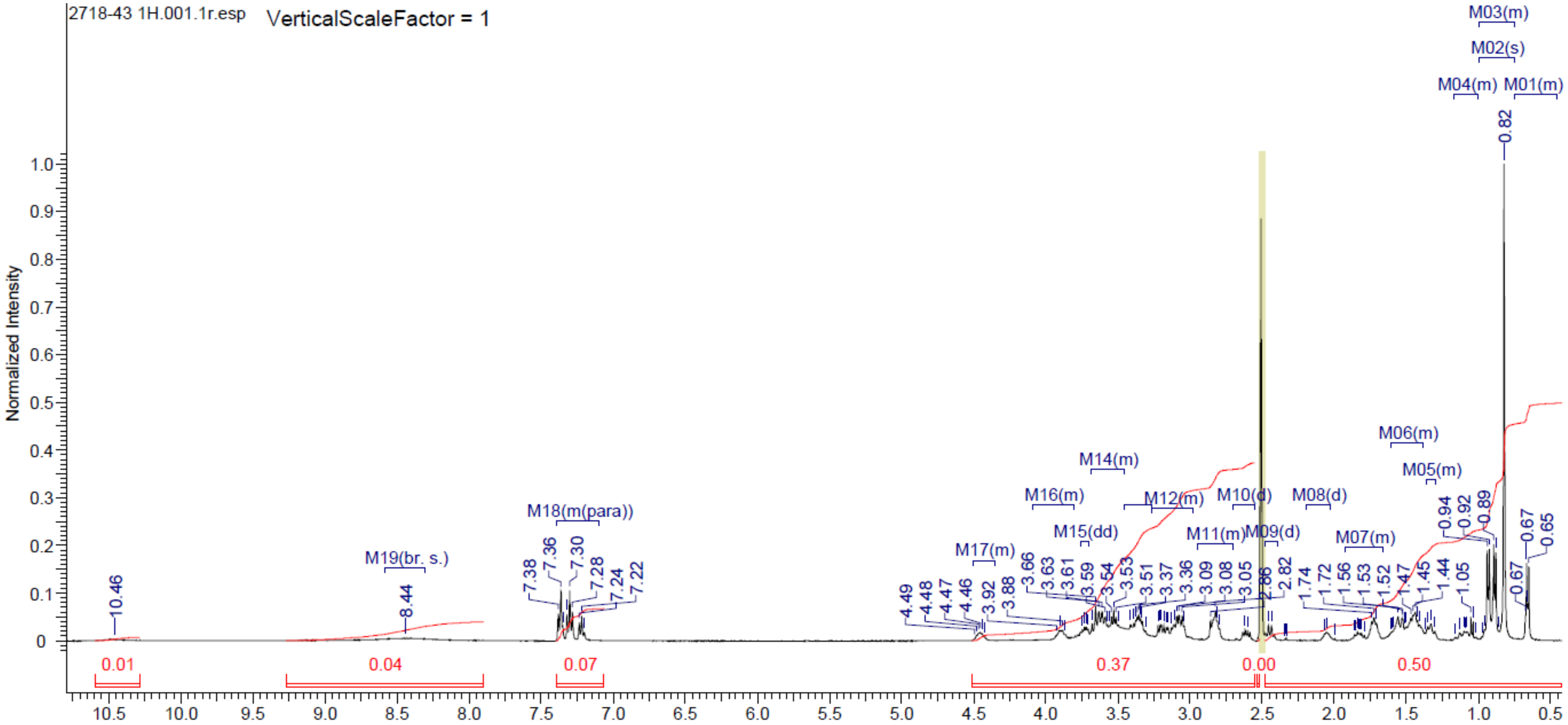
2718-43 1H.001.1r.esp
VerticalScaleFactor = 1
Normalized Intensity
M01(m)
M02(s)
M03(m)
M04(m)
M05(m)
M06(m)
M07(m)
M08(d)
M09(d)
M10(d)
M11(m)
M12(m)
M14(m)
M15(dd)
M16(m)
M17(m)
M18(m(para))
M19(br. s.)
0.01
0.04
0.07
0.37
0.00
0.50

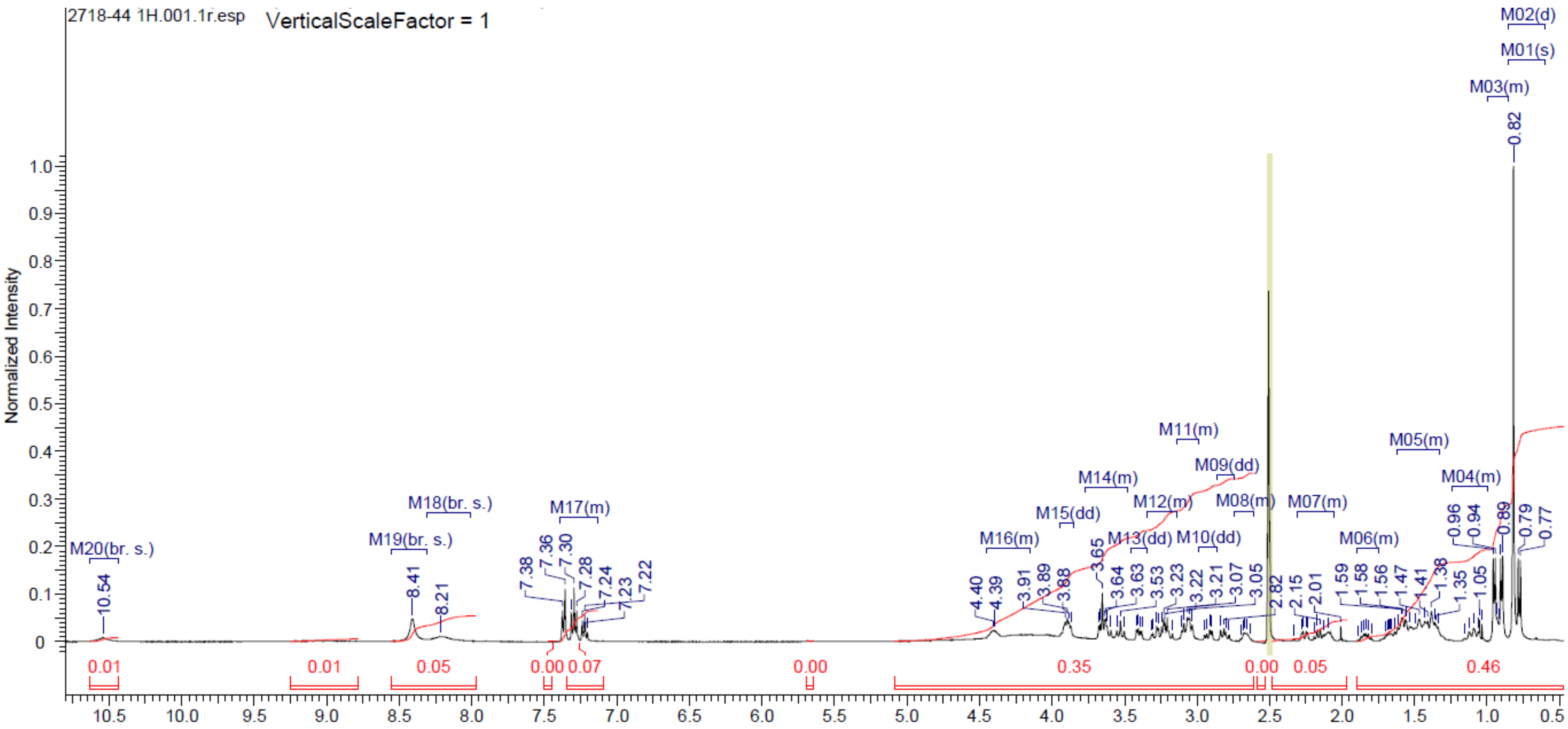

2718-44 1H.001.1r.esp
VerticalScaleFactor = 1
Normalized Intensity
M01(s)
M02(d)
M03(m)
M04(m)
M05(m)
M06(m)
M07(m)
M08(m)
M09(dd)
M10(dd)
M11(m)
M12(m)
M13(dd)
M14(m)
M15(dd)
M16(m)
M17(m)
M18(br. s.)
M19(br. s.)
M20(br. s.)
0.01
0.01
0.05
0.00
0.07
0.00
0.35
0.00
0.05
0.46

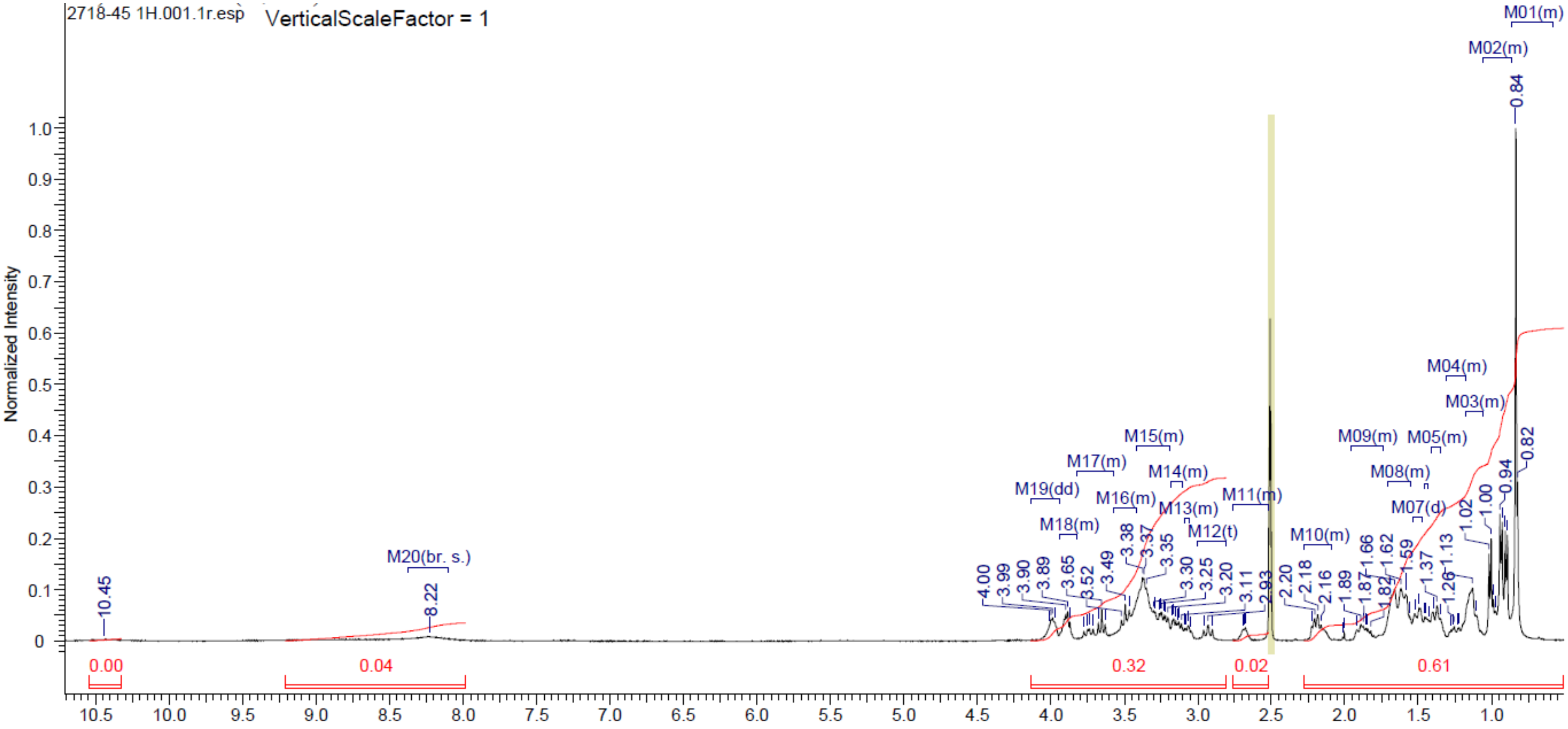
2718-45 1H.001.1r.esp
VerticalScaleFactor = 1
Normalized Intensity
M01(m)
M02(m)
M03(m)
M04(m)
M05(m)
M07(d)
M08(m)
M09(m)
M10(m)
M11(m)
M12(t)
M13(m)
M14(m)
M15(m)
M16(m)
M17(m)
M18(m)
M19(dd)
M20(br. s.)
0.00
0.04
0.32
0.02
0.61

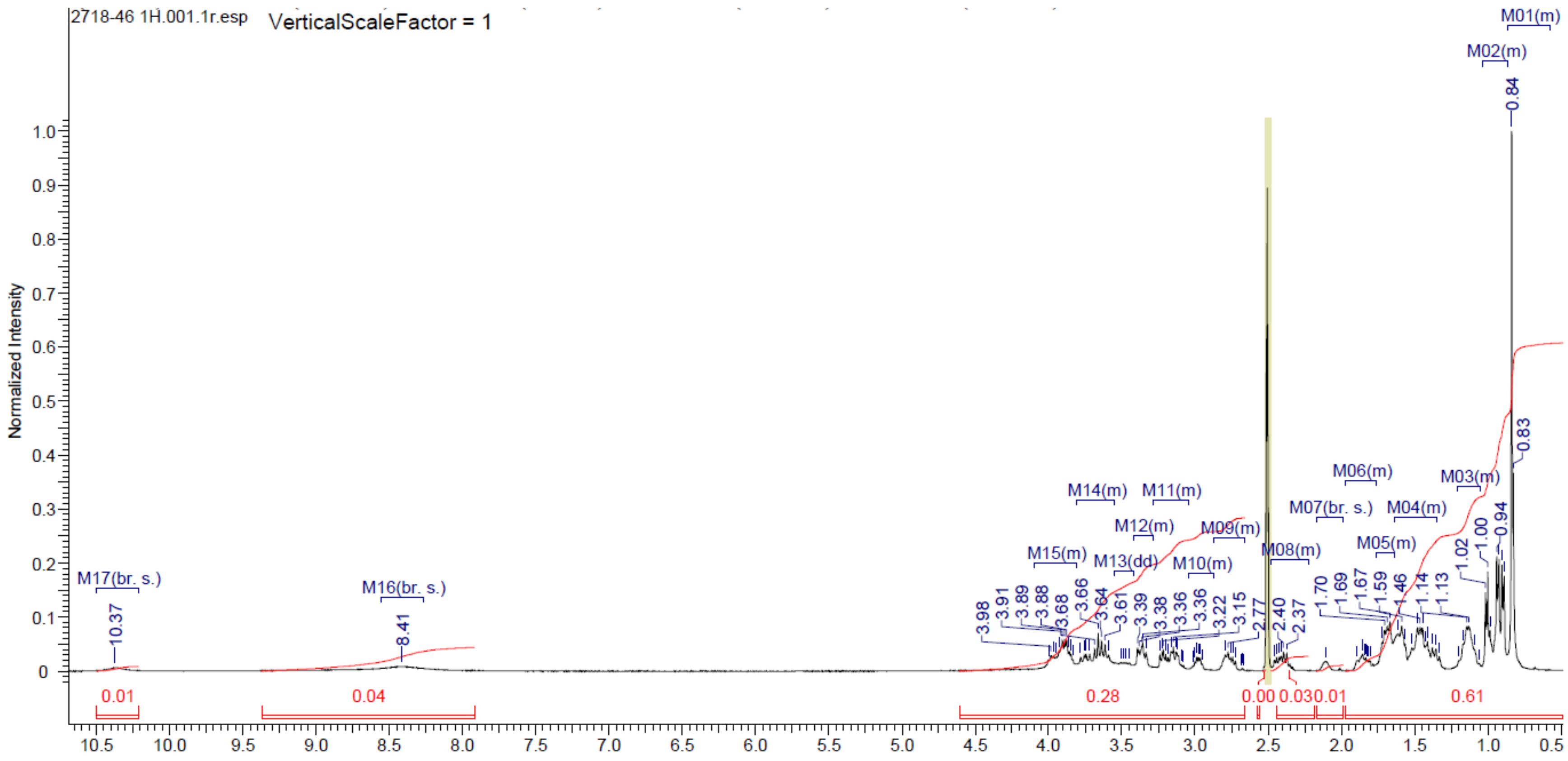
2718-46 1H.001.1r.esp
VerticalScaleFactor = 1
Normalized Intensity
M01(m)
M02(m)
M03(m)
M04(m)
M05(m)
M06(m)
M07(br. s.)
M08(m)
M09(m)
M10(m)
M11(m)
M12(m)
M13(dd)
M14(m)
M15(m)
M16(br. s.)
M17(br. s.)
0.01
0.04
0.28
0.00
0.03
0.01
0.61

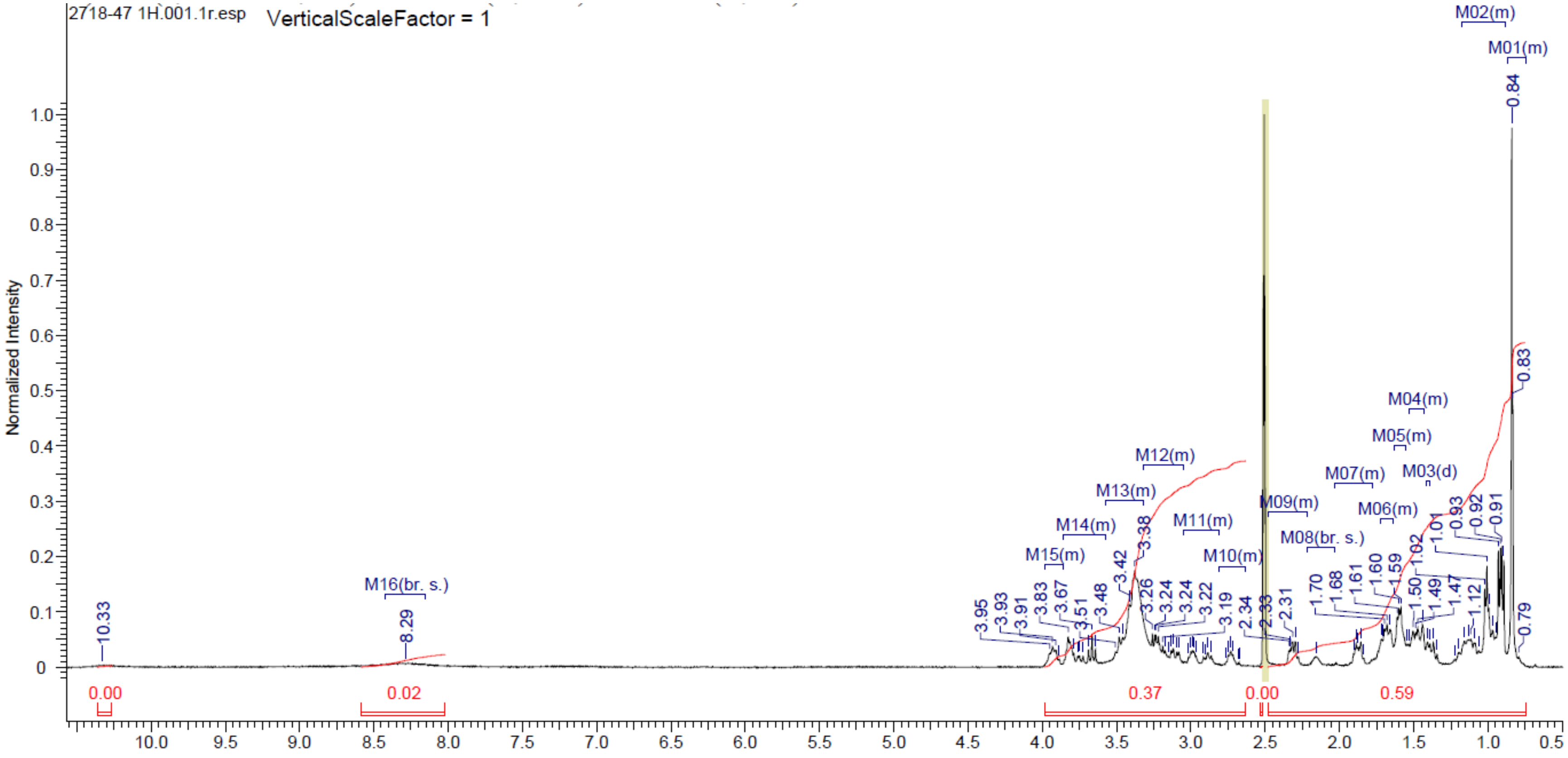
2718-47 1H.001.1r.esp
VerticalScaleFactor = 1
Normalized Intensity
M01(m)
M02(m)
M03(d)
M04(m)
M05(m)
M06(m)
M07(m)
M08(br. s.)
M09(m)
M10(m)
M11(m)
M12(m)
M13(m)
M14(m)
M15(m)
M16(br. s.)
0.00
0.02
0.37
0.00
0.59

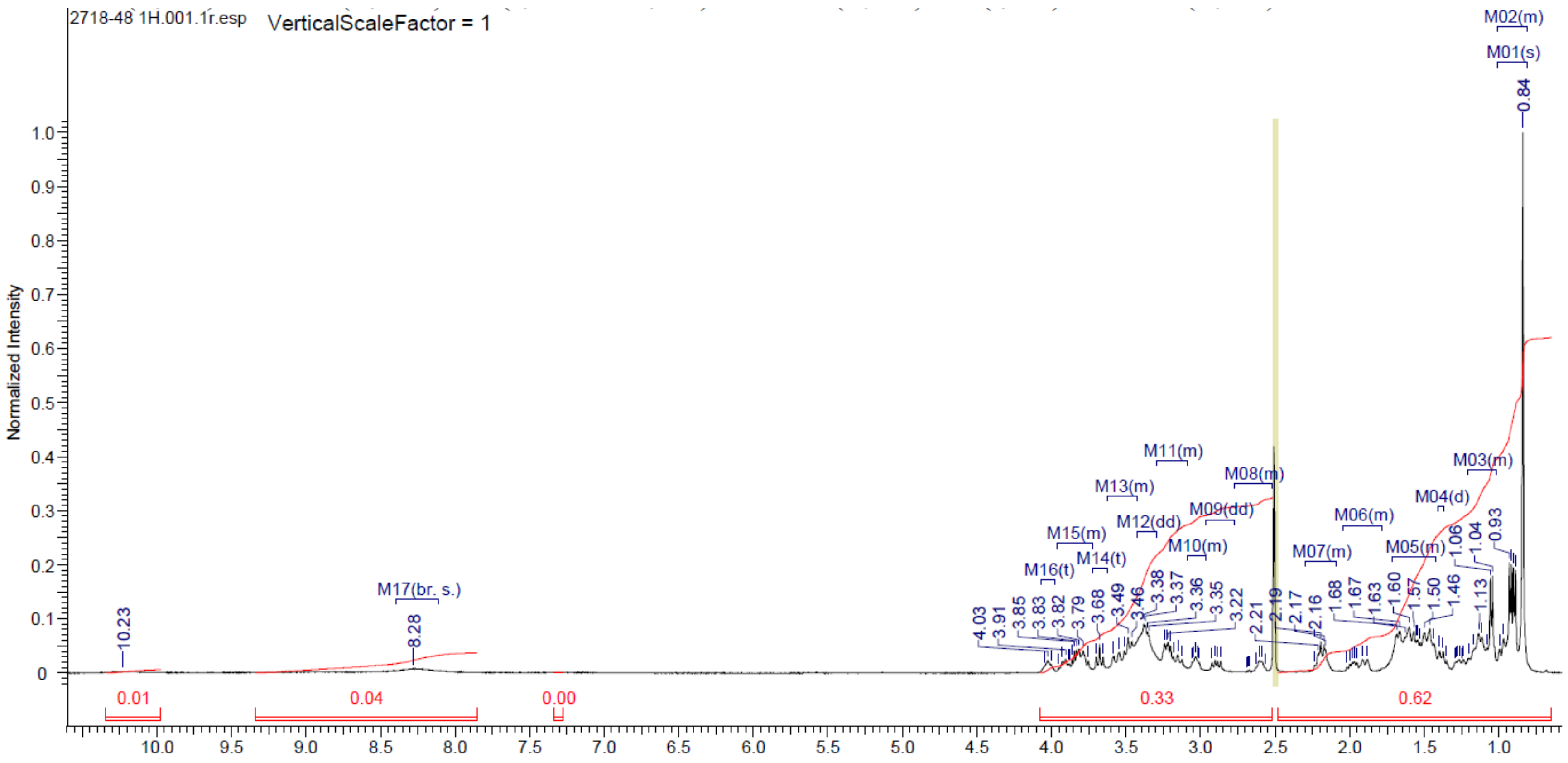

2718-48 1H.001.1r.esp
VerticalScaleFactor = 1
Normalized Intensity
M01(s)
M02(m)
M03(m)
M04(d)
M05(m)
M06(m)
M07(m)
M08(m)
M09(dd)
M10(m)
M11(m)
M12(dd)
M13(m)
M14(t)
M15(m)
M16(t)
M17(br. s.)
0.84
10.23
8.28
0.01
0.04
0.00
0.33
0.62

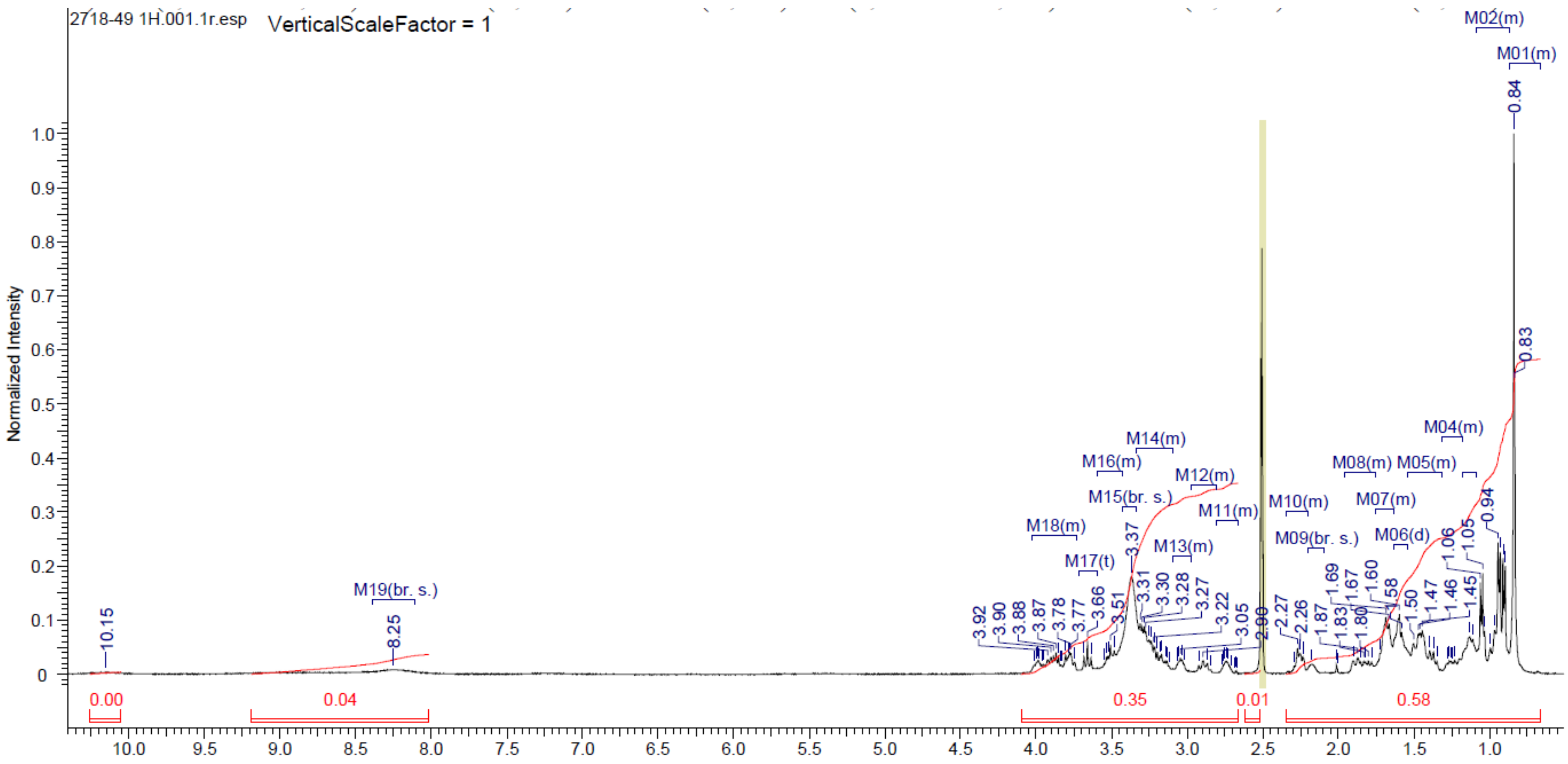

2718-49 1H.001.1r.esp
VerticalScaleFactor = 1
Normalized Intensity
M01(m)
M02(m)
M04(m)
M05(m)
M06(d)
M07(m)
M08(m)
M09(br. s.)
M10(m)
M11(m)
M12(m)
M13(m)
M14(m)
M15(br. s.)
M16(m)
M17(t)
M18(m)
M19(br. s.)
10.15
8.25
0.00
0.04
0.35
0.01
0.58

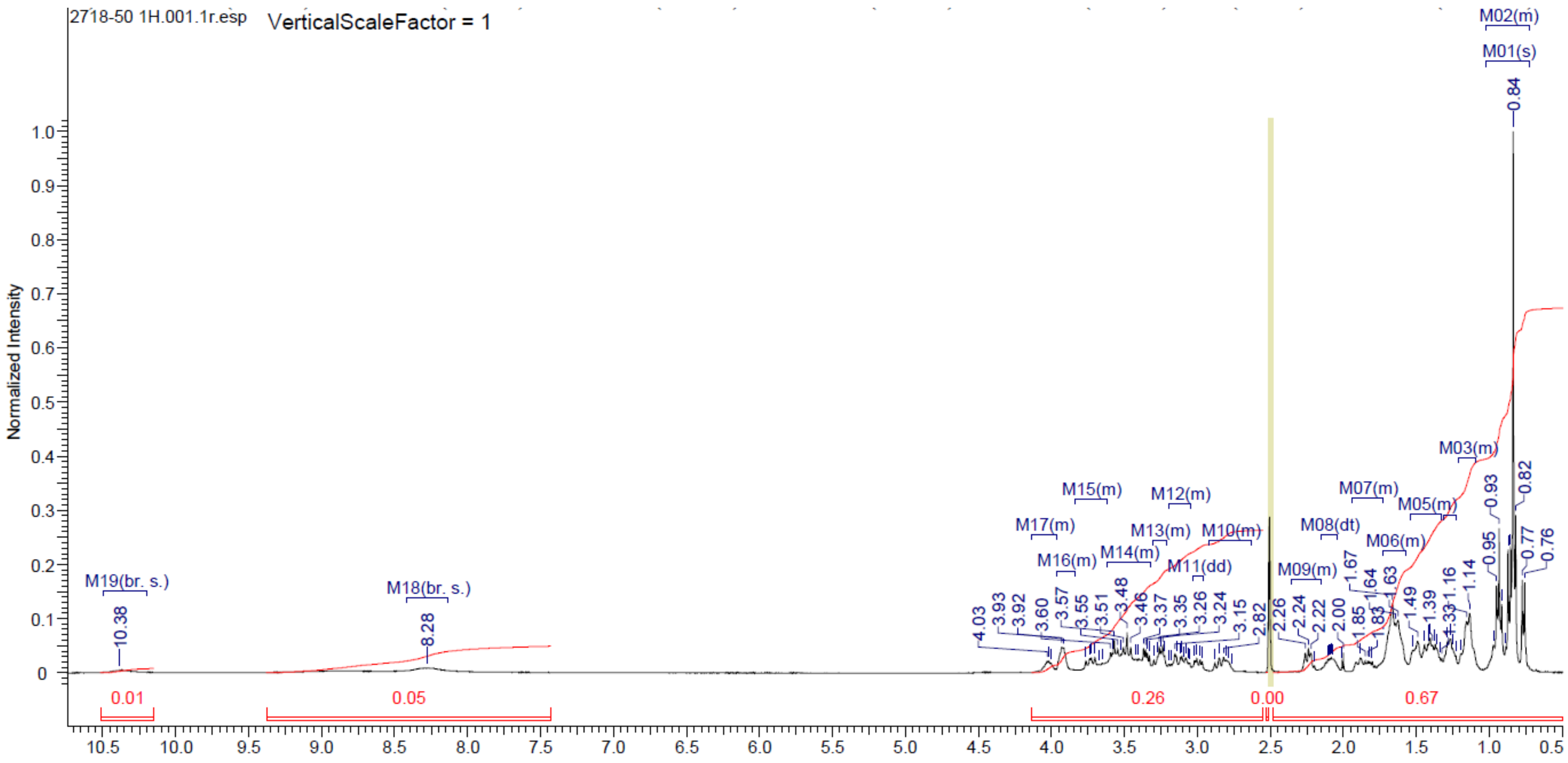

2718-50 1H.001.1r.esp
VerticalScaleFactor = 1
Normalized Intensity
M01(s)
M02(m)
M03(m)
M05(m)
M06(m)
M07(m)
M08(dt)
M09(m)
M10(m)
M11(dd)
M12(m)
M13(m)
M14(m)
M15(m)
M16(m)
M17(m)
M18(br. s.)
M19(br. s.)
0.01
0.05
0.26
0.00
0.67

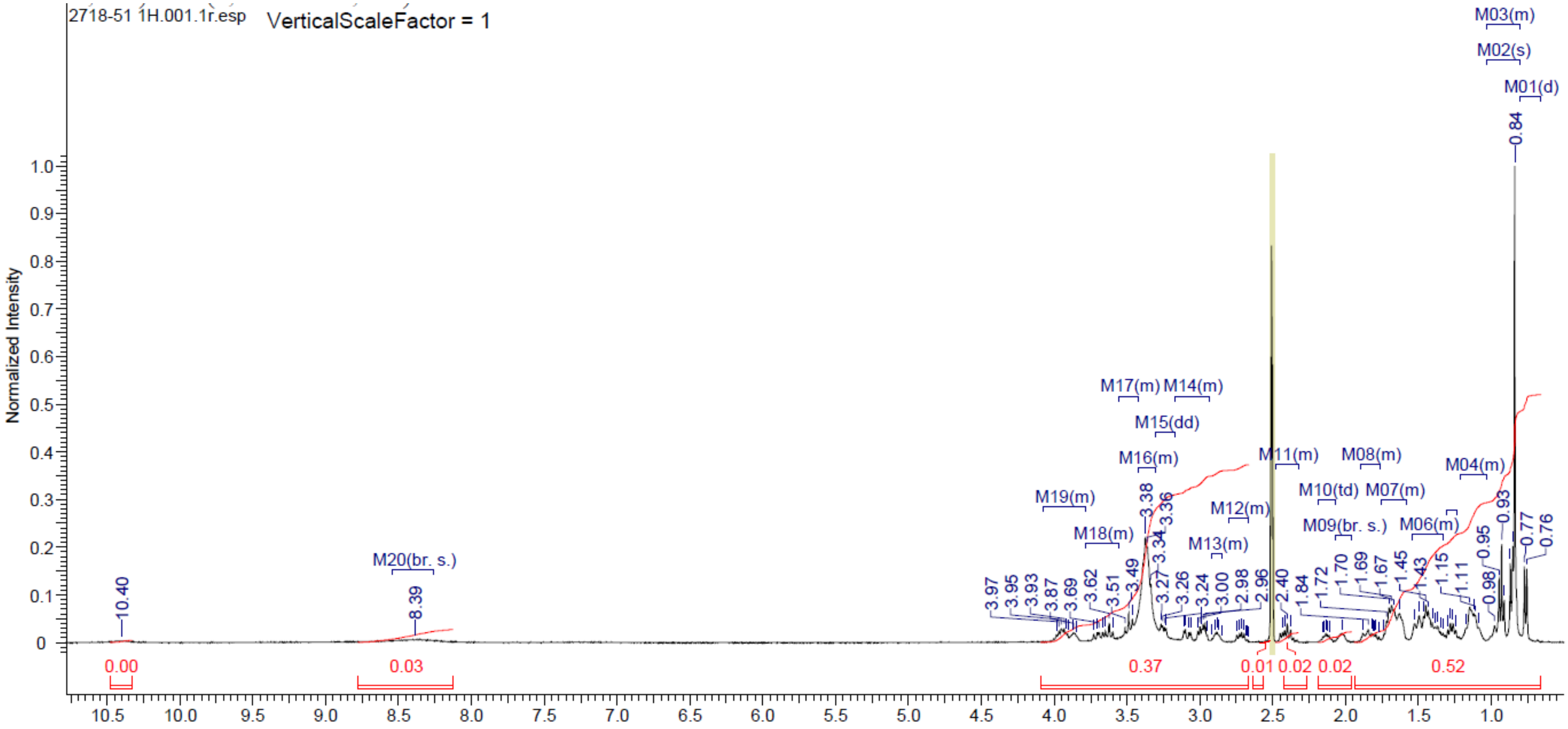

2718-51 1H.001.1r.esp
VerticalScaleFactor = 1
Normalized Intensity
M01(d)
M02(s)
M03(m)
M04(m)
M06(m)
M07(m)
M08(m)
M09(br. s.)
M10(td)
M11(m)
M12(m)
M13(m)
M14(m)
M15(dd)
M16(m)
M17(m)
M18(m)
M19(m)
M20(br. s.)
0.00
0.03
0.37
0.01
0.02
0.02
0.52

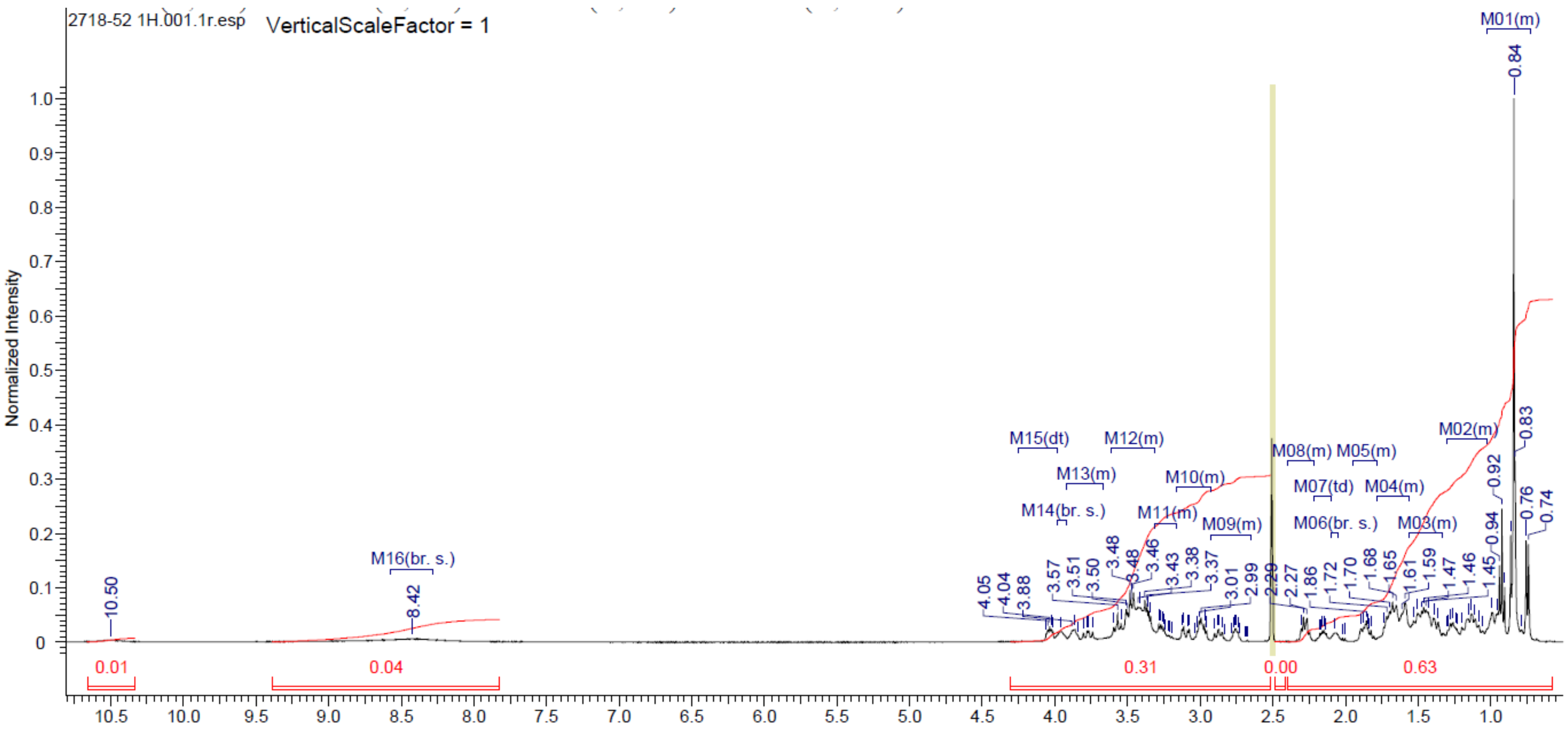

2718-52 1H.001.1r.esp
VerticalScaleFactor = 1
Normalized Intensity
M01(m)
M02(m)
M03(m)
M04(m)
M05(m)
M06(br. s.)
M07(td)
M08(m)
M09(m)
M10(m)
M11(m)
M12(m)
M13(m)
M14(br. s.)
M15(dt)
M16(br. s.)
0.01
0.04
0.31
0.00
0.63

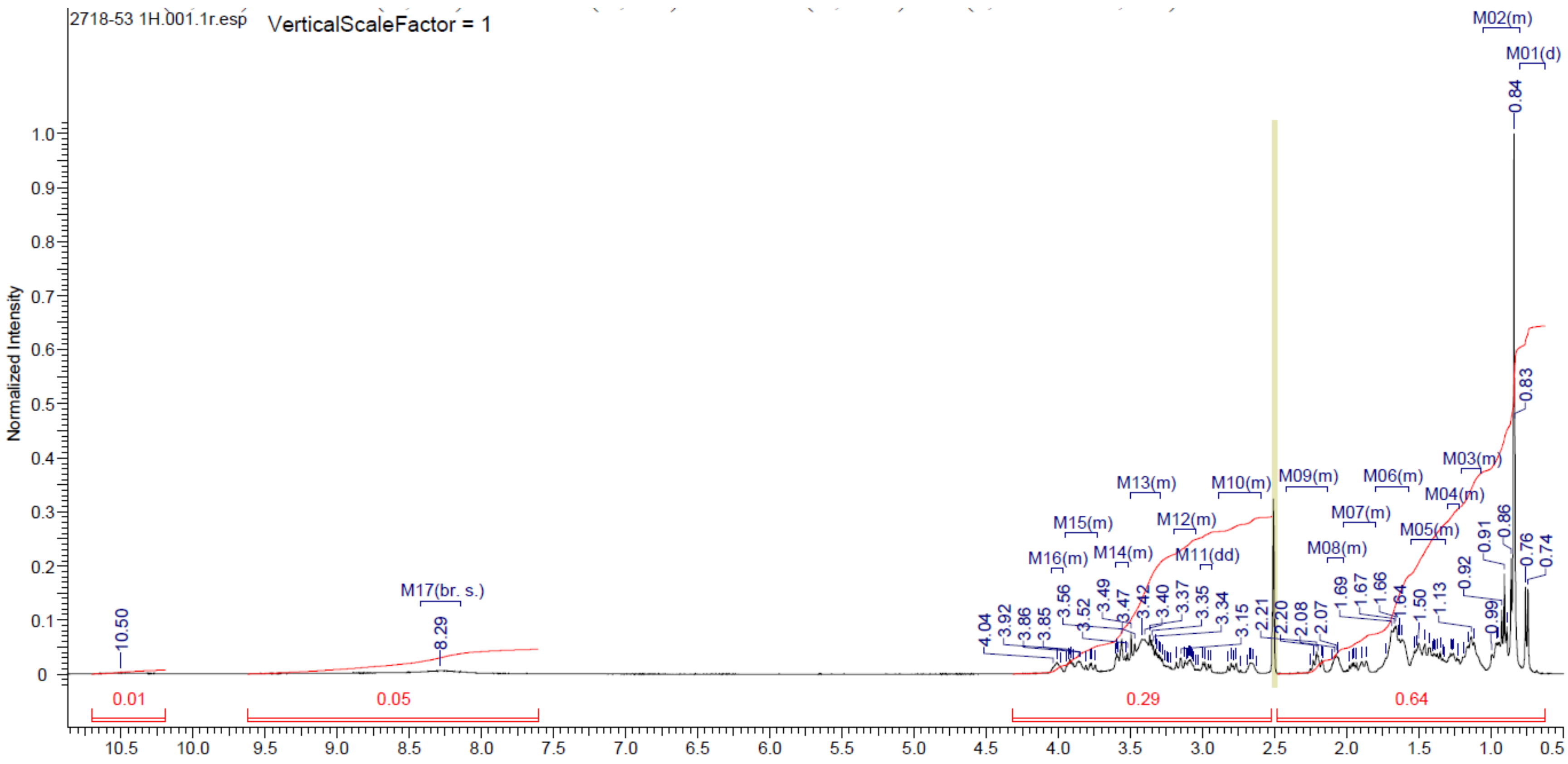
2718-53 1H.001.1r.esp
VerticalScaleFactor = 1
Normalized Intensity
M01(d)
M02(m)
M03(m)
M04(m)
M05(m)
M06(m)
M07(m)
M08(m)
M09(m)
M10(m)
M11(dd)
M12(m)
M13(m)
M14(m)
M15(m)
M16(m)
M17(br. s.)
0.01
0.05
0.29
0.64

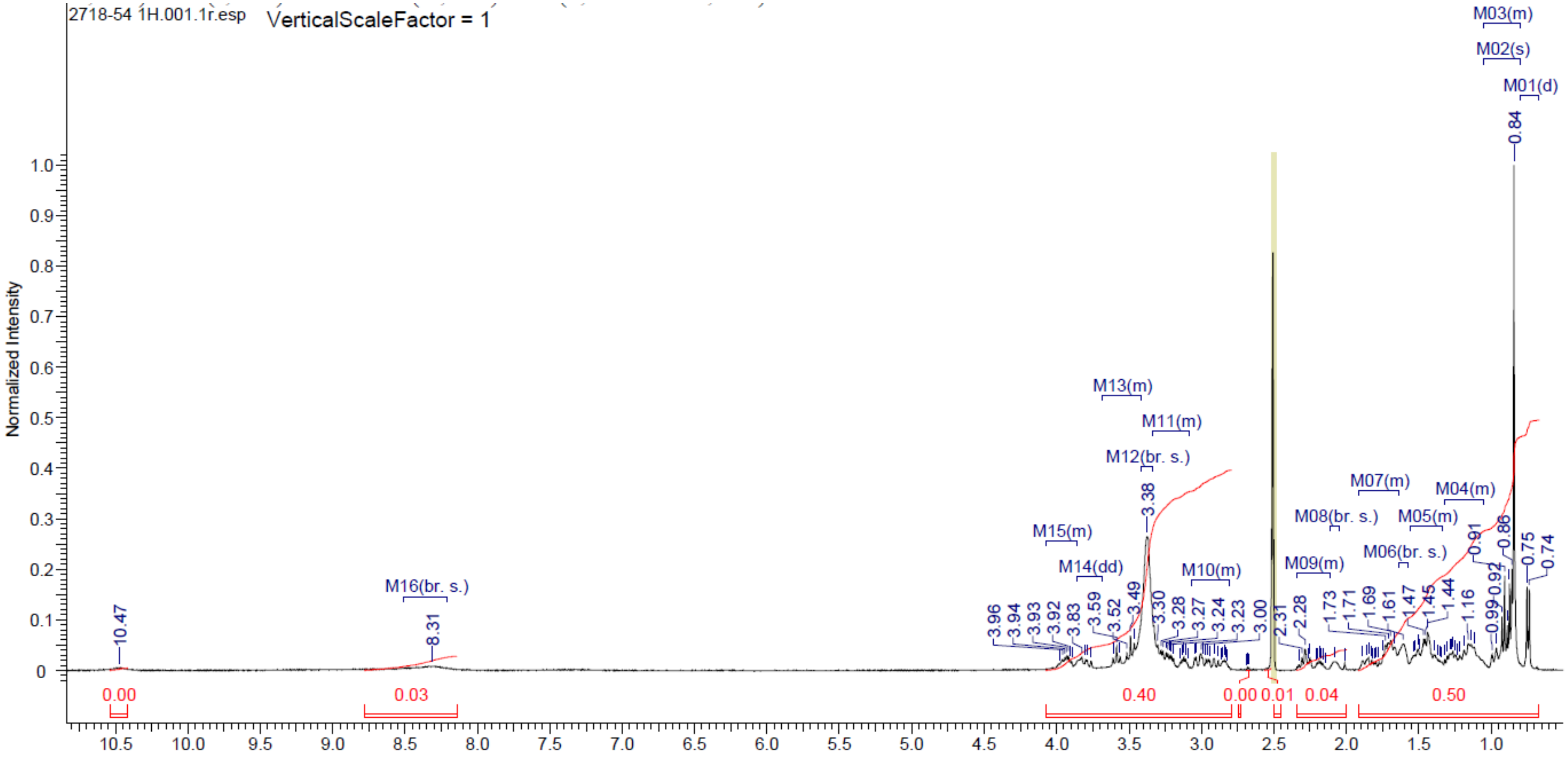

2718-54 1H.001.1r.esp
VerticalScaleFactor = 1
Normalized Intensity
M01(d)
M02(s)
M03(m)
M04(m)
M05(m)
M06(br. s.)
M07(m)
M08(br. s.)
M09(m)
M10(m)
M11(m)
M12(br. s.)
M13(m)
M14(dd)
M15(m)
M16(br. s.)
10.47
8.31
3.96
3.94
3.93
3.92
3.83
3.59
3.52
3.49
3.38
3.30
3.28
3.27
3.24
3.23
3.00
2.31
2.28
1.73
1.71
1.69
1.61
1.47
1.45
1.44
1.16
0.99
0.92
0.91
0.86
0.84
0.75
0.74
0.00
0.03
0.40
0.00
0.01
0.04
0.50

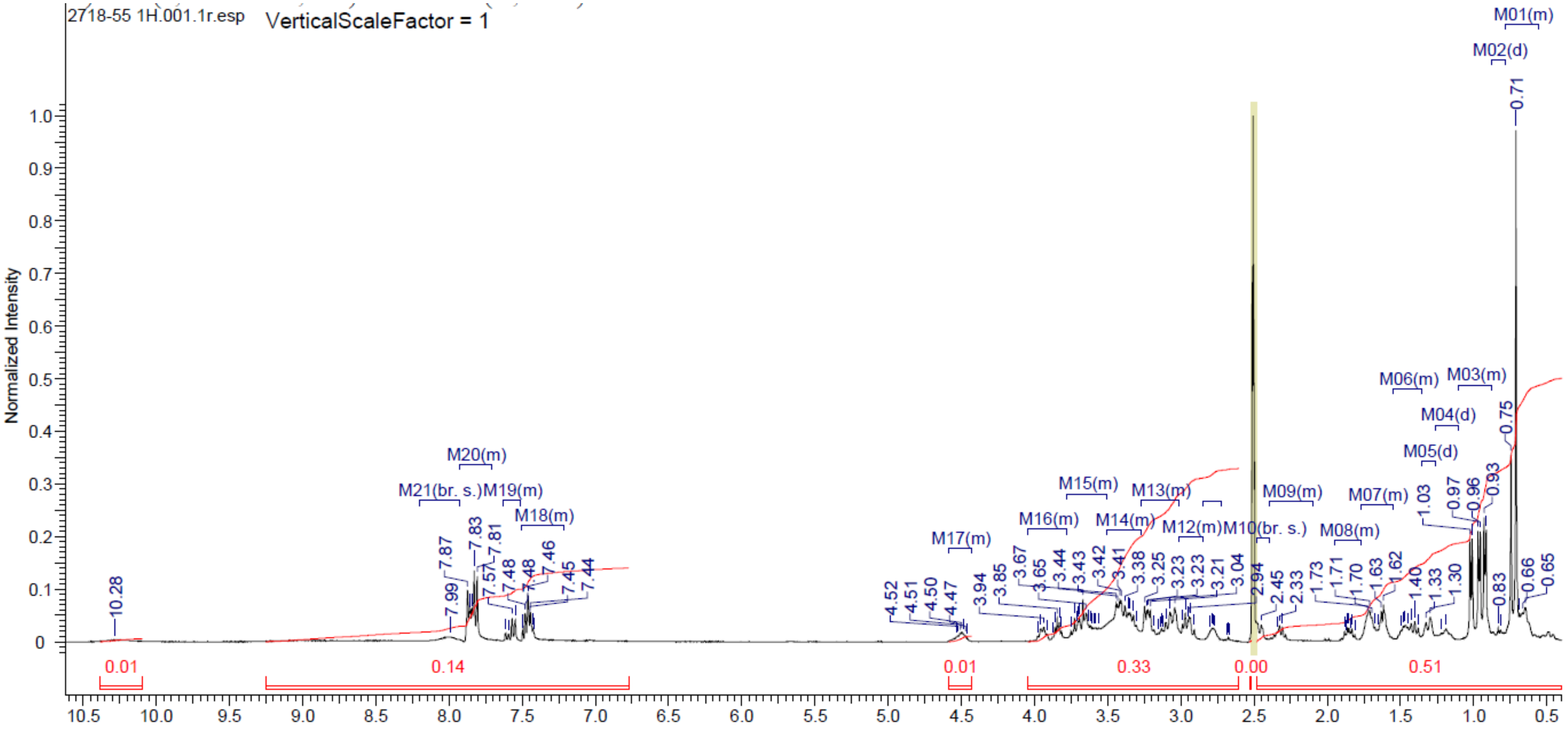

2718-55 1H.001.1r.esp
VerticalScaleFactor = 1
Normalized Intensity
M01(m)
M02(d)
M03(m)
M04(d)
M05(d)
M06(m)
M07(m)
M08(m)
M09(m)
M10(br. s.)
M12(m)
M13(m)
M14(m)
M15(m)
M16(m)
M17(m)
M18(m)
M19(m)
M20(m)
M21(br. s.)
0.01
0.14
0.01
0.33
0.00
0.51

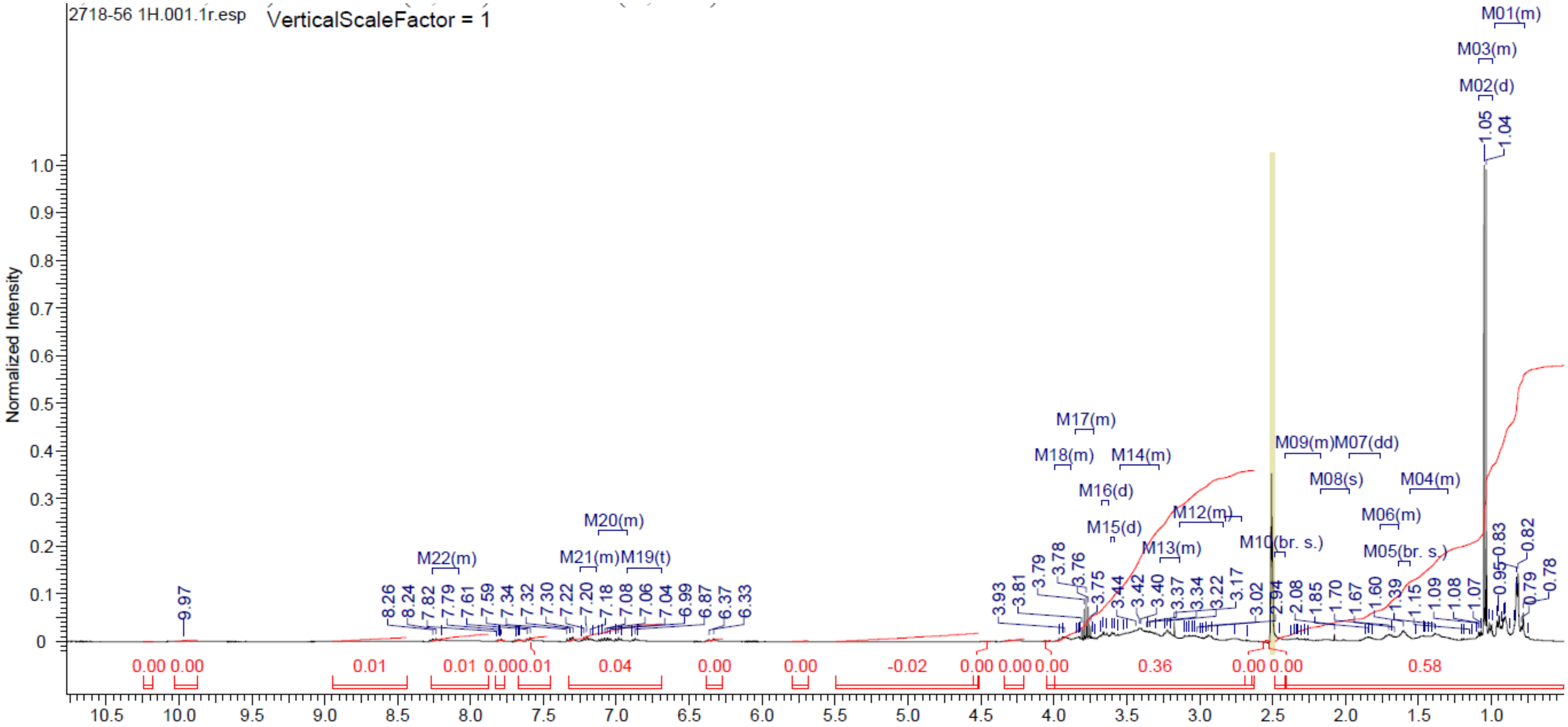

2718-56 1H.001.1r.esp
VerticalScaleFactor = 1
Normalized Intensity
M01(m)
M02(d)
M03(m)
M04(m)
M05(br. s.)
M06(m)
M07(dd)
M08(s)
M09(m)
M10(br. s.)
M12(m)
M13(m)
M14(m)
M15(d)
M16(d)
M17(m)
M18(m)
M19(t)
M20(m)
M21(m)
M22(m)

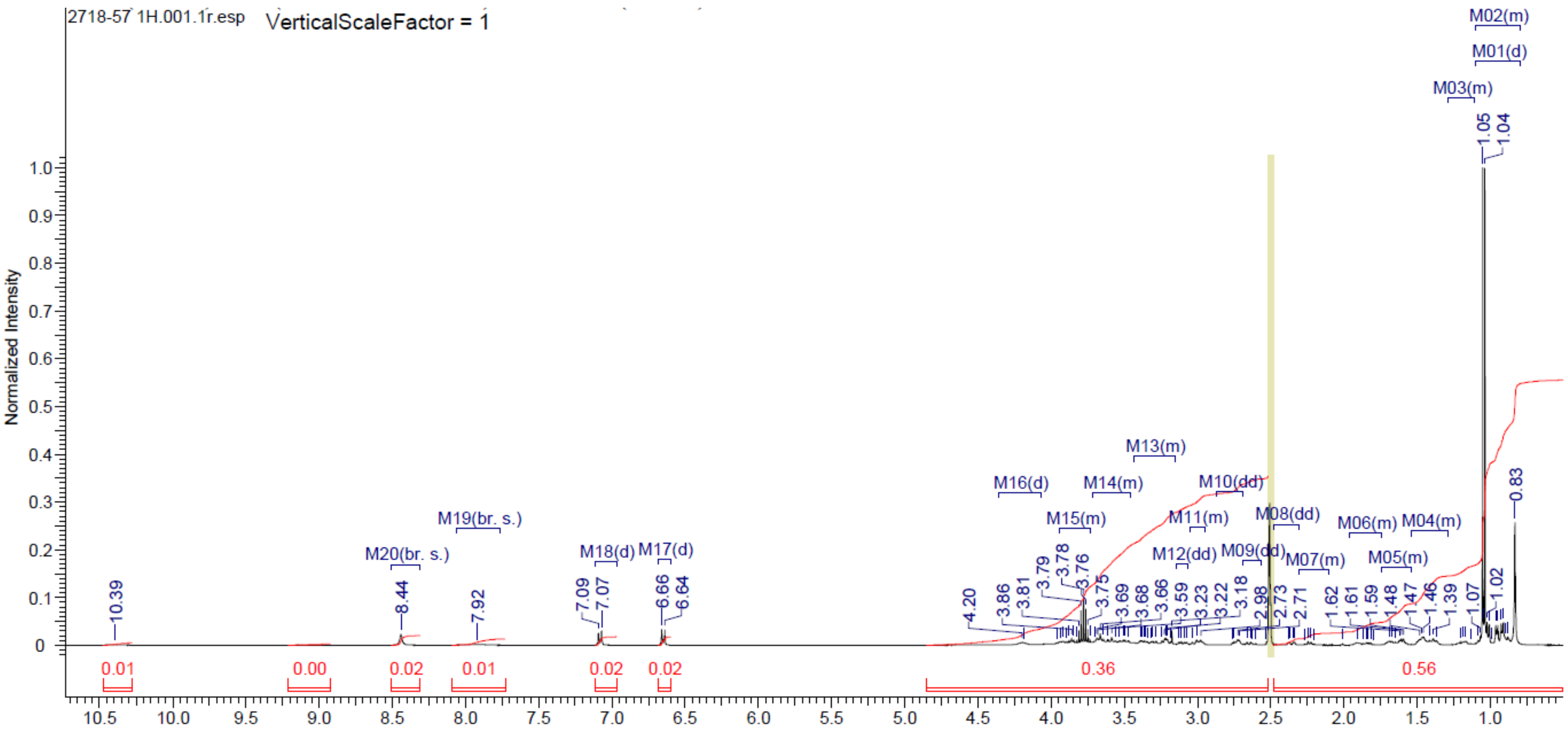

2718-57 1H.001.1r.esp
VerticalScaleFactor = 1
Normalized Intensity
M01(d)
M02(m)
M03(m)
M04(m)
M05(m)
M06(m)
M07(m)
M08(dd)
M09(dd)
M10(dd)
M11(m)
M12(dd)
M13(m)
M14(m)
M15(m)
M16(d)
M17(d)
M18(d)
M19(br. s.)
M20(br. s.)
0.01
0.00
0.02
0.01
0.02
0.02
0.36
0.56

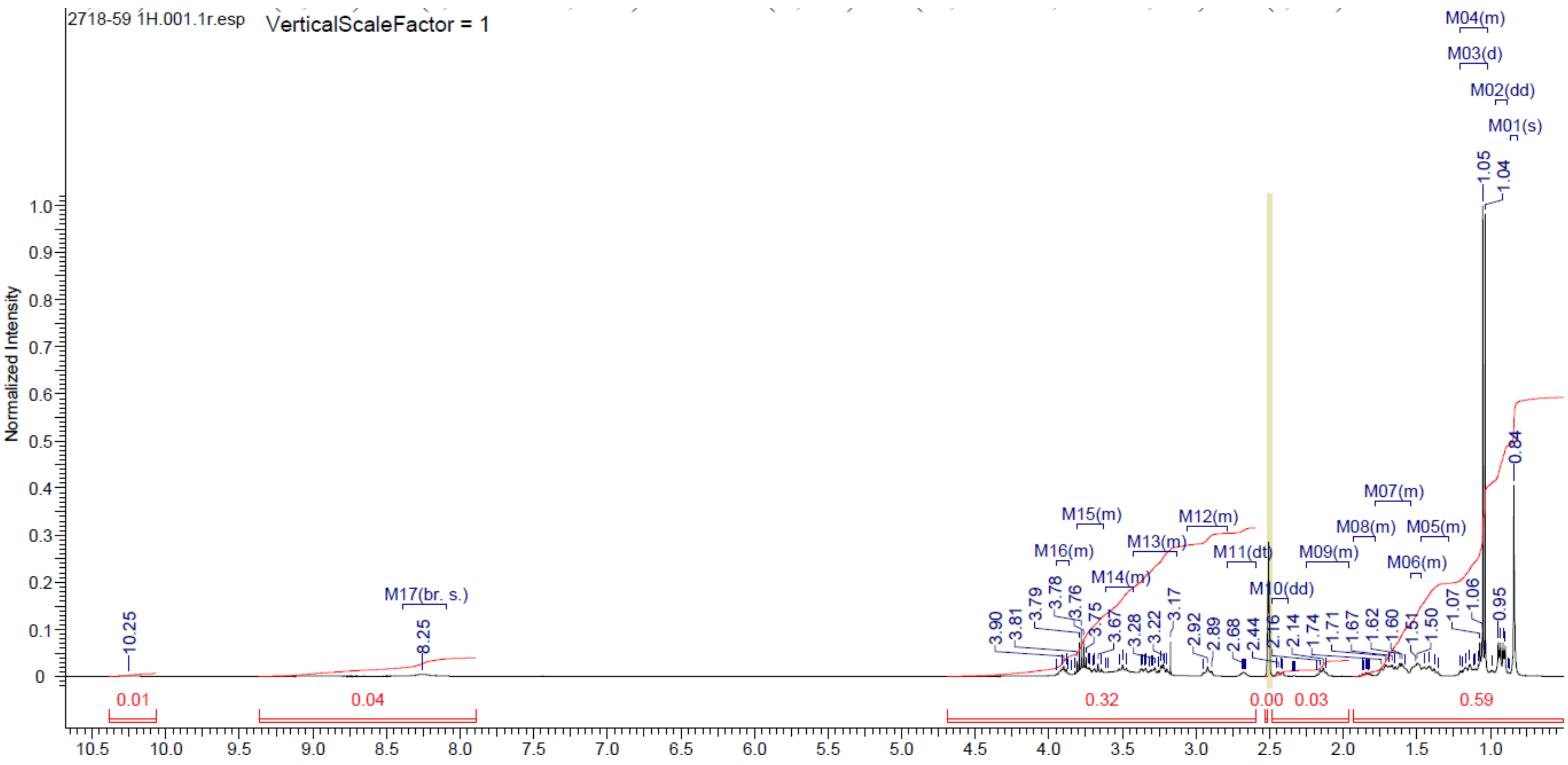

2718-59 1H.001.1r.esp
VerticalScaleFactor = 1
Normalized Intensity
M01(s)
M02(dd)
M03(d)
M04(m)
M05(m)
M06(m)
M07(m)
M08(m)
M09(m)
M10(dd)
M11(dt)
M12(m)
M13(m)
M14(m)
M15(m)
M16(m)
M17(br. s.)
0.01
0.04
0.32
0.00
0.03
0.59

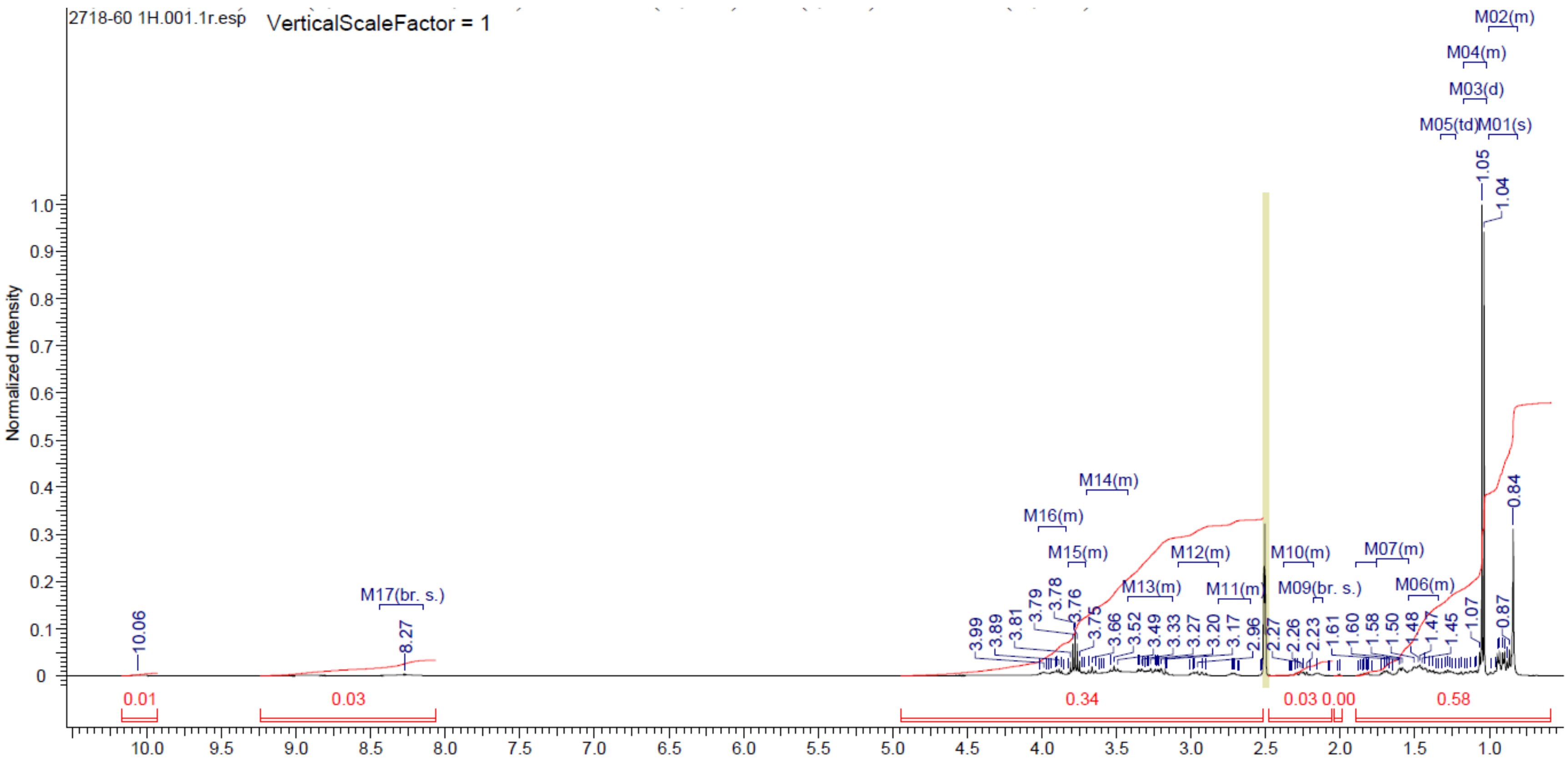

2718-60 1H.001.1r.esp
VerticalScaleFactor = 1
Normalized Intensity
M02(m)
M04(m)
M03(d)
M05(td)
M01(s)
M14(m)
M16(m)
M15(m)
M12(m)
M10(m)
M07(m)
M13(m)
M11(m)
M09(br. s.)
M06(m)
M17(br. s.)
10.06
8.27
3.99
3.89
3.81
3.79
3.78
3.76
3.75
3.66
3.52
3.49
3.33
3.27
3.20
3.17
2.96
2.27
2.26
2.23
1.61
1.60
1.58
1.50
1.48
1.47
1.45
1.07
1.05
1.04
0.87
0.84
0.01
0.03
0.34
0.03
0.00
0.58

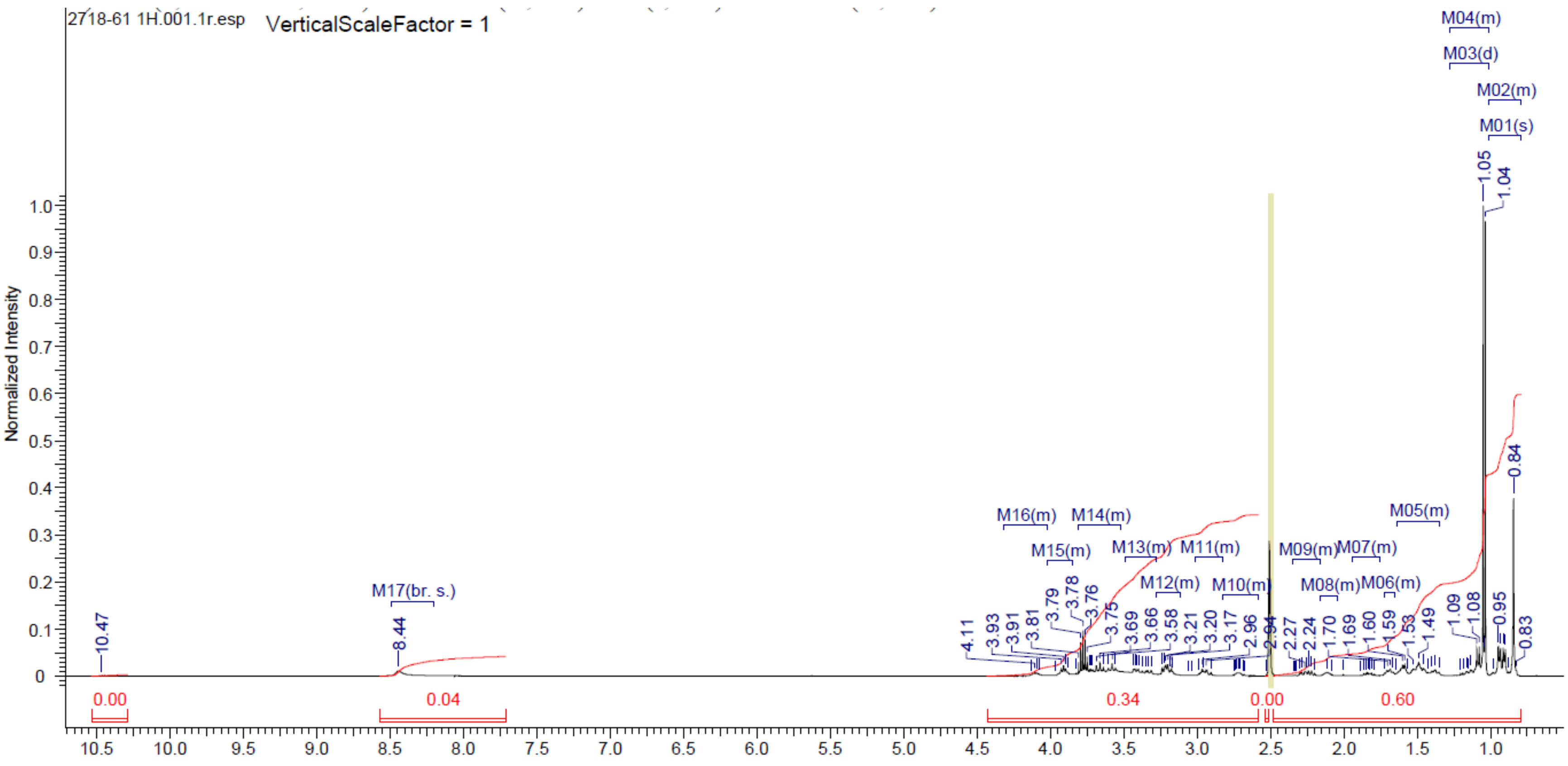

2718-61 1H.001.1r.esp
VerticalScaleFactor = 1
Normalized Intensity
M01(s)
M02(m)
M03(d)
M04(m)
M05(m)
M06(m)
M07(m)
M08(m)
M09(m)
M10(m)
M11(m)
M12(m)
M13(m)
M14(m)
M15(m)
M16(m)
M17(br. s.)
10.47
8.44
4.11
3.93
3.91
3.81
3.79
3.78
3.76
3.75
3.69
3.66
3.58
3.21
3.20
3.17
2.96
2.94
2.27
2.24
1.70
1.69
1.60
1.59
1.53
1.49
1.09
1.08
1.05
1.04
0.95
0.84
0.83
0.00
0.04
0.34
0.00
0.60
